# Supplementary material for: Real-time forecasting the trajectory of monkeypox outbreaks at the national and global levels, July–October 2022
Source: BMC Med. 2023 Jan 16;21:19. doi: 10.1186/s12916-022-02725-2 (PMC9841951; doi:10.1186/s12916-022-02725-2)
Supplement: Supplementary file 1 — Additional file 1: Supplementary figures. Additional figures related to the dissemination and evaluation of models used to produce the forecasts included within the study. [file 12916_2022_2725_MOESM1_ESM.pdf]

Additional file 1: Supplementary figures

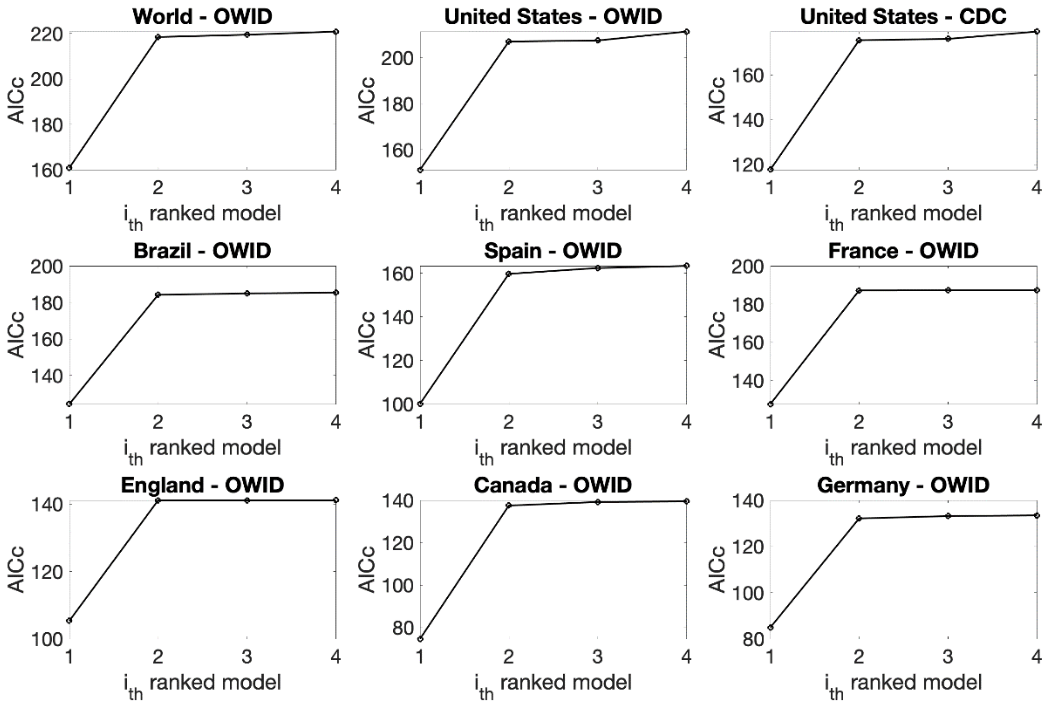

Figure 1s. AICc values of the top sub-epidemic models for the latest forecasts produced during the week of October 13th, 2022.

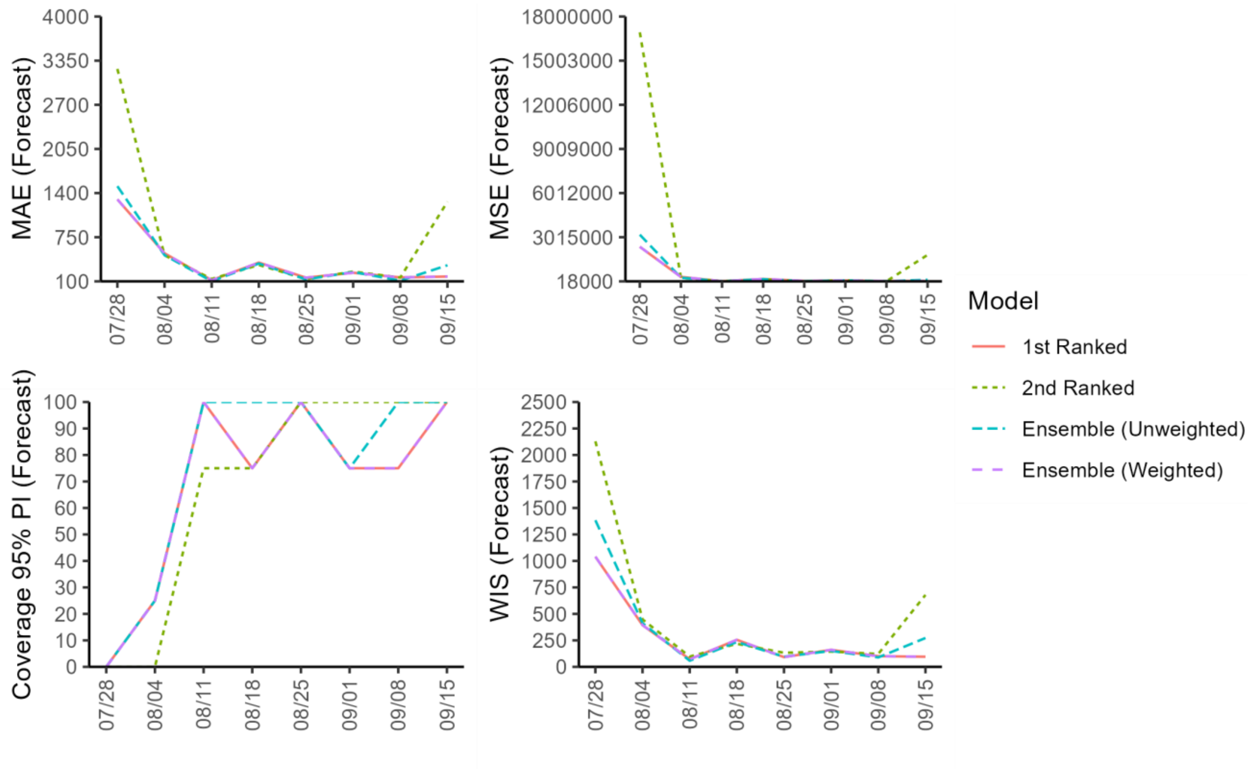

Figure 2s. Performance metrics of the forecasts generated by the sub-epidemic models across 8 sequential forecasting periods (Week of July 28th through the week of September 15th, 2022) for Brazil.

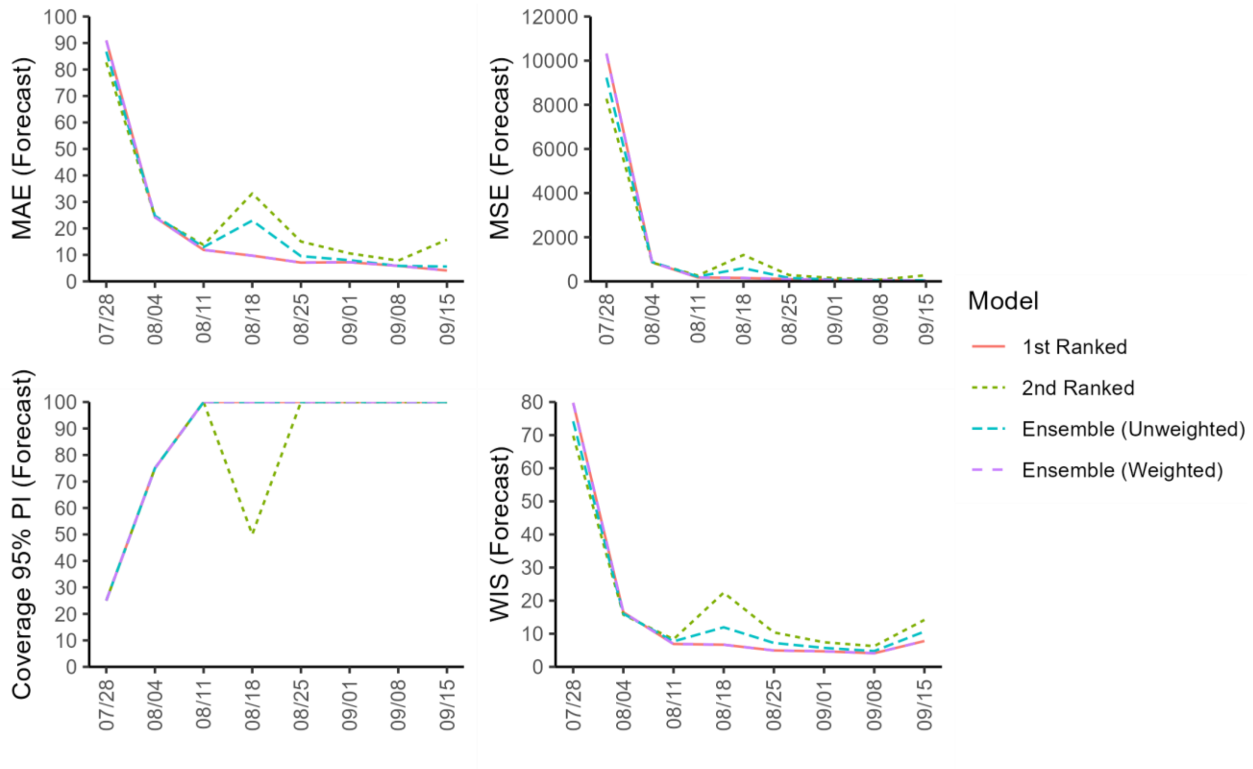

Figure 3s. Performance metrics of the forecasts generated by the sub-epidemic models across 8 sequential forecasting periods (Week of July 28th through the week of September 15th, 2022) for Canada.

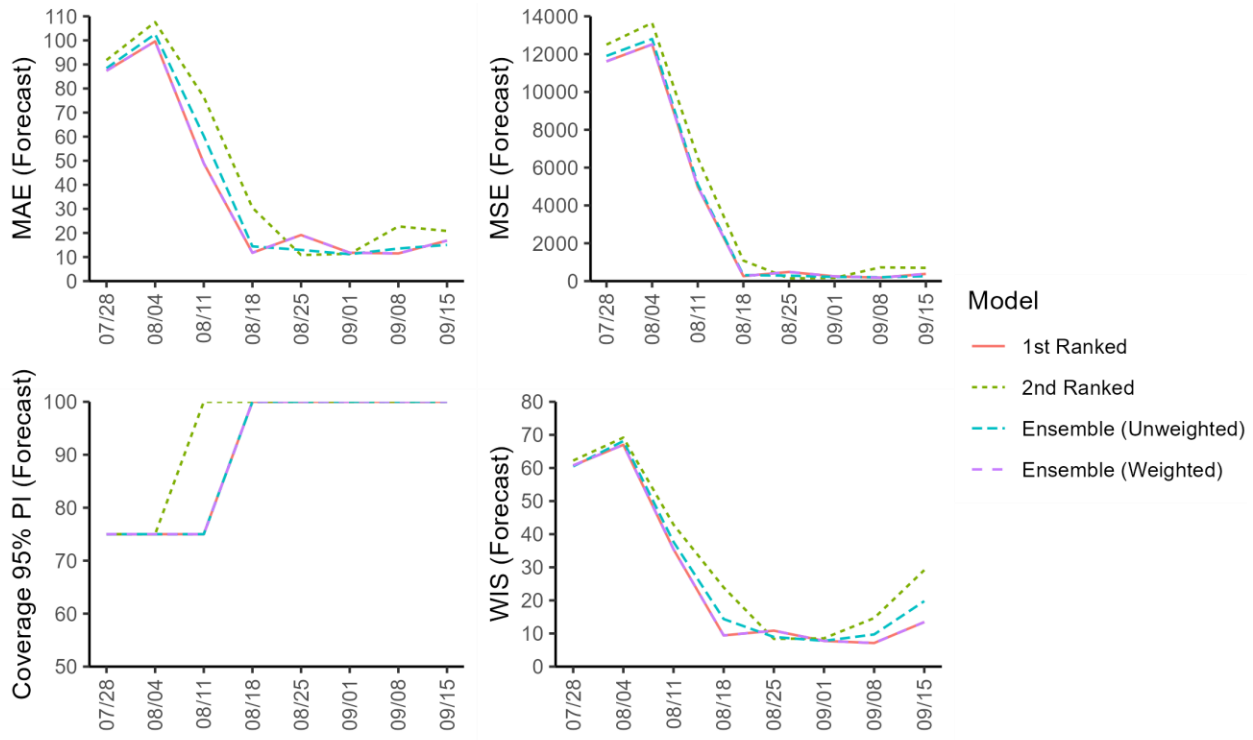

Figure 4s. Performance metrics of the forecasts generated by the sub-epidemic models across 8 sequential forecasting periods (Week of July 28th through the week of September 15th, 2022) for England.

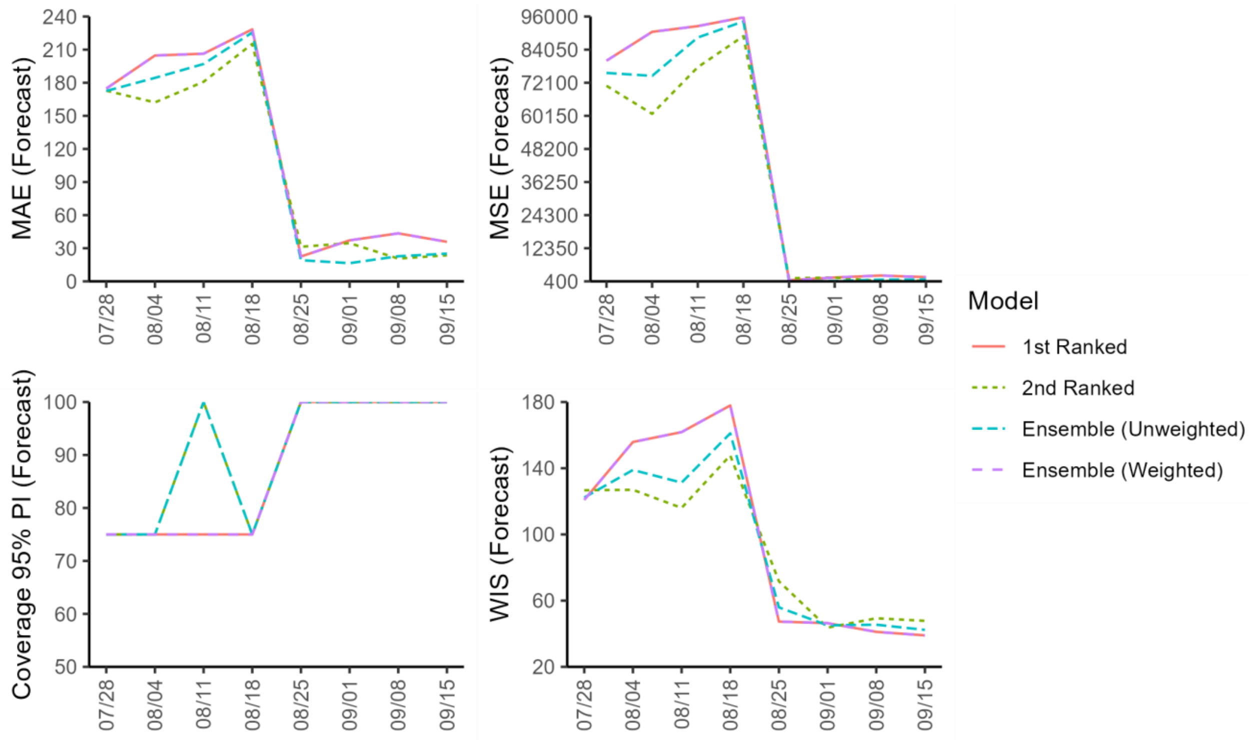

Figure 5s. Performance metrics of the forecasts generated by the sub-epidemic models across 8 sequential forecasting periods (Week of July 28th through the week of September 15th, 2022) for France.

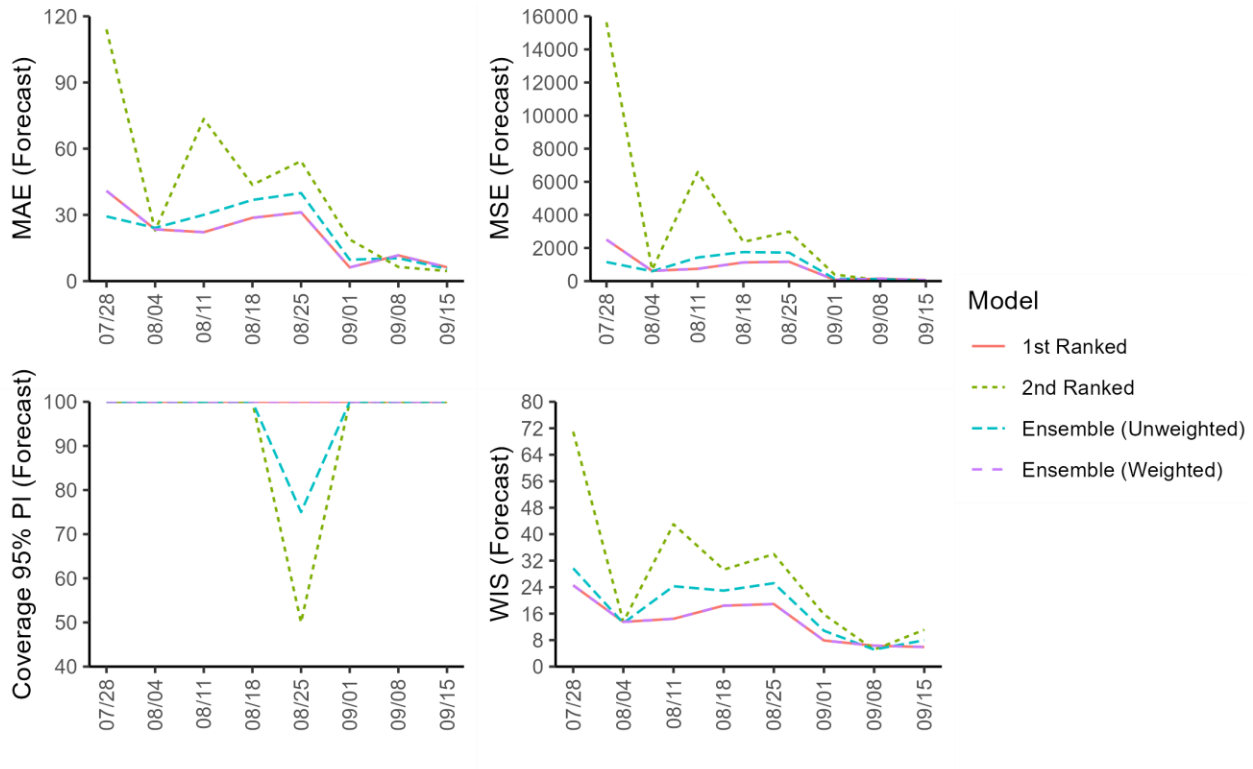

Figure 6s. Performance metrics of the forecasts generated by the sub-epidemic models across 8 sequential forecasting periods (Week of July 28th through the week of September 15th, 2022) for Germany.

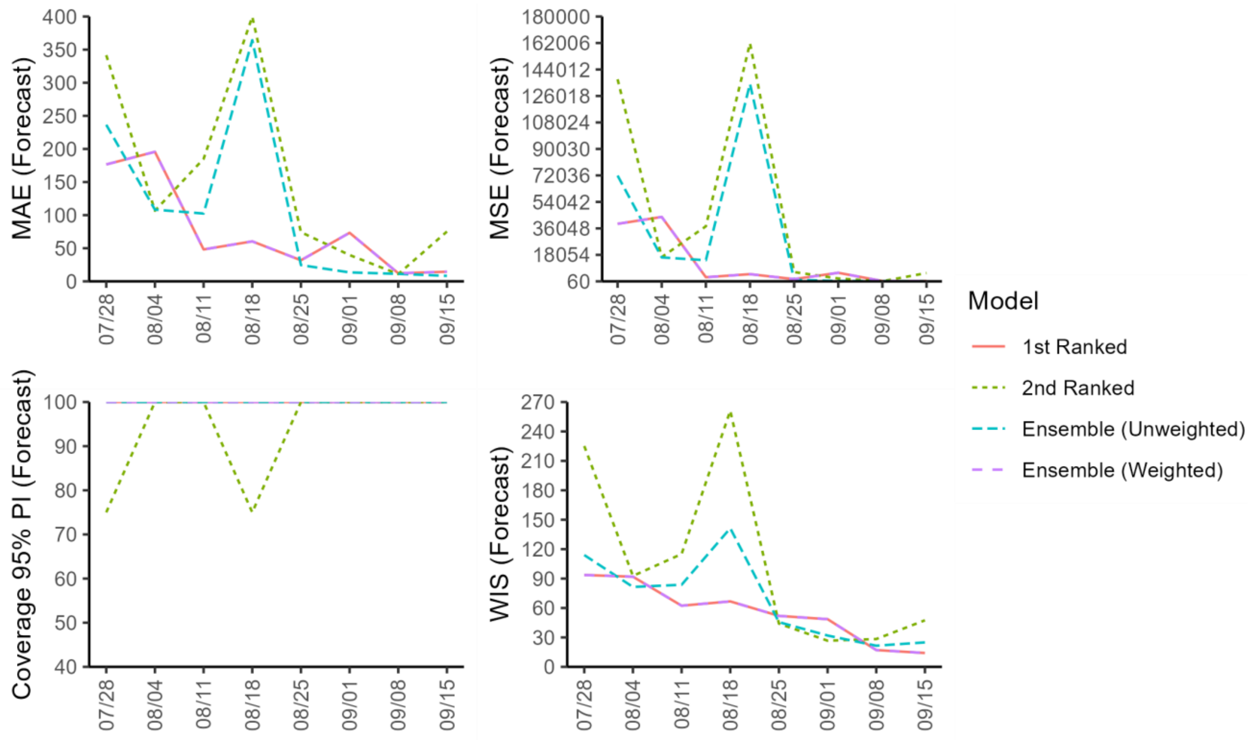

Figure 7s. Performance metrics of the forecasts generated by the sub-epidemic models across 8 sequential forecasting periods (Week of July 28th through the week of September 15th, 2022) for Spain.

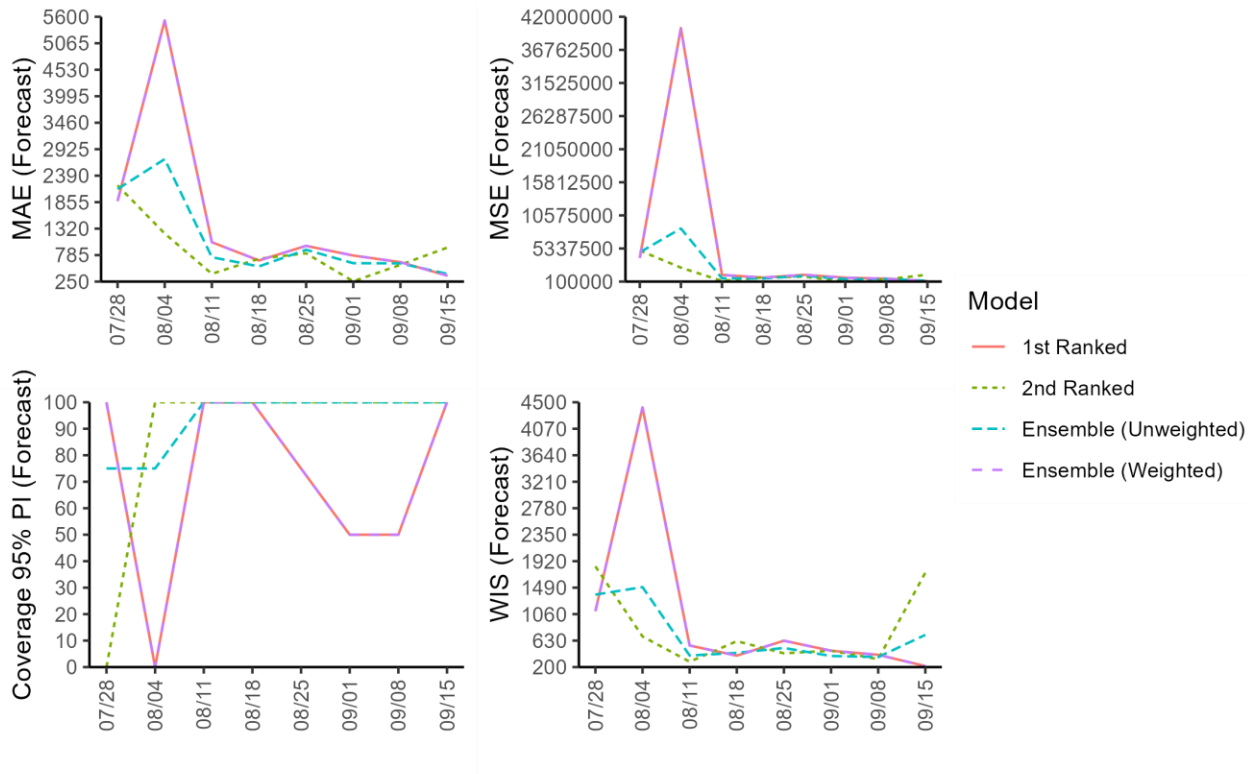

Figure 8s. Performance metrics of the forecasts generated by the sub-epidemic models across 8 sequential forecasting periods (Week of July 28th through the week of September 15th, 2022) for the United States (OWID).

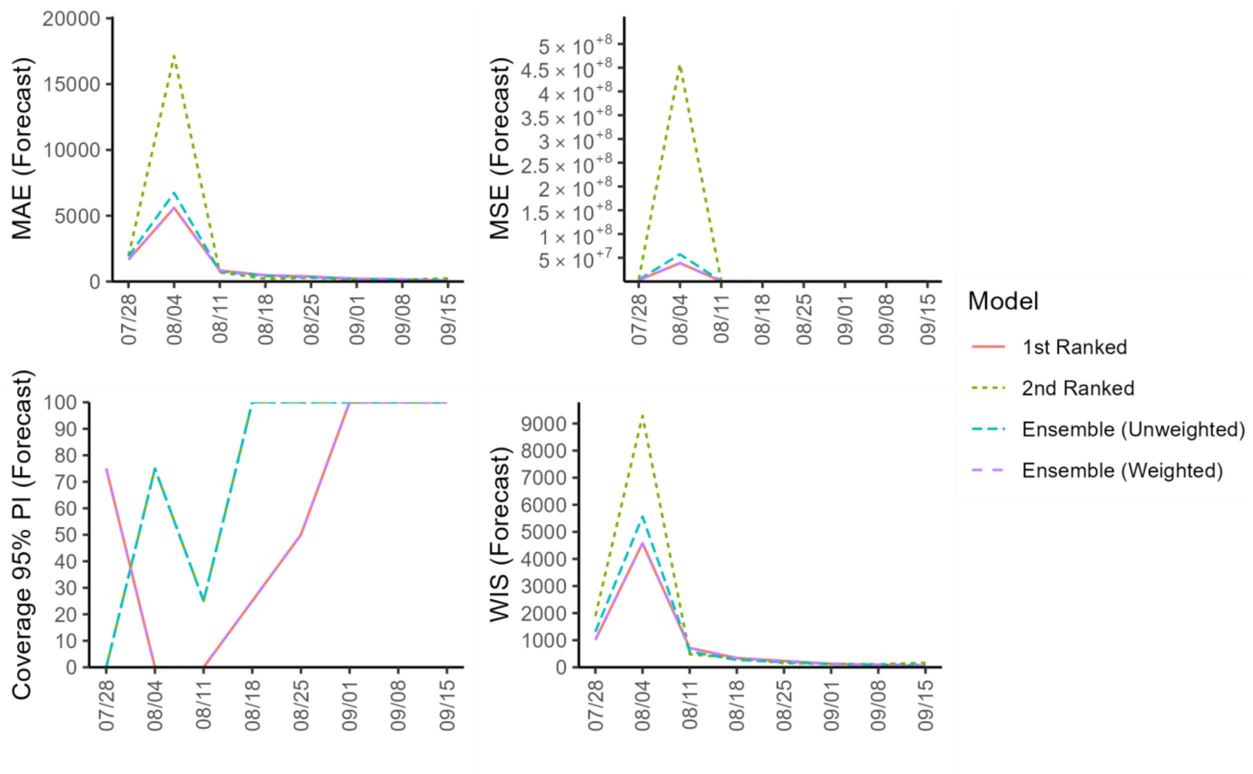

Figure 9s. Performance metrics of the forecasts generated by the sub-epidemic models across 8 sequential forecasting periods (Week of July 28th through the week of September 15th, 2022) for the United States (CDC).

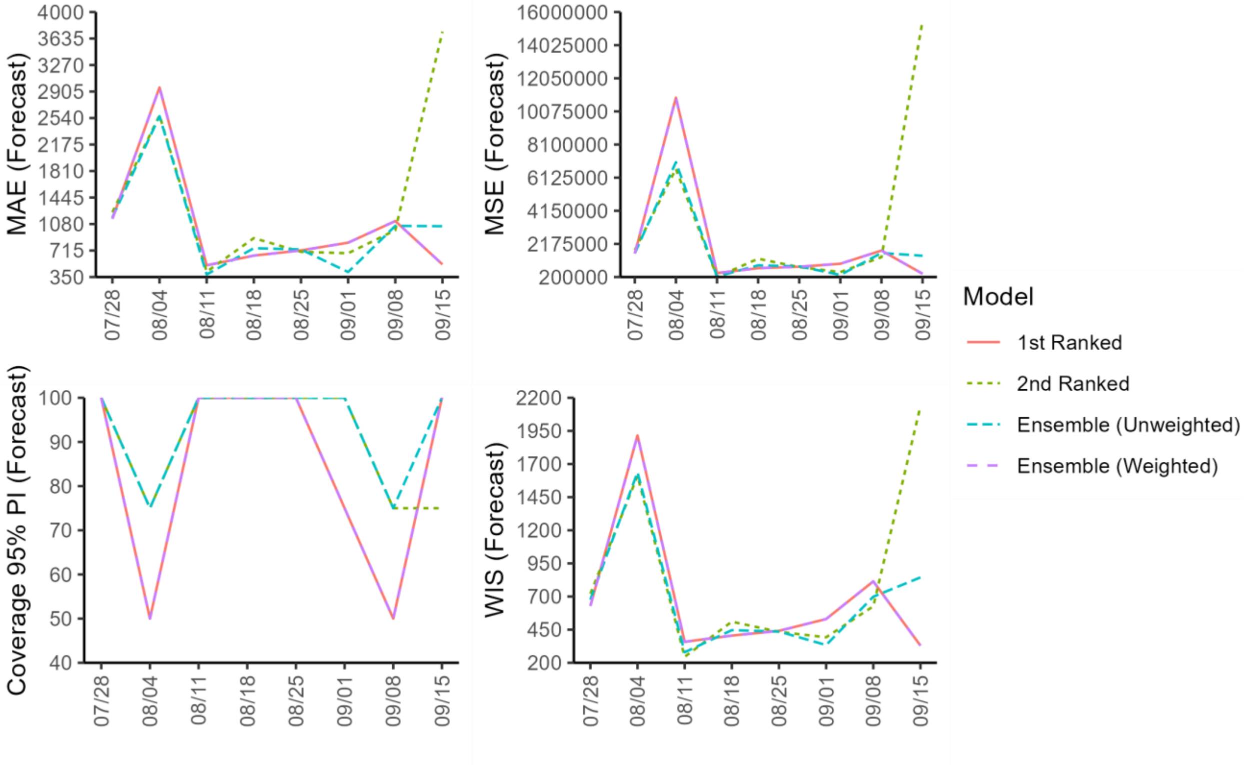

Figure 10s. Performance metrics of the forecasts generated by the sub-epidemic models across 8 sequential forecasting periods (Week of July 28th through the week of September 15th, 2022) for the World.

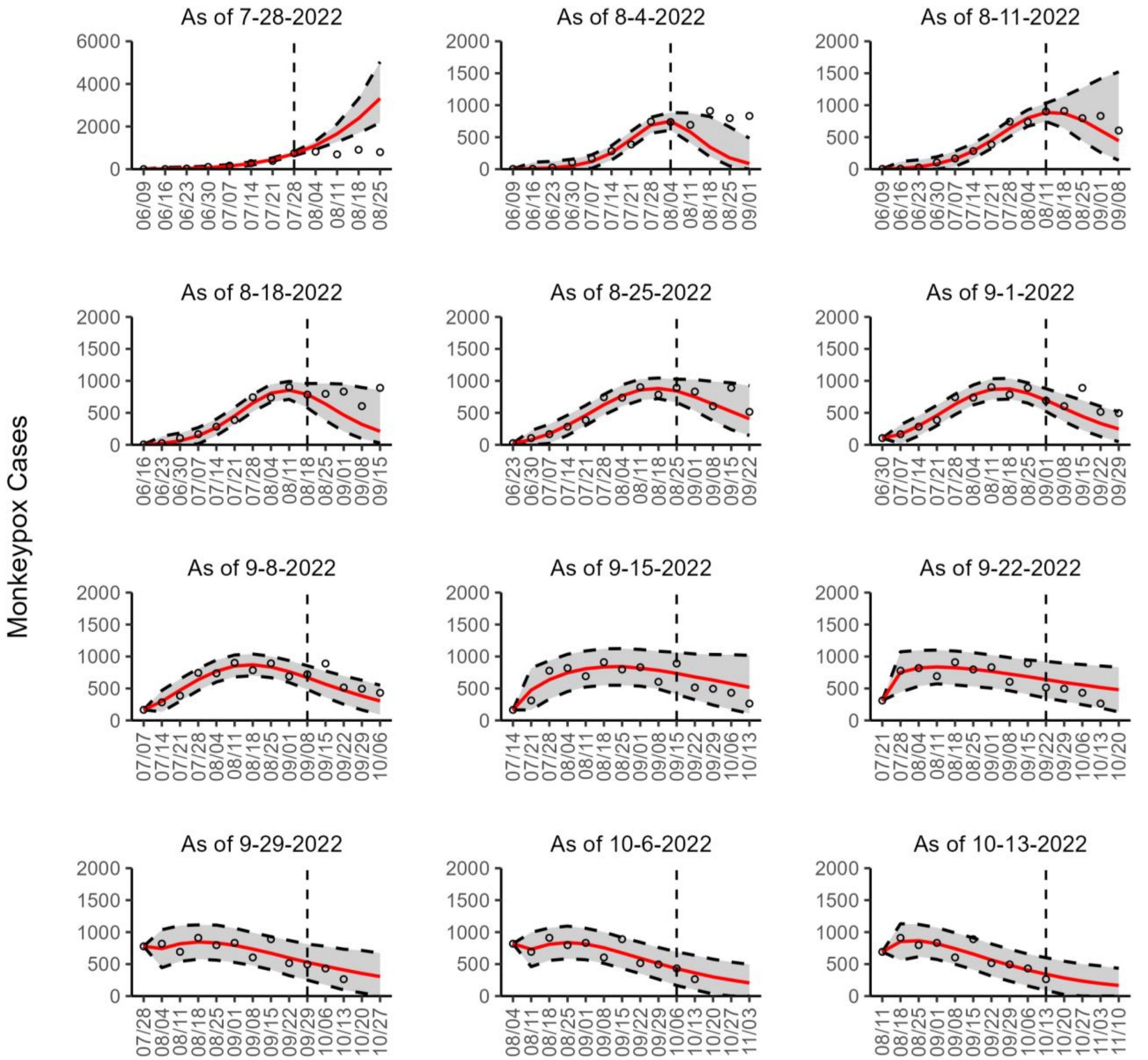

Figure 11s. The overlayed forecasted and reported monkeypox cases for the weeks of 7/28/2022 through the week of 10/13/2022 for Brazil. The forecasts are derived from the top-ranked sub-epidemic model using 10-week calibration data, and the reported cases are obtained from the OWID GitHub [25]. The black circles to the left of the vertical line represent the reported cases as of the Friday of the forecast period; the solid red line corresponds to the best fit model; the dashed black lines correspond to the 95% prediction intervals. The black circles to the right of the vertical line represent the reported case counts (as of 10/21/2022) for the corresponding date. The vertical dashed black line indicates the start of the forecast period. For the week of 7/28/2022, data posted by the OWID team on 8/9/2022 was used to produce the forecast as it was the earliest version of data available.

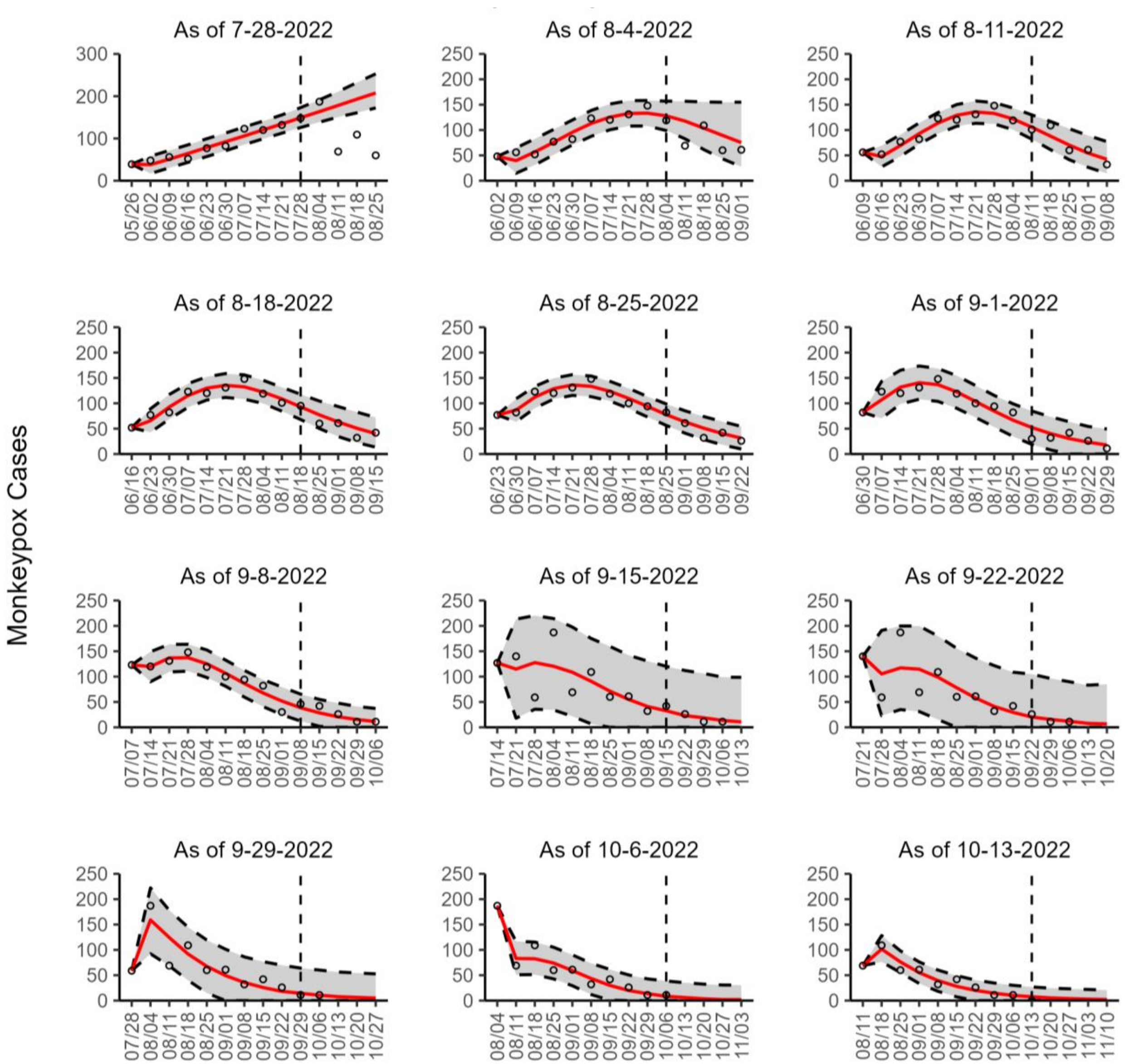

Figure 12s. The overlayed forecasted and reported monkeypox cases for the weeks of 7/28/2022 through the week of 10/13/2022 for Canada. The forecasts are derived from the top-ranked sub-epidemic model using 10-week calibration data, and the reported cases are obtained from the OWID GitHub [25]. The black circles to the left of the vertical line represent the reported cases as of the Friday of the forecast period; the solid red line corresponds to the best fit model; the dashed black lines correspond to the 95% prediction intervals. The black circles to the right of the vertical line represent the reported case counts (as of 10/21/2022) for the corresponding date. The vertical dashed black line indicates the start of the forecast period. For the week of 7/28/2022, data posted by the OWID team on 8/9/2022 was used to produce the forecast as it was the earliest version of data available.

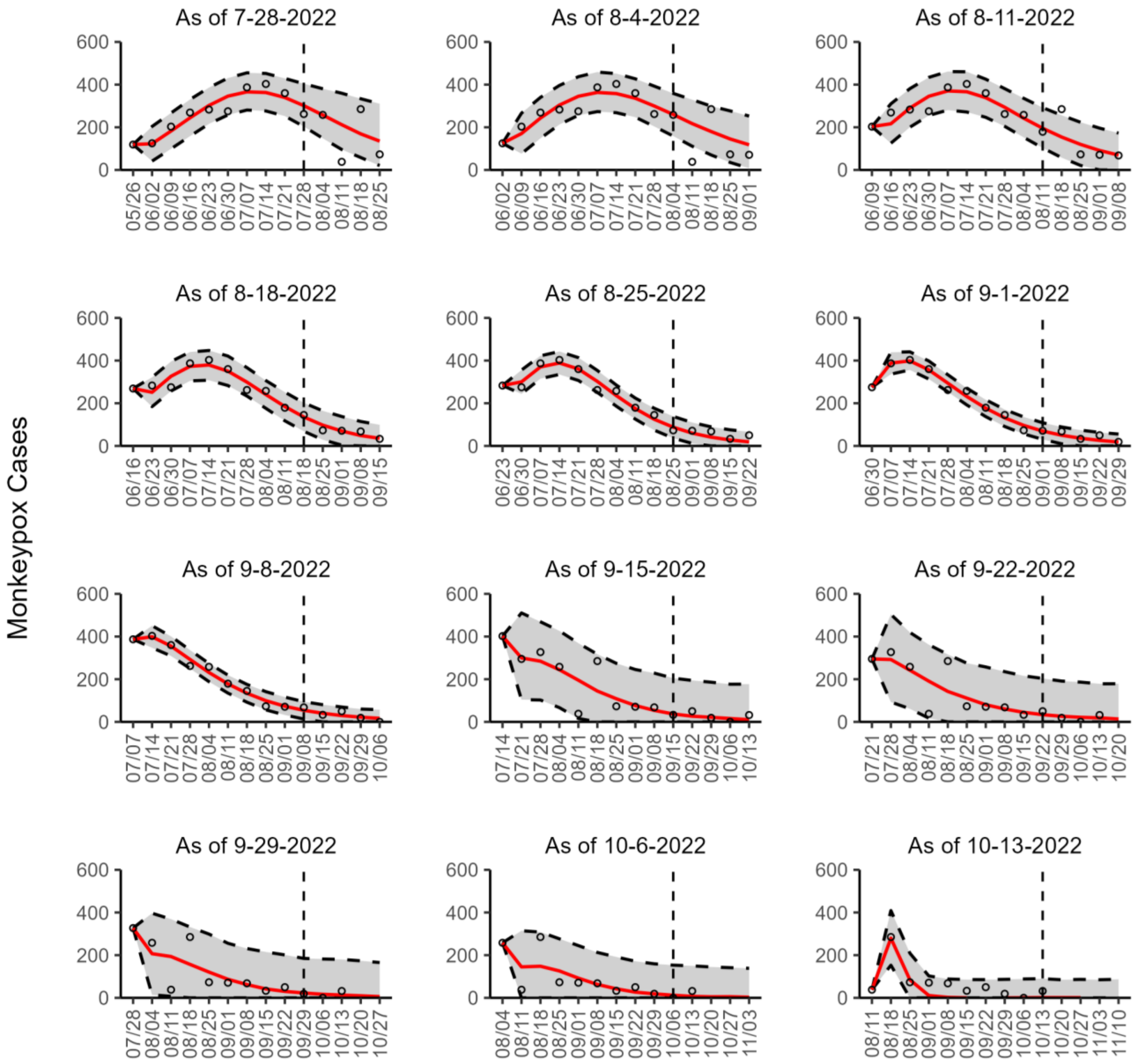

Figure 13s. The overlayed forecasted and reported monkeypox cases for the weeks of 7/28/2022 through the week of 10/13/2022 for England. The forecasts are derived from the top-ranked sub-epidemic model using 10-week calibration data, and the reported cases are obtained from the OWID GitHub [25]. The black circles to the left of the vertical line represent the reported cases as of the Friday of the forecast period; the solid red line corresponds to the best fit model; the dashed black lines correspond to the 95% prediction intervals. The black circles to the right of the vertical line represent the reported case counts (as of 10/21/2022) for the corresponding date. The vertical dashed black line indicates the start of the forecast period. For the week of 7/28/2022, data posted by the OWID team on 8/9/2022 was used to produce the forecast as it was the earliest version of data available.

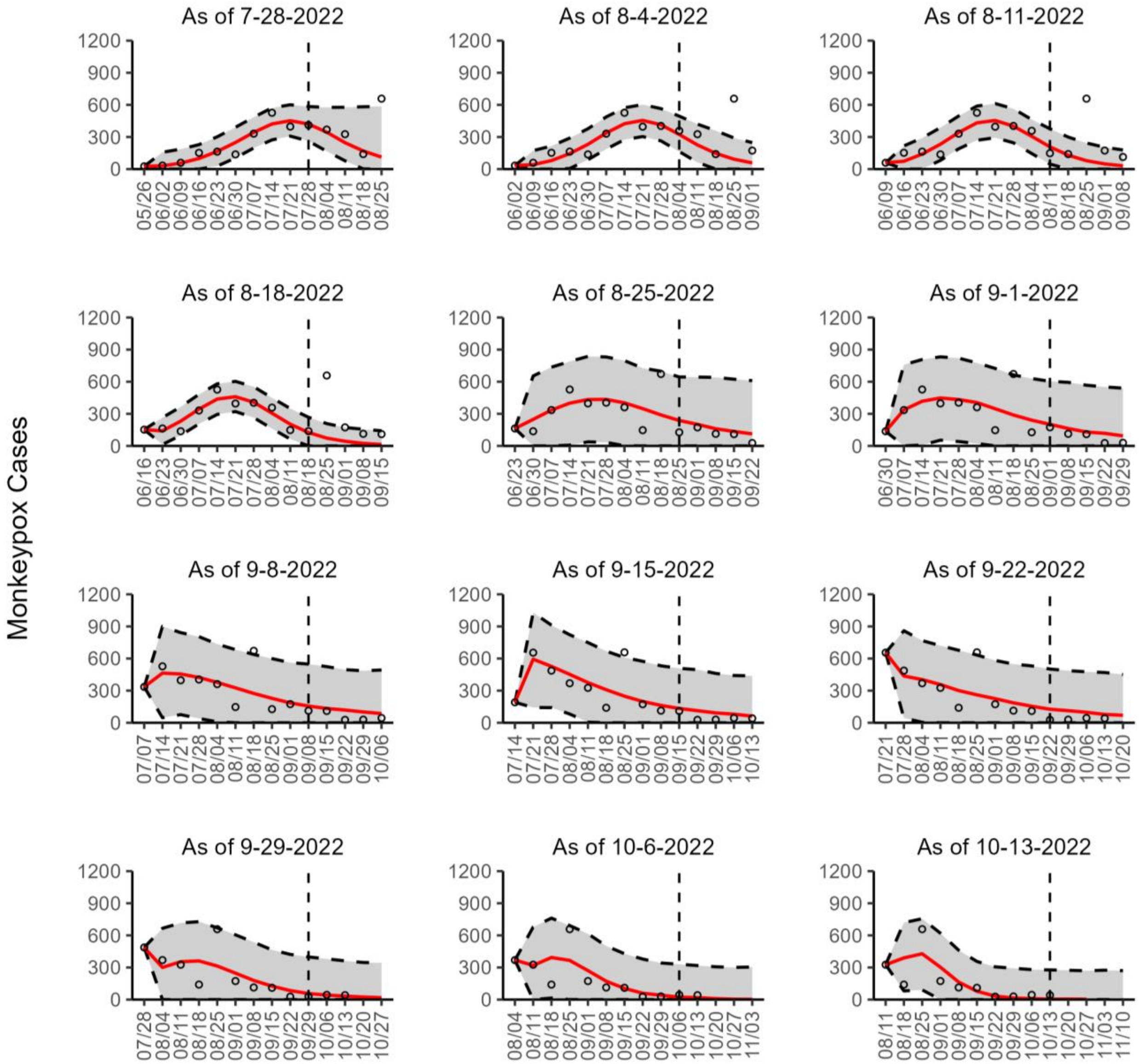

Figure 14s. The overlayed forecasted and reported monkeypox cases for the weeks of 7/28/2022 through the week of 10/13/2022 for France. The forecasts are derived from the top-ranked sub-epidemic model using 10-week calibration data, and the reported cases are obtained from the OWID GitHub [25]. The black circles to the left of the vertical line represent the reported cases as of the Friday of the forecast period; the solid red line corresponds to the best fit model; the dashed black lines correspond to the 95% prediction intervals. The black circles to the right of the vertical line represent the reported case counts (as of 10/21/2022) for the corresponding date. The vertical dashed black line indicates the start of the forecast period. For the week of 7/28/2022, data posted by the OWID team on 8/9/2022 was used to produce the forecast as it was the earliest version of data available.

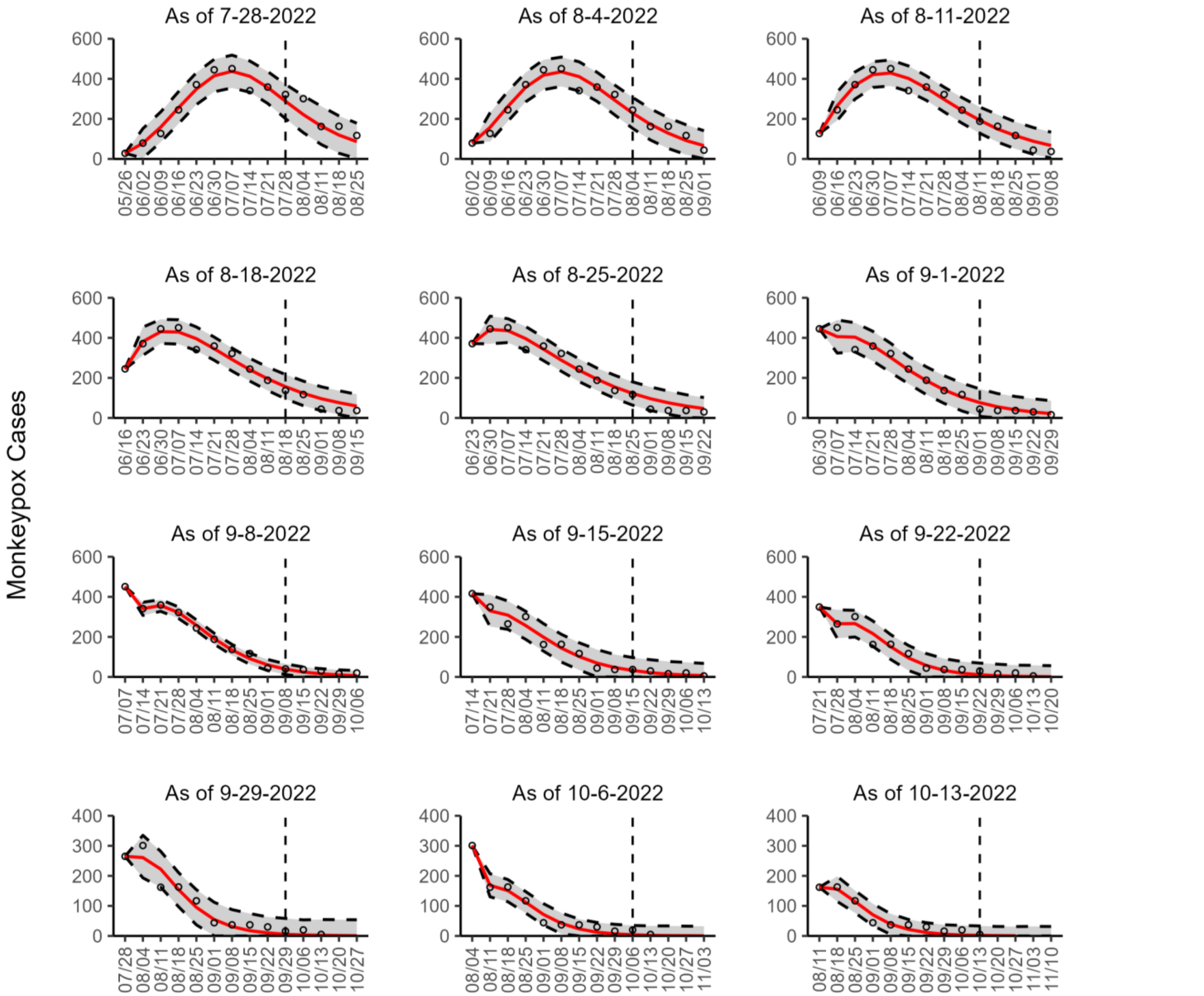

Figure 15s. The overlayed forecasted and reported monkeypox cases for the weeks of 7/28/2022 through the week of 10/13/2022 for Germany. The forecasts are derived from the top-ranked sub-epidemic model using 10-week calibration data, and the reported cases are obtained from the OWID GitHub [25]. The black circles to the left of the vertical line represent the reported cases as of the Friday of the forecast period; the solid red line corresponds to the best fit model; the dashed black lines correspond to the 95% prediction intervals. The black circles to the right of the vertical line represent the reported case counts (as of 10/21/2022) for the corresponding date. The vertical dashed black line indicates the start of the forecast period. For the week of 7/28/2022, data posted by the OWID team on 8/9/2022 was used to produce the forecast as it was the earliest version of data available.

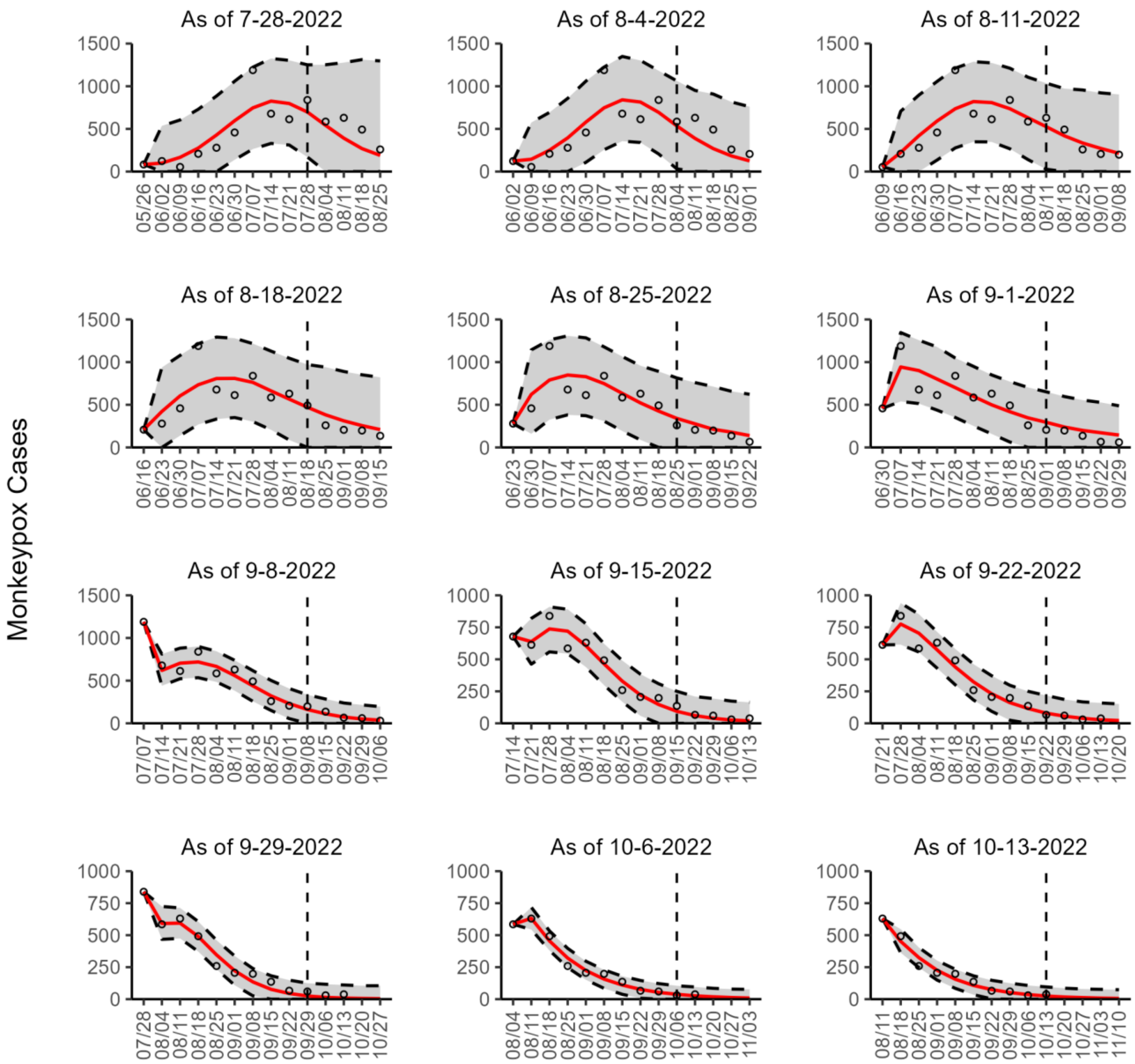

Figure 16s. The overlayed forecasted and reported monkeypox cases for the weeks of 7/28/2022 through the week of 10/13/2022 for Spain. The forecasts are derived from the top-ranked sub-epidemic model using 10-week calibration data, and the reported cases are obtained from the OWID GitHub [25]. The black circles to the left of the vertical line represent the reported cases as of the Friday of the forecast period; the solid red line corresponds to the best fit model; the dashed black lines correspond to the 95% prediction intervals. The black circles to the right of the vertical line represent the reported case counts (as of 10/21/2022) for the corresponding date. The vertical dashed black line indicates the start of the forecast period. For the week of 7/28/2022, data posted by the OWID team on 8/9/2022 was used to produce the forecast as it was the earliest version of data available.

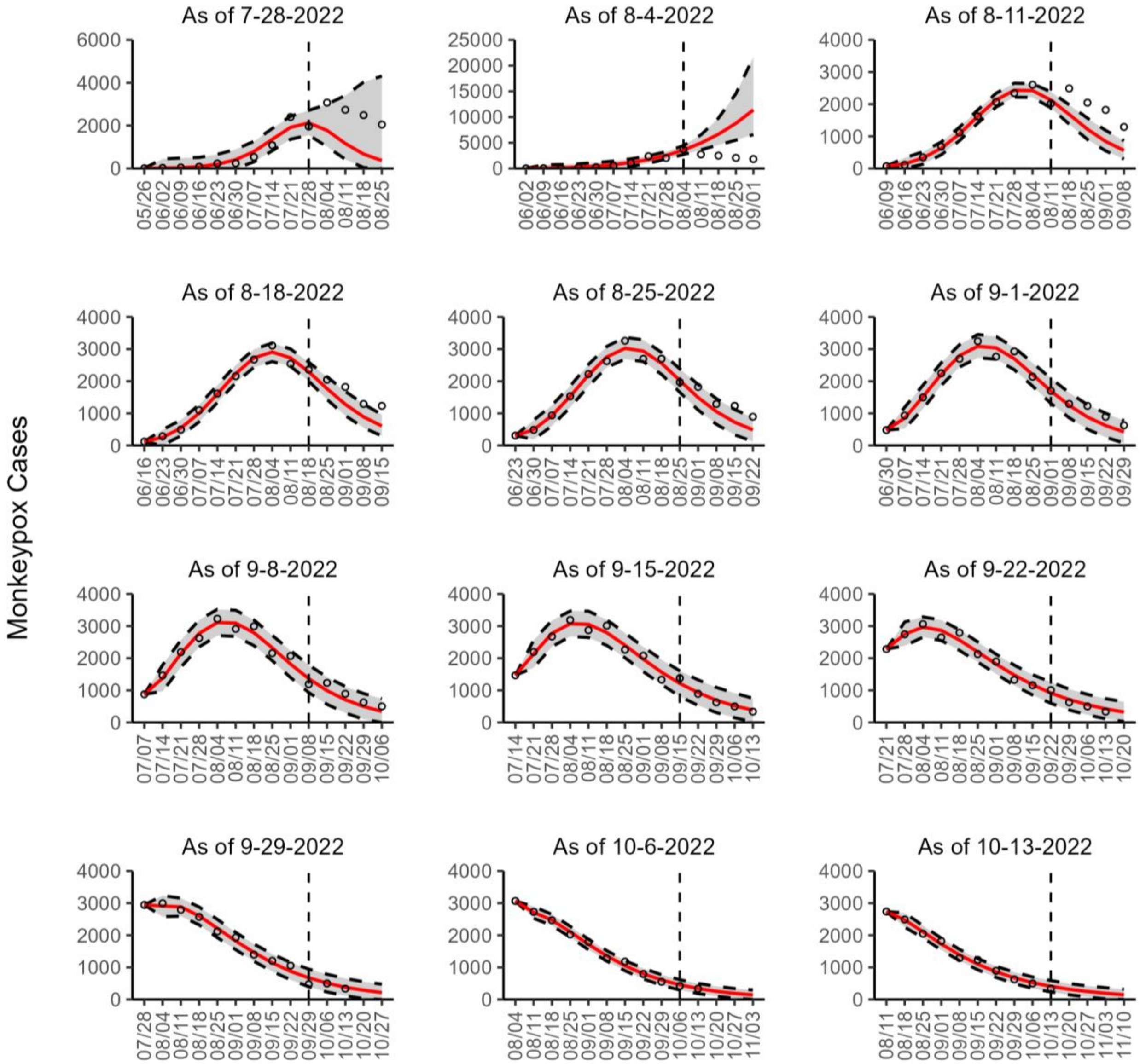

Figure 17s. The overlayed forecasted and reported monkeypox cases for the weeks of 7/28/2022 through the week of 10/13/2022 for the United States. The forecasts are derived from the top-ranked sub-epidemic model using 10-week calibration data, and the reported cases are obtained from the CDC [24]. The black circles to the left of the vertical line represent the reported cases as of the Wednesday of the forecast period; the solid red line corresponds to the best fit model; the dashed black lines correspond to the 95% prediction intervals. The black circles to the right of the vertical line represent the reported case counts (as of 10/19/2022) for the corresponding date.

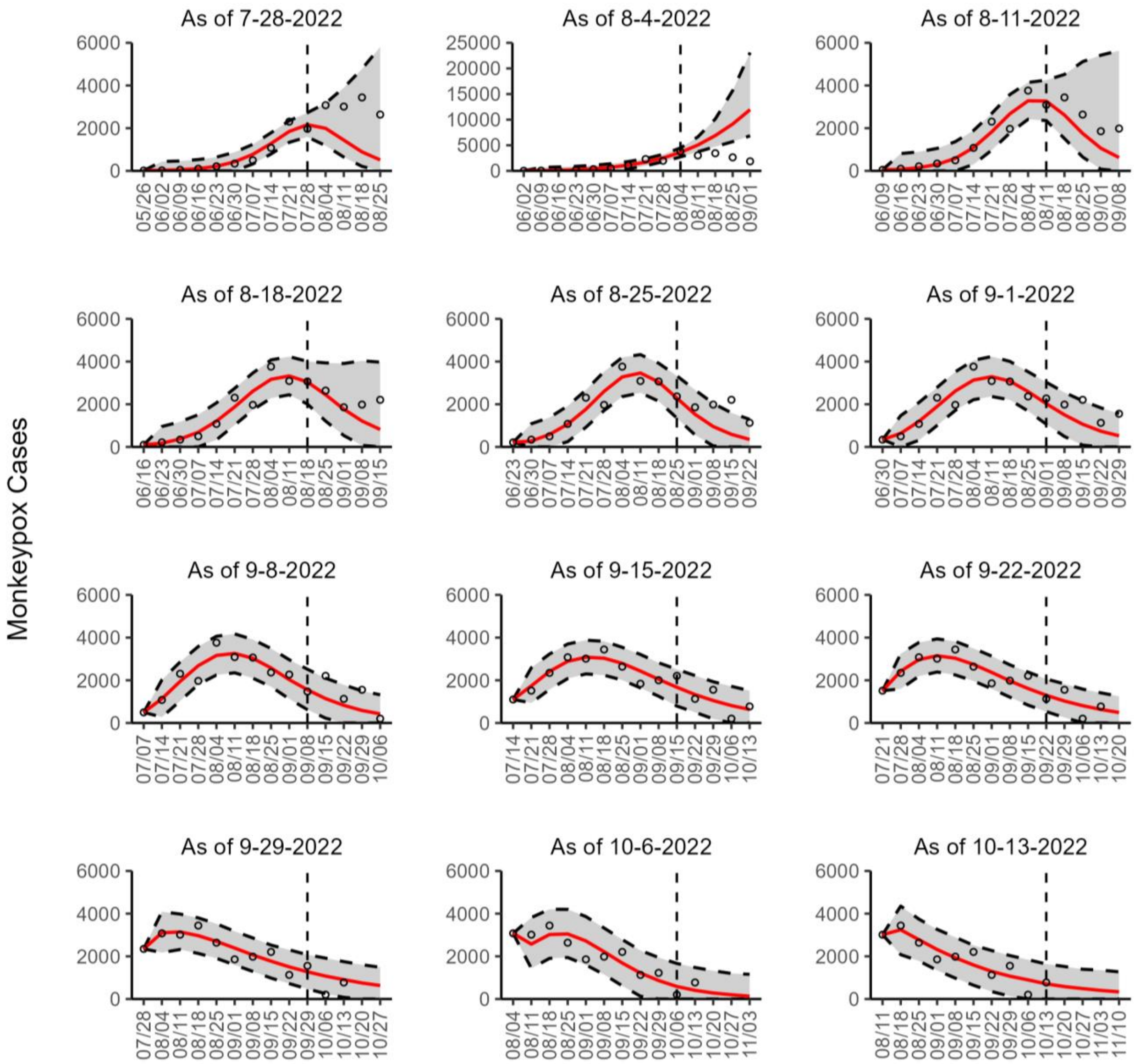

Figure 18s. The overlayed forecasted and reported monkeypox cases for the weeks of 7/28/2022 through the week of 10/13/2022 for the United States. The forecasts are derived from the top-ranked sub-epidemic model using 10-week calibration data, and the reported cases are obtained from the OWID GitHub [25]. The black circles to the left of the vertical line represent the reported cases as of the Friday of the forecast period; the solid red line corresponds to the best fit model; the dashed black lines correspond to the 95% prediction intervals. The black circles to the right of the vertical line represent the reported case counts (as of 10/21/2022) for the corresponding date. The vertical dashed black line indicates the start of the forecast period. For the week of 7/28/2022, data posted by the OWID team on 8/9/2022 was used to produce the forecast as it was the earliest version of data available.

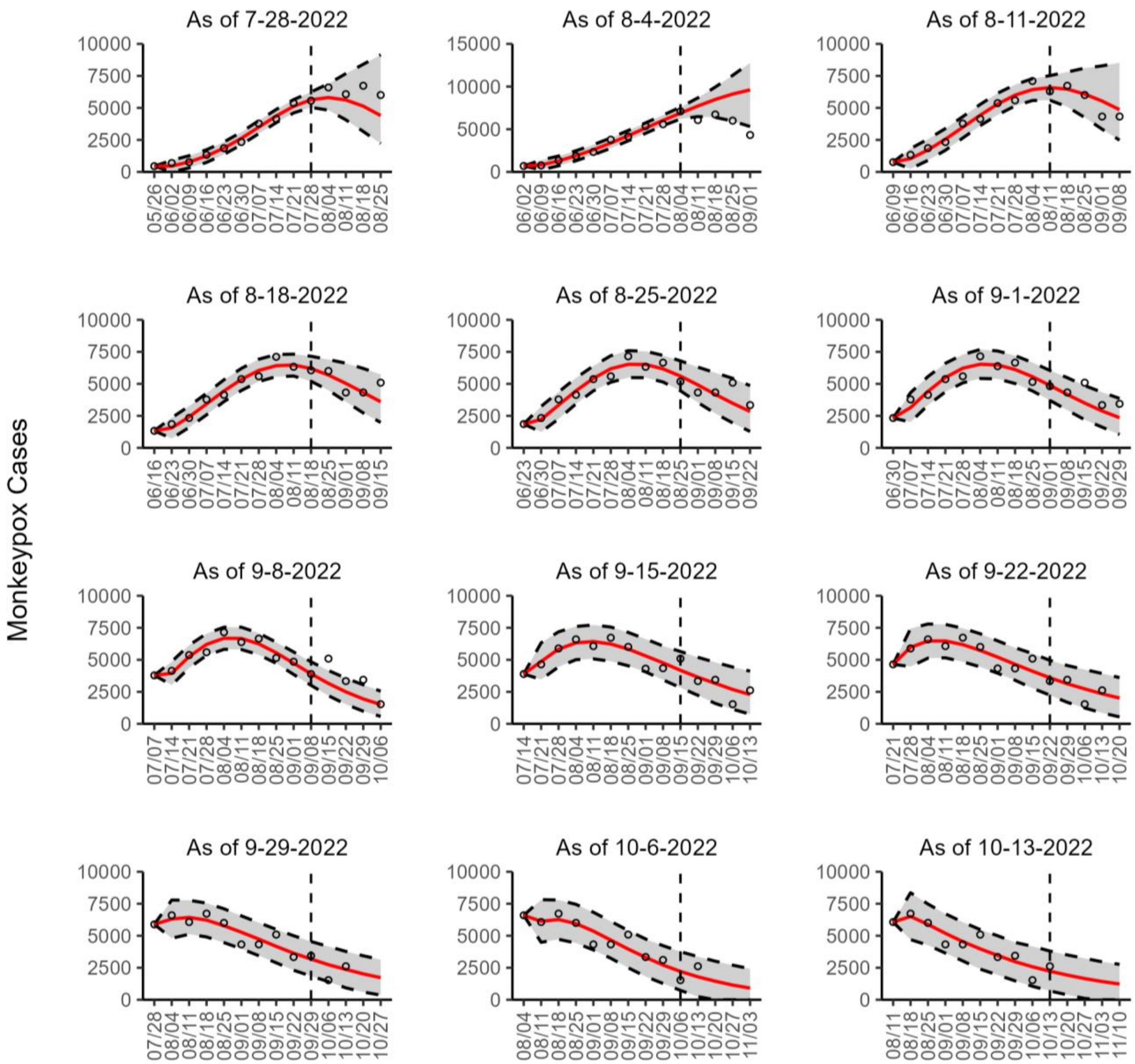

Figure 19s. The overlayed forecasted and reported monkeypox cases for the weeks of 7/28/2022 through the week of 10/13/2022 for the World. The forecasts are derived from the top-ranked sub-epidemic model using 10-week calibration data, and the reported cases are obtained from the OWID GitHub [25]. The black circles to the left of the vertical line represent the reported cases as of the Friday of the forecast period; the solid red line corresponds to the best fit model; the dashed black lines correspond to the 95% prediction intervals. The black circles to the right of the vertical line represent the reported case counts (as of 10/21/2022) for the corresponding date. The vertical dashed black line indicates the start of the forecast period. For the week of 7/28/2022, data posted by the OWID team on 8/9/2022 was used to produce the forecast as it was the earliest version of data available.

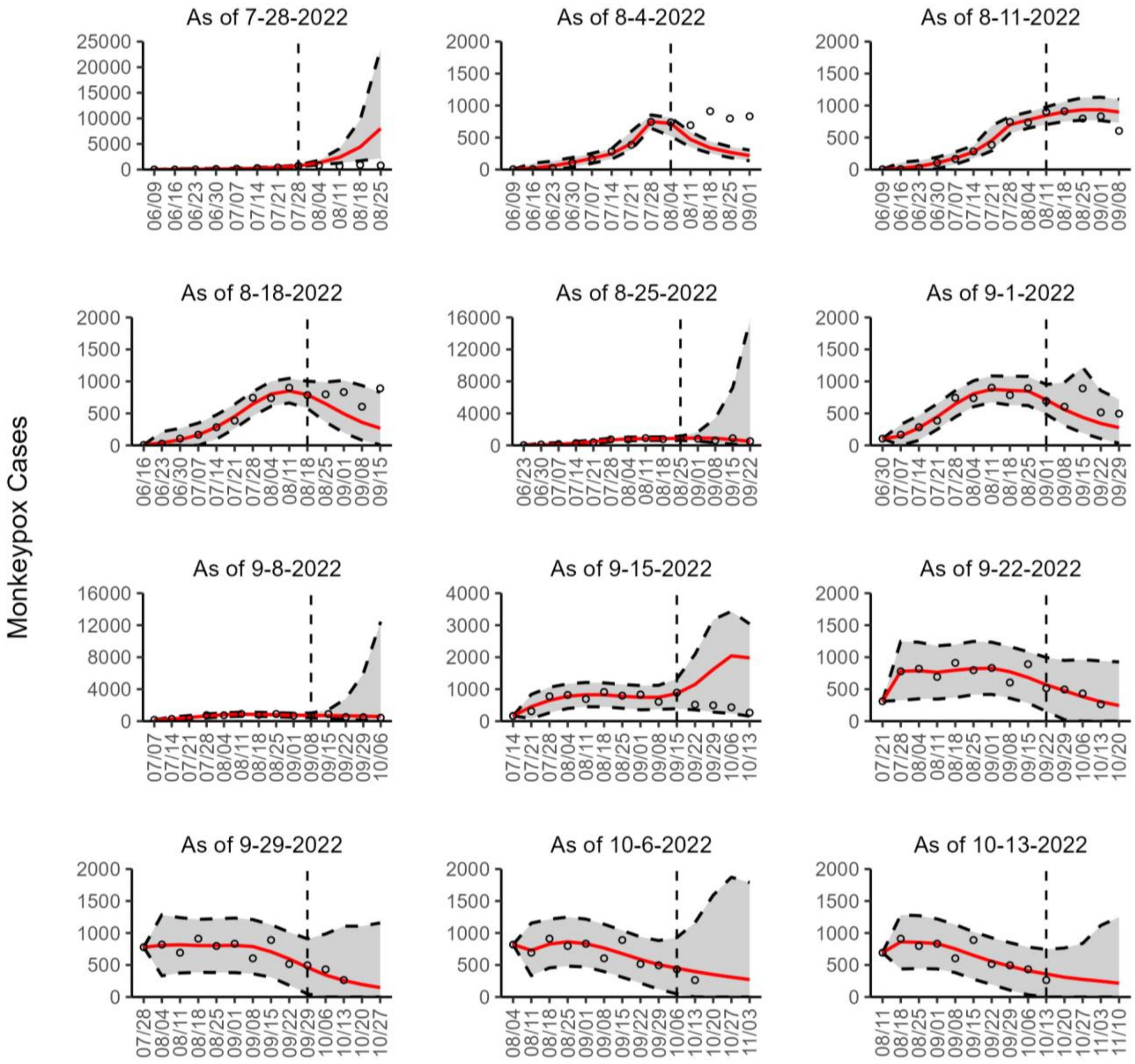

Figure 20s. The overlayed forecasted and reported monkeypox cases for the weeks of 7/28/2022 through the week of 10/13/2022 for Brazil. The forecasts are derived from the second-ranked sub-epidemic model using 10-week calibration data, and the reported cases are obtained from the OWID GitHub [25]. The black circles to the left of the vertical line represent the reported cases as of the Friday of the forecast period; the solid red line corresponds to the best fit model; the dashed black lines correspond to the 95% prediction intervals. The black circles to the right of the vertical line represent the reported case counts (as of 10/21/2022) for the corresponding date. The vertical dashed black line indicates the start of the forecast period. For the week of 7/28/2022, data posted by the OWID team on 8/9/2022 was used to produce the forecast as it was the earliest version of data available.

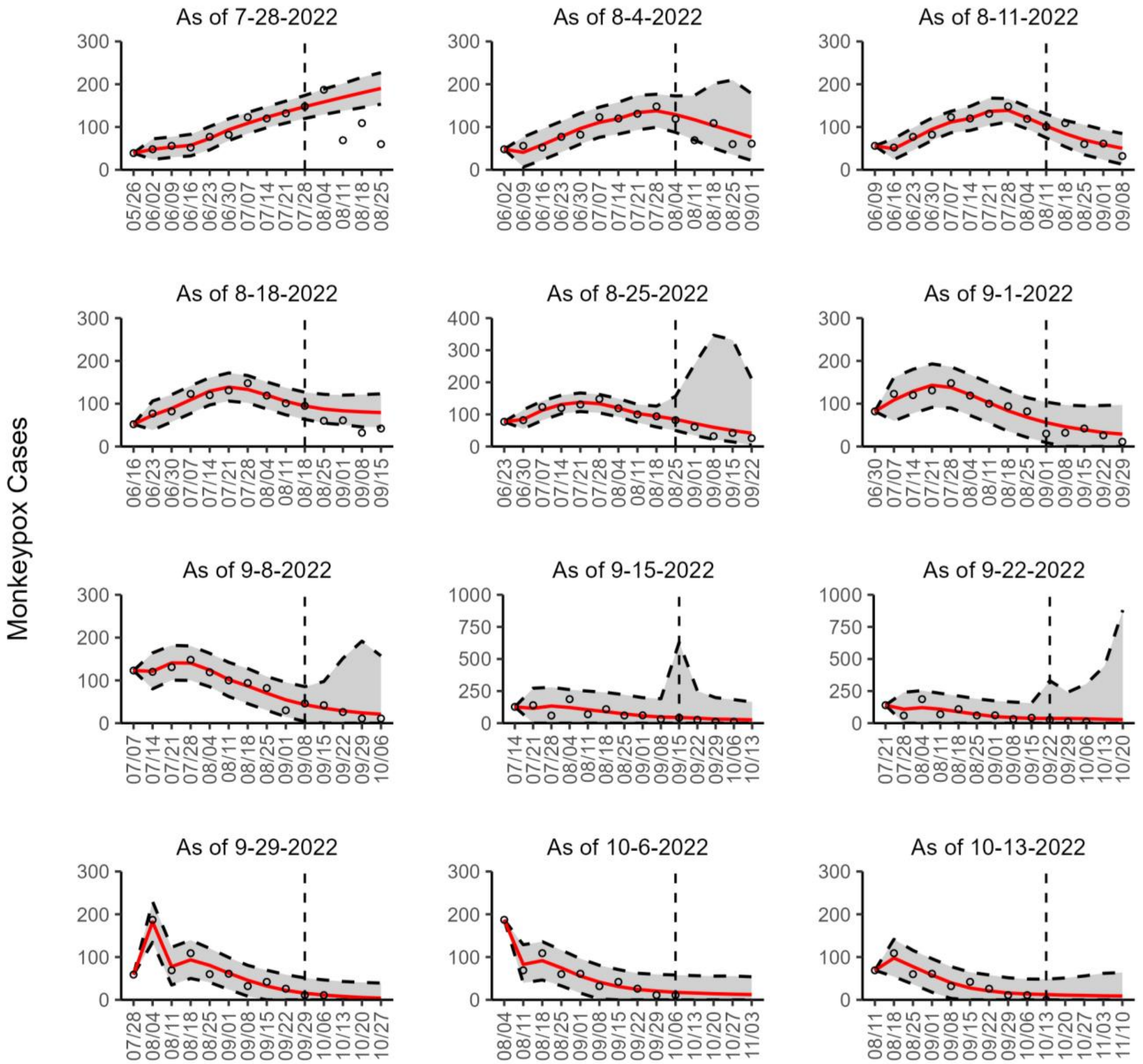

Figure 21s. The overlayed forecasted and reported monkeypox cases for the weeks of 7/28/2022 through the week of 10/13/2022 for Canada. The forecasts are derived from the second-ranked sub-epidemic model using 10-week calibration data, and the reported cases are obtained from the OWID GitHub [25]. The black circles to the left of the vertical line represent the reported cases as of the Friday of the forecast period; the solid red line corresponds to the best fit model; the dashed black lines correspond to the 95% prediction intervals. The black circles to the right of the vertical line represent the reported case counts (as of 10/21/2022) for the corresponding date. The vertical dashed black line indicates the start of the forecast period. For the week of 7/28/2022, data posted by the OWID team on 8/9/2022 was used to produce the forecast as it was the earliest version of data available.

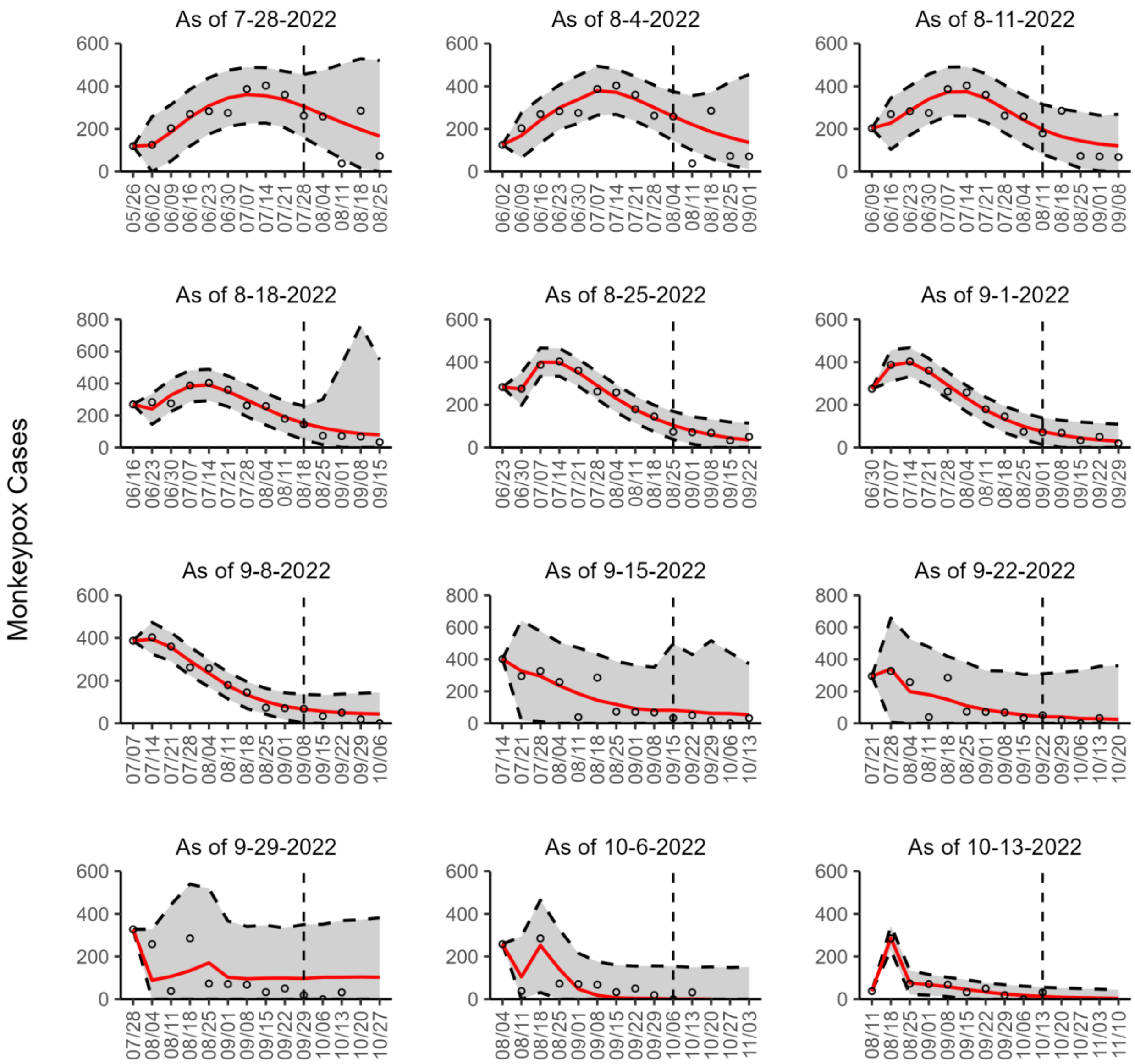

Figure 22s. The overlayed forecasted and reported monkeypox cases for the weeks of 7/28/2022 through the week of 10/13/2022 for England. The forecasts are derived from the second-ranked sub-epidemic model using 10-week calibration data, and the reported cases are obtained from the OWID GitHub [25]. The black circles to the left of the vertical line represent the reported cases as of the Friday of the forecast period; the solid red line corresponds to the best fit model; the dashed black lines correspond to the 95% prediction intervals. The black circles to the right of the vertical line represent the reported case counts (as of 10/21/2022) for the corresponding date. The vertical dashed black line indicates the start of the forecast period. For the week of 7/28/2022, data posted by the OWID team on 8/9/2022 was used to produce the forecast as it was the earliest version of data available.

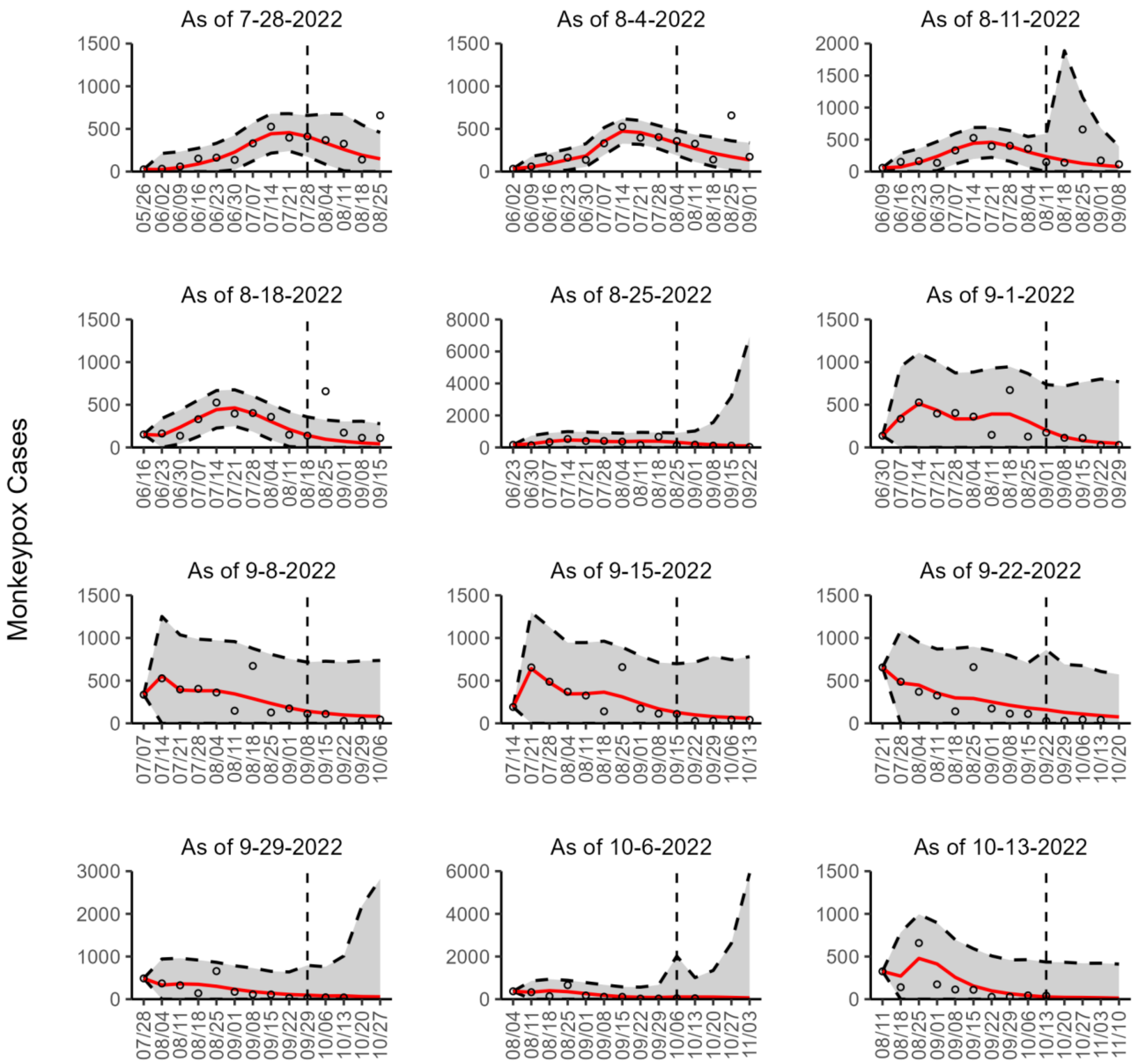

Figure 23s. The overlayed forecasted and reported monkeypox cases for the weeks of 7/28/2022 through the week of 10/13/2022 for France. The forecasts are derived from the second-ranked sub-epidemic model using 10-week calibration data, and the reported cases are obtained from the OWID GitHub [25]. The black circles to the left of the vertical line represent the reported cases as of the Friday of the forecast period; the solid red line corresponds to the best fit model; the dashed black lines correspond to the 95% prediction intervals. The black circles to the right of the vertical line represent the reported case counts (as of 10/21/2022) for the corresponding date. The vertical dashed black line indicates the start of the forecast period. For the week of 7/28/2022, data posted by the OWID team on 8/9/2022 was used to produce the forecast as it was the earliest version of data available.

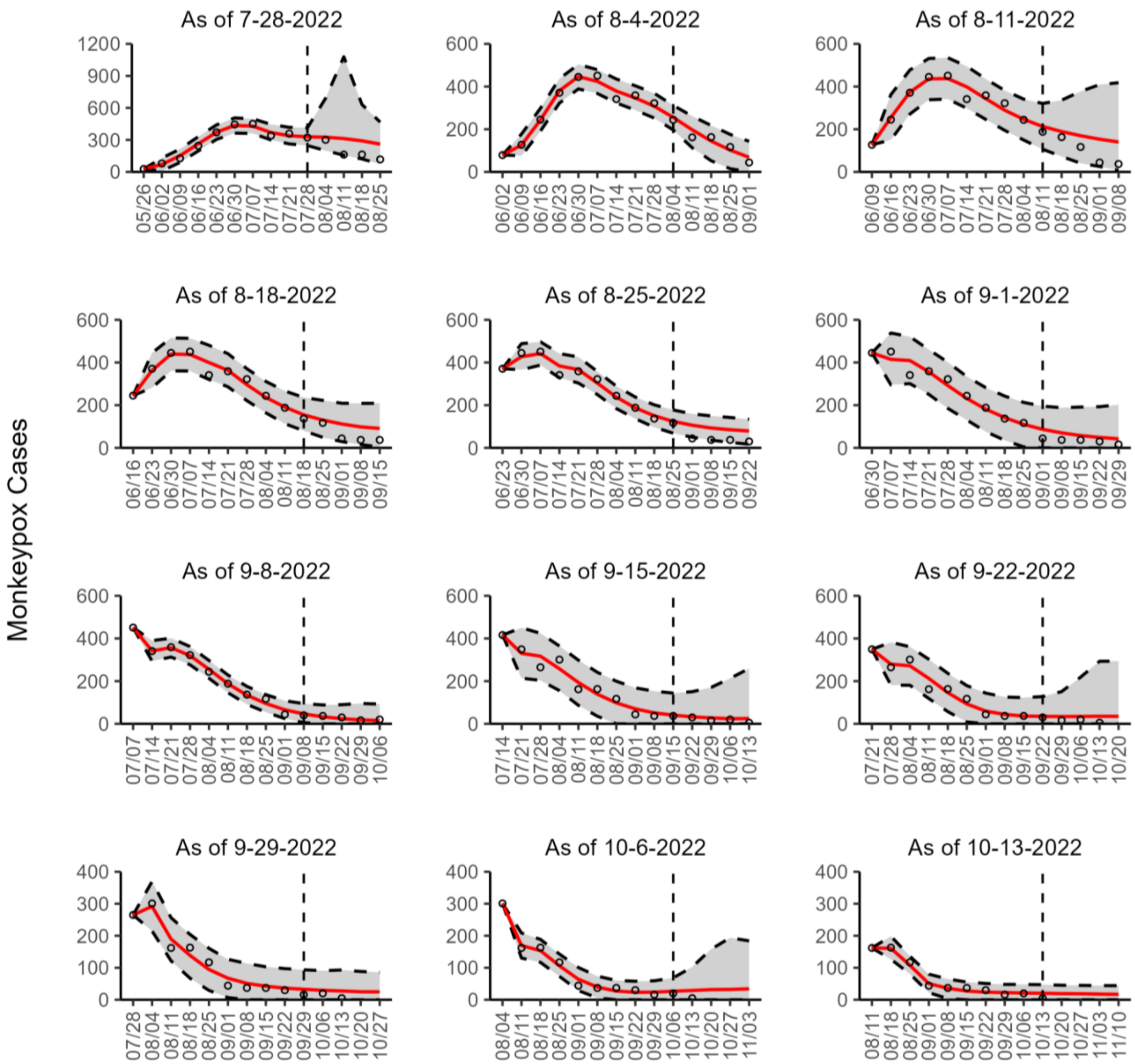

Figure 24s. The overlayed forecasted and reported monkeypox cases for the weeks of 7/28/2022 through the week of 10/13/2022 for Germany. The forecasts are derived from the second-ranked sub-epidemic model using 10-week calibration data, and the reported cases are obtained from the OWID GitHub [25]. The black circles to the left of the vertical line represent the reported cases as of the Friday of the forecast period; the solid red line corresponds to the best fit model; the dashed black lines correspond to the 95% prediction intervals. The black circles to the right of the vertical line represent the reported case counts (as of 10/21/2022) for the corresponding date. The vertical dashed black line indicates the start of the forecast period. For the week of 7/28/2022, data posted by the OWID team on 8/9/2022 was used to produce the forecast as it was the earliest version of data available.

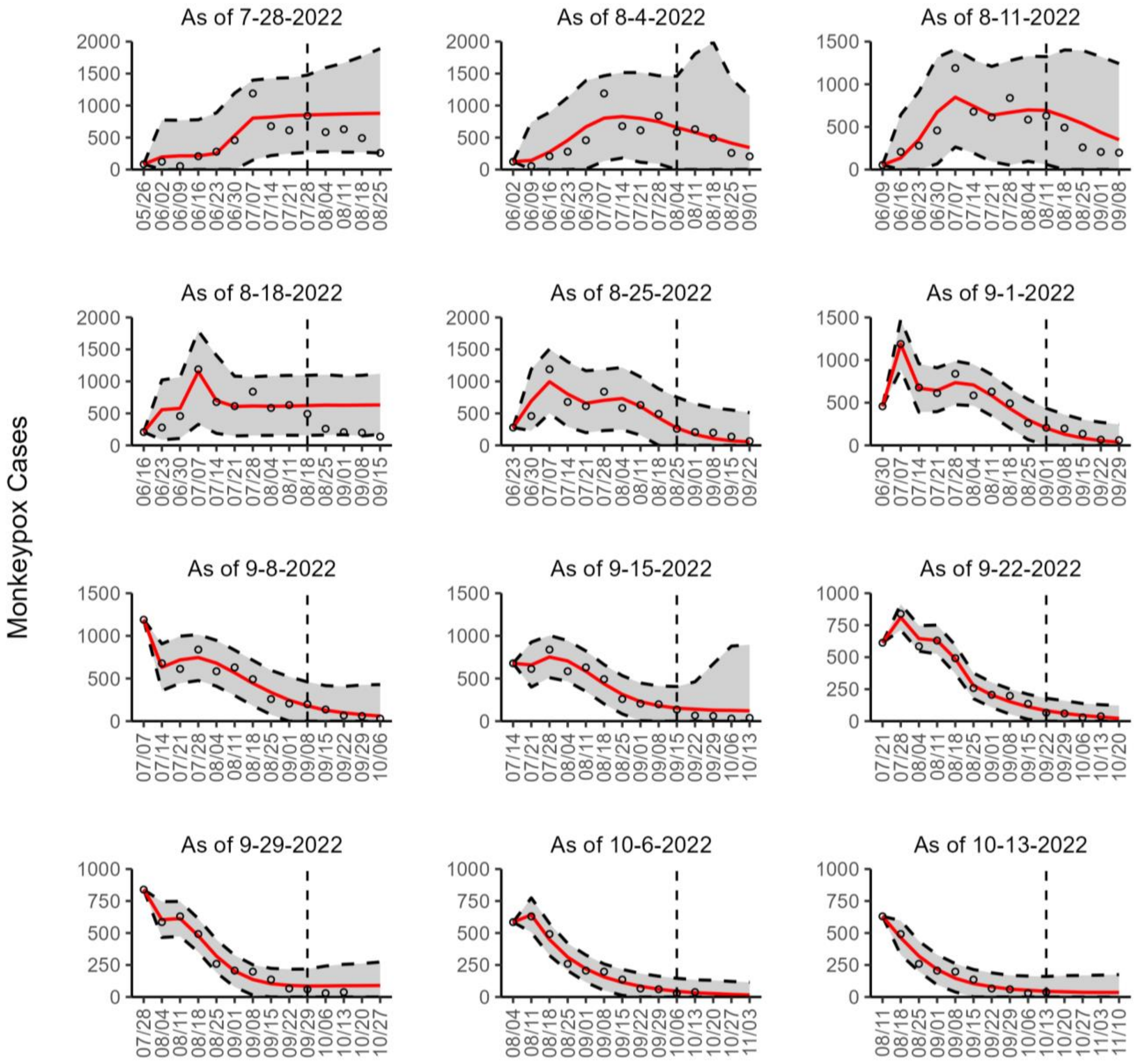

Figure 25s. The overlayed forecasted and reported monkeypox cases for the weeks of 7/28/2022 through the week of 10/13/2022 for Spain. The forecasts are derived from the second-ranked sub-epidemic model using 10-week calibration data, and the reported cases are obtained from the OWID GitHub [25]. The black circles to the left of the vertical line represent the reported cases as of the Friday of the forecast period; the solid red line corresponds to the best fit model; the dashed black lines correspond to the 95% prediction intervals. The black circles to the right of the vertical line represent the reported case counts (as of 10/21/2022) for the corresponding date. The vertical dashed black line indicates the start of the forecast period. For the week of 7/28/2022, data posted by the OWID team on 8/9/2022 was used to produce the forecast as it was the earliest version of data available.

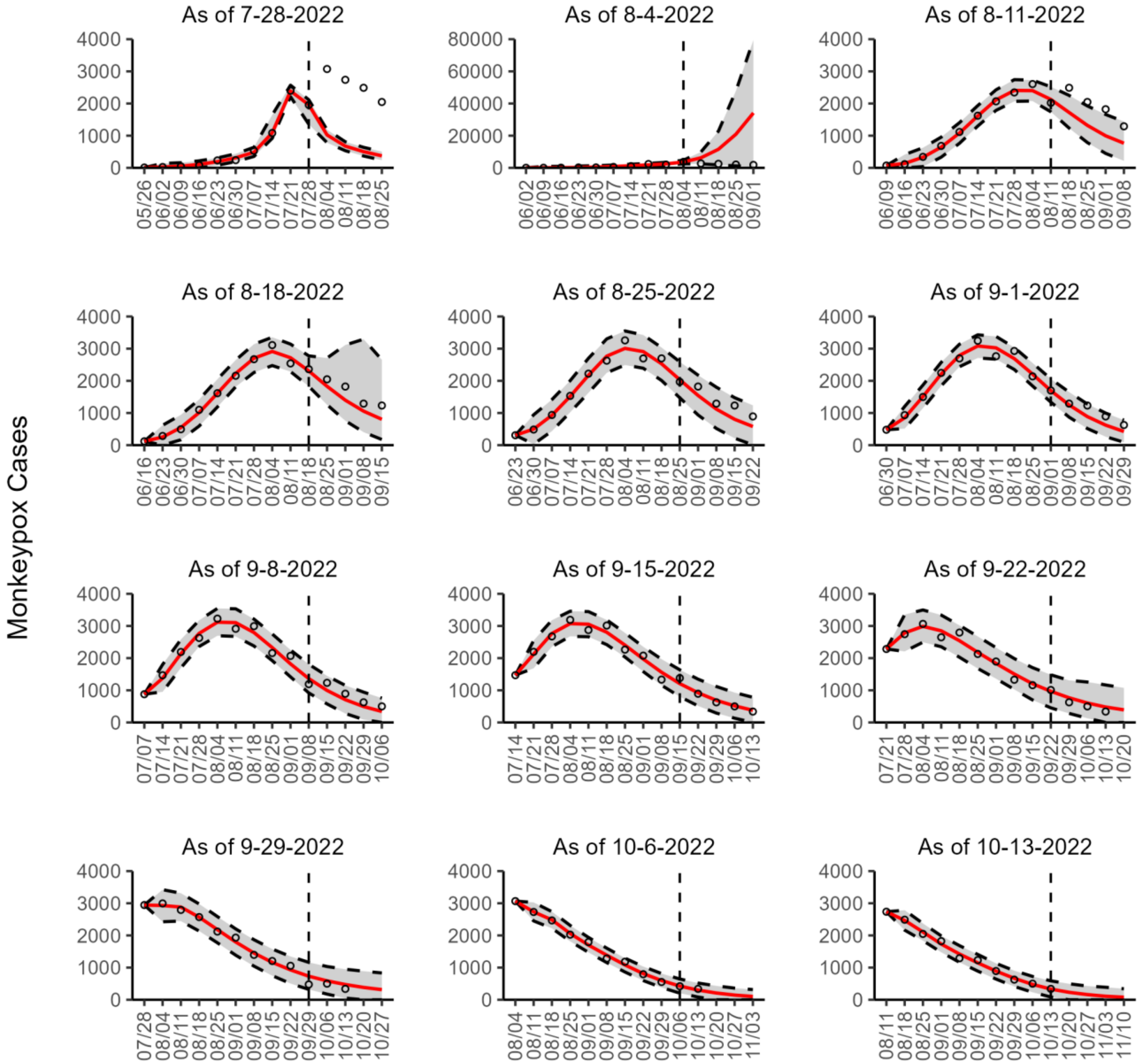

Figure 26s. The overlayed forecasted and reported monkeypox cases for the weeks of 7/28/2022 through the week of 10/13/2022 for the United States. The forecasts are derived from the second-ranked sub-epidemic model using 10-week calibration data, and the reported cases are obtained from the CDC [24]. The black circles to the left of the vertical line represent the reported cases as of the Wednesday of the forecast period; the solid red line corresponds to the best fit model; the dashed black lines correspond to the 95% prediction intervals. The black circles to the right of the vertical line represent the reported case counts (as of 10/19/2022) for the corresponding date.

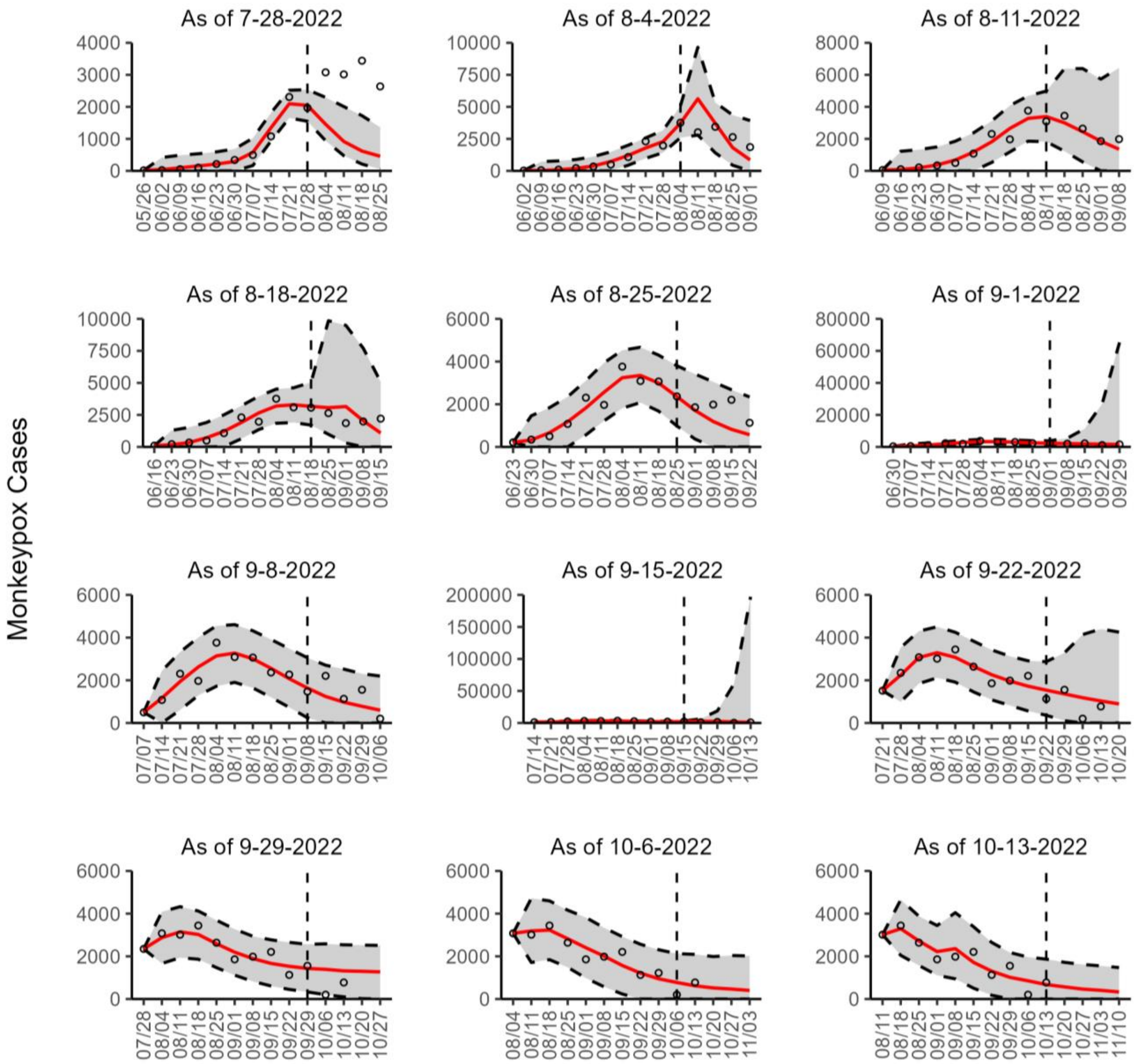

Figure 27s. The overlayed forecasted and reported monkeypox cases for the weeks of 7/28/2022 through the week of 10/13/2022 for the United States. The forecasts are derived from the second-ranked sub-epidemic model using 10-week calibration data, and the reported cases are obtained from the OWID GitHub [25]. The black circles to the left of the vertical line represent the reported cases as of the Friday of the forecast period; the solid red line corresponds to the best fit model; the dashed black lines correspond to the 95% prediction intervals. The black circles to the right of the vertical line represent the reported case counts (as of 10/21/2022) for the corresponding date. The vertical dashed black line indicates the start of the forecast period. For the week of 7/28/2022, data posted by the OWID team on 8/9/2022 was used to produce the forecast as it was the earliest version of data available.

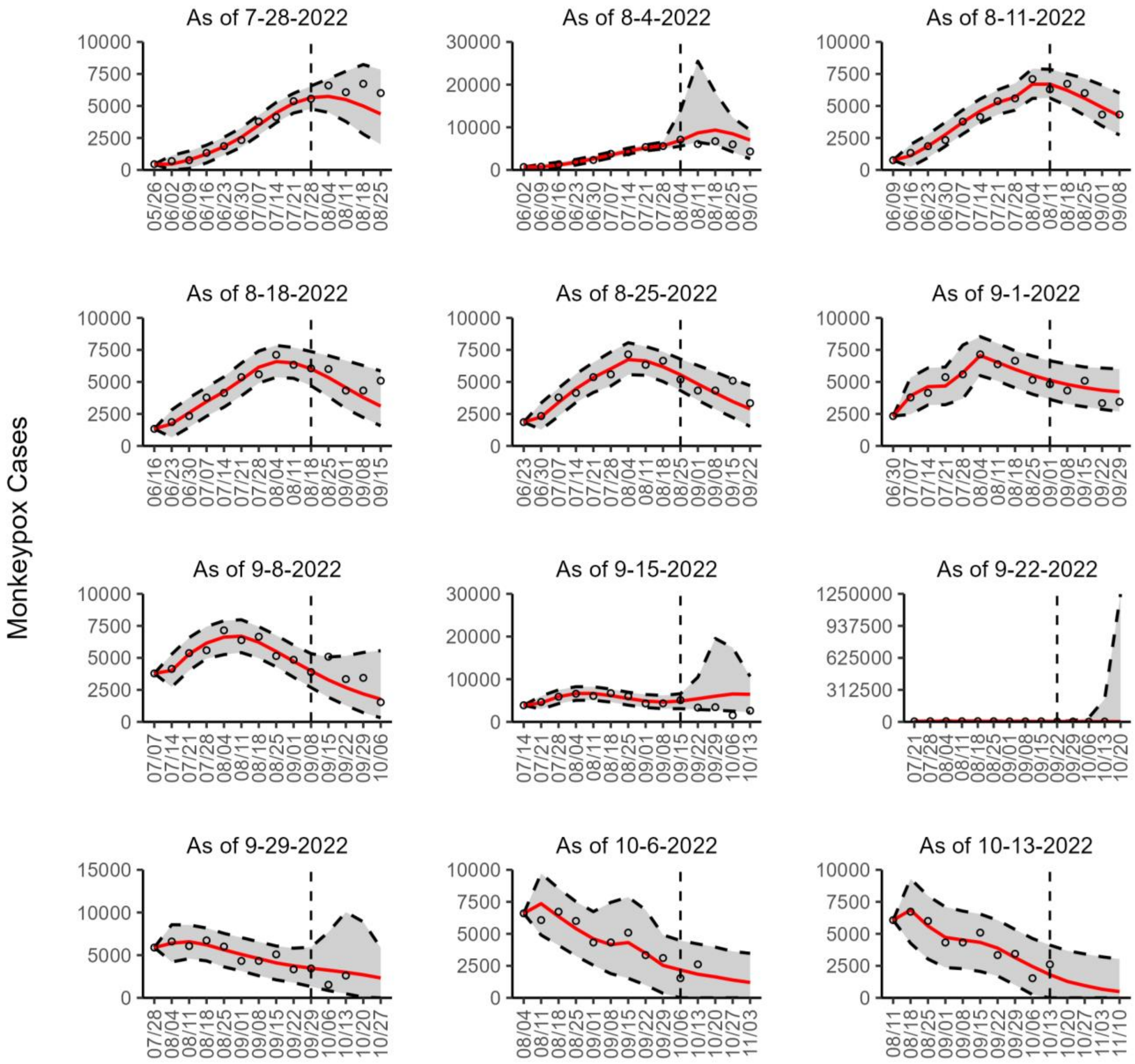

Figure 28s. The overlayed forecasted and reported monkeypox cases for the weeks of 7/28/2022 through the week of 10/13/2022 for the World. The forecasts are derived from the second-ranked sub-epidemic model using 10-week calibration data, and the reported cases are obtained from the OWID GitHub [25]. The black circles to the left of the vertical line represent the reported cases as of the Friday of the forecast period; the solid red line corresponds to the best fit model; the dashed black lines correspond to the 95% prediction intervals. The black circles to the right of the vertical line represent the reported case counts (as of 10/21/2022) for the corresponding date. The vertical dashed black line indicates the start of the forecast period. For the week of 7/28/2022, data posted by the OWID team on 8/9/2022 was used to produce the forecast as it was the earliest version of data available.

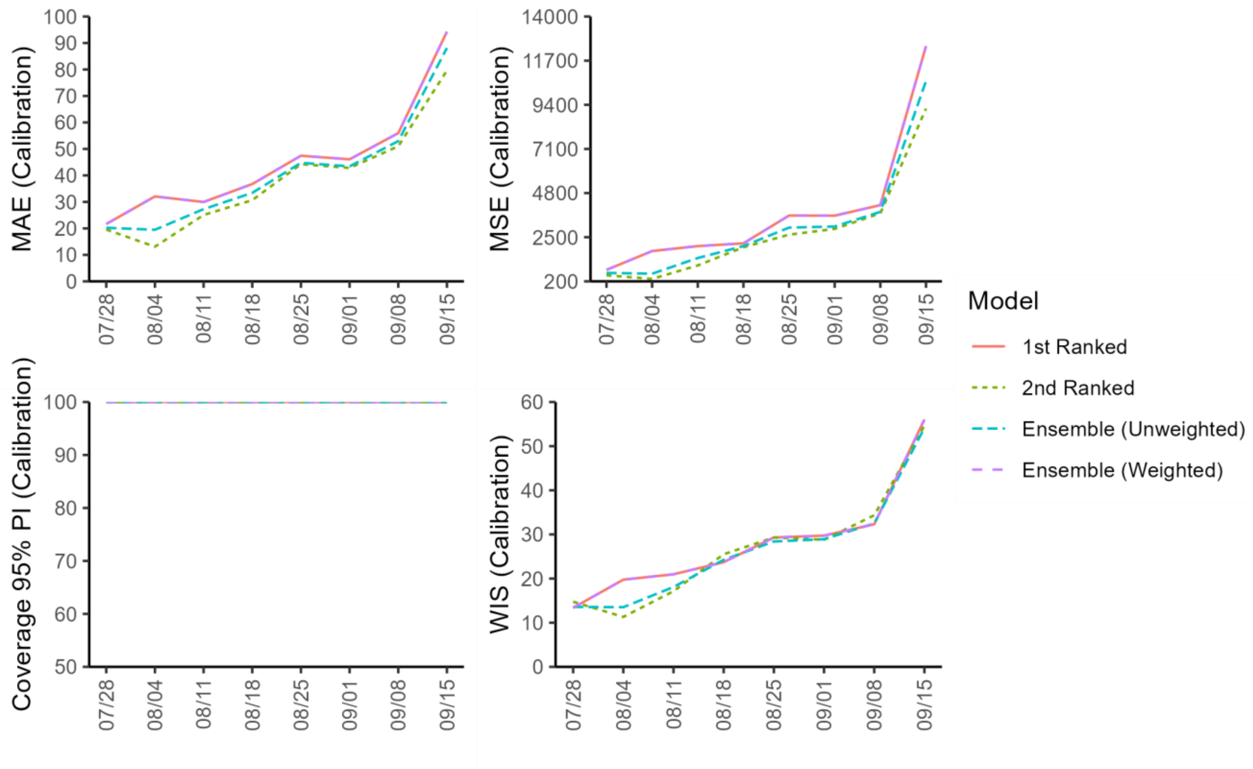

Figure 29s. Mean performance metrics for Brazil quantifying model fit across 20 sequential 10-week calibration periods (Week of May 26th – October 13th, 2022) using weekly timeseries data from the OWID team over each 8 forecasting periods**.** Only forecast periods in which observed data was available are included.

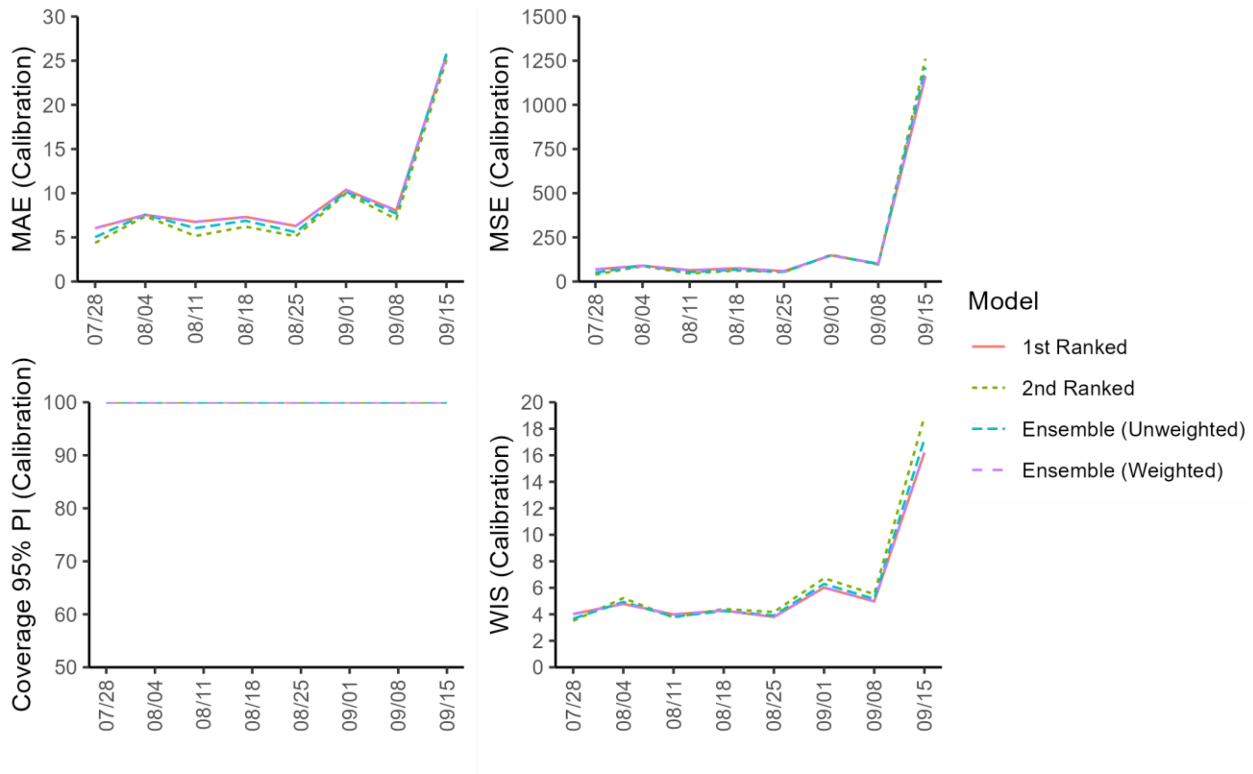

Figure 30s. Mean performance metrics for Canada quantifying model fit across 20 sequential 10-week calibration periods (Week of May 26th – October 13th, 2022) using weekly timeseries data from the OWID team over each 8 forecasting periods**.** Only forecast periods in which observed data was available are included.

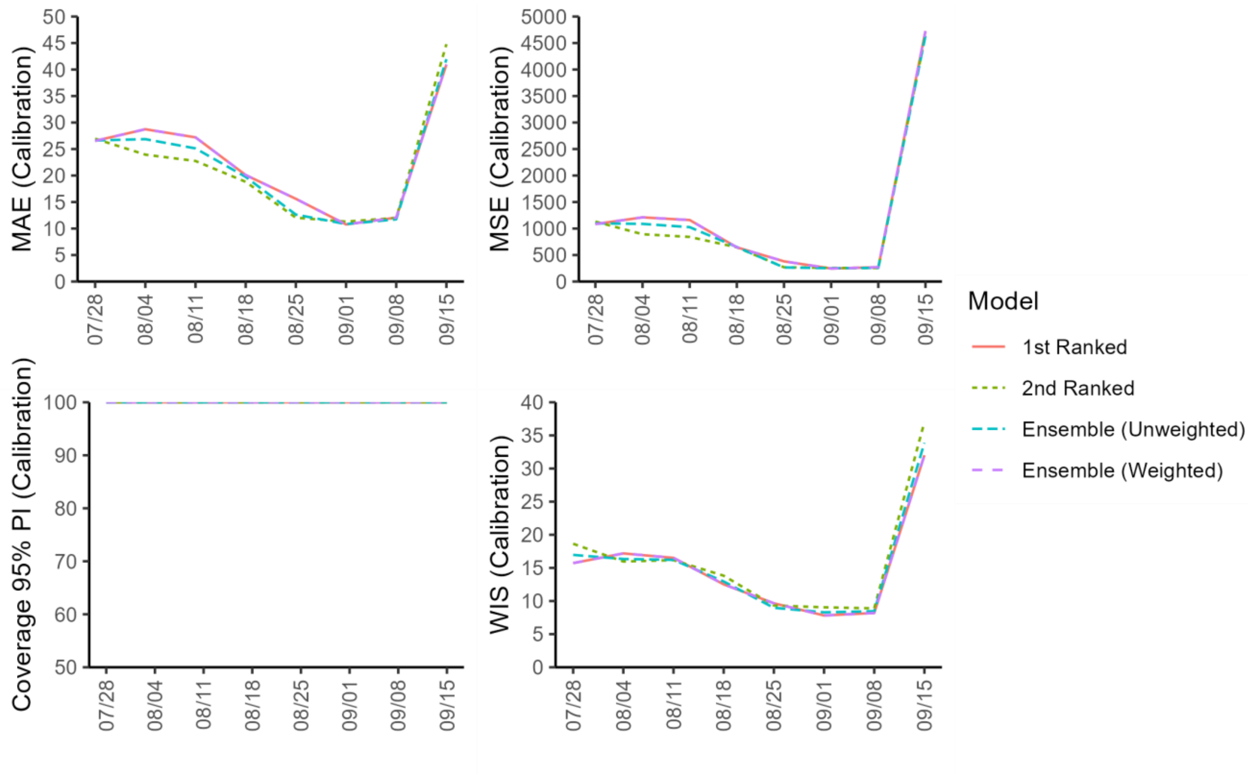

Figure 31s. Mean performance metrics for England quantifying model fit across 20 sequential 10-week calibration periods (Week of May 26th – October 13th, 2022) using weekly timeseries data from the OWID team over each 8 forecasting periods**.** Only forecast periods in which observed data was available are included.

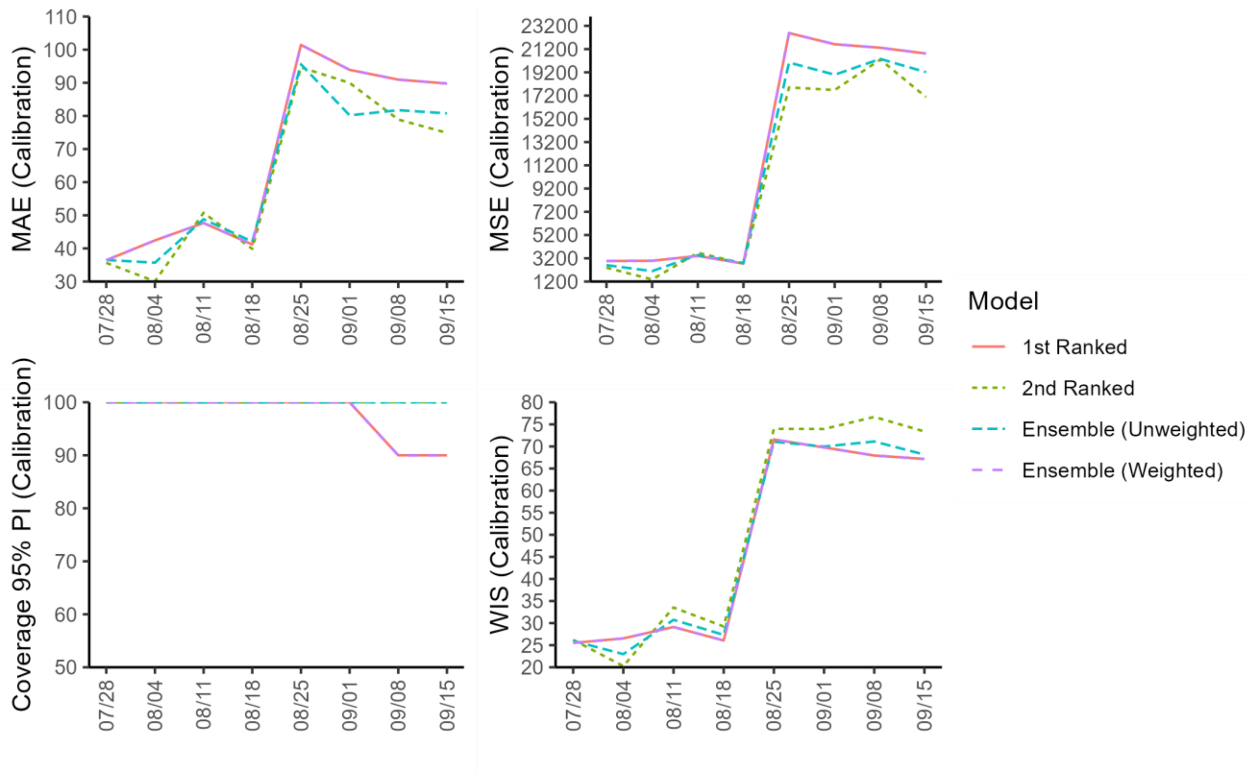

Figure 32s. Mean performance metrics for France quantifying model fit across 20 sequential 10-week calibration periods (Week of May 26th – October 13th, 2022) using weekly timeseries data from the OWID team over each 8 forecasting periods**.** Only forecast periods in which observed data was available are included.

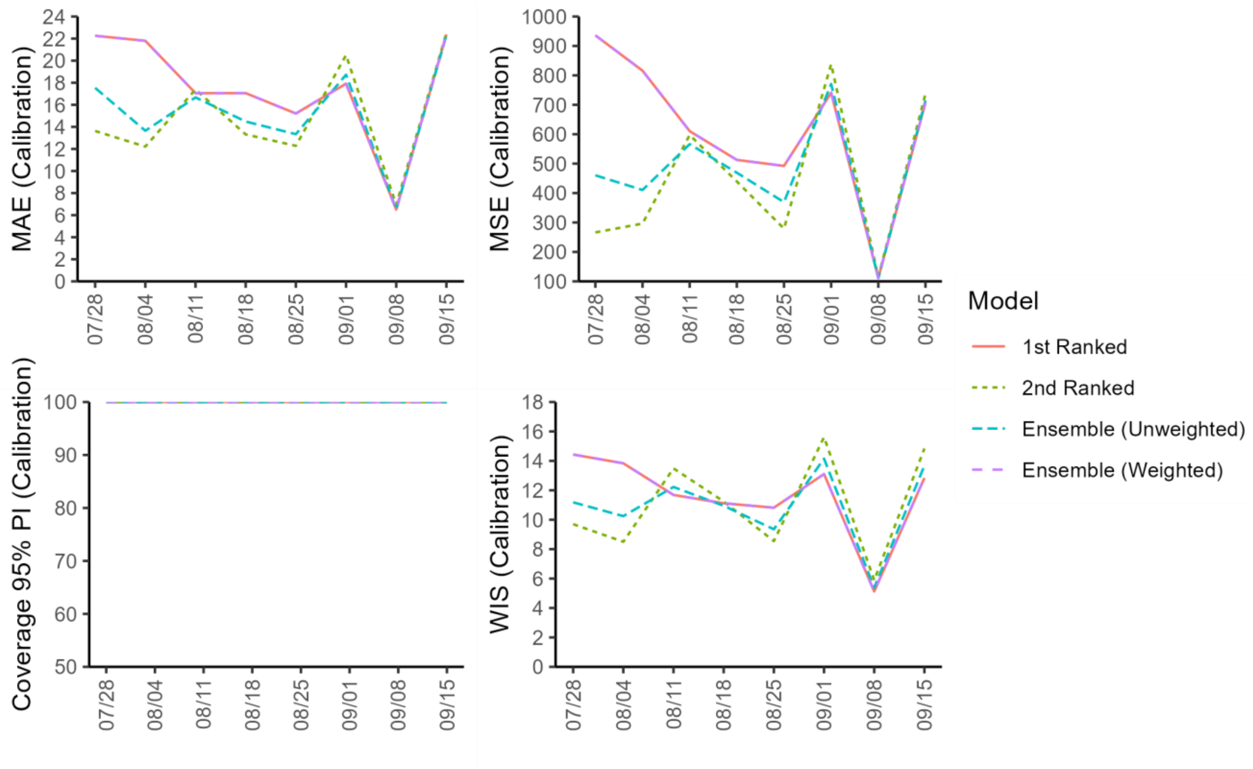

Figure 33s. Mean performance metrics for Germany quantifying model fit across 20 sequential 10-week calibration periods (Week of May 26th – October 13th, 2022) using weekly timeseries data from the OWID team over each 8 forecasting periods**.** Only forecast periods in which observed data was available are included.

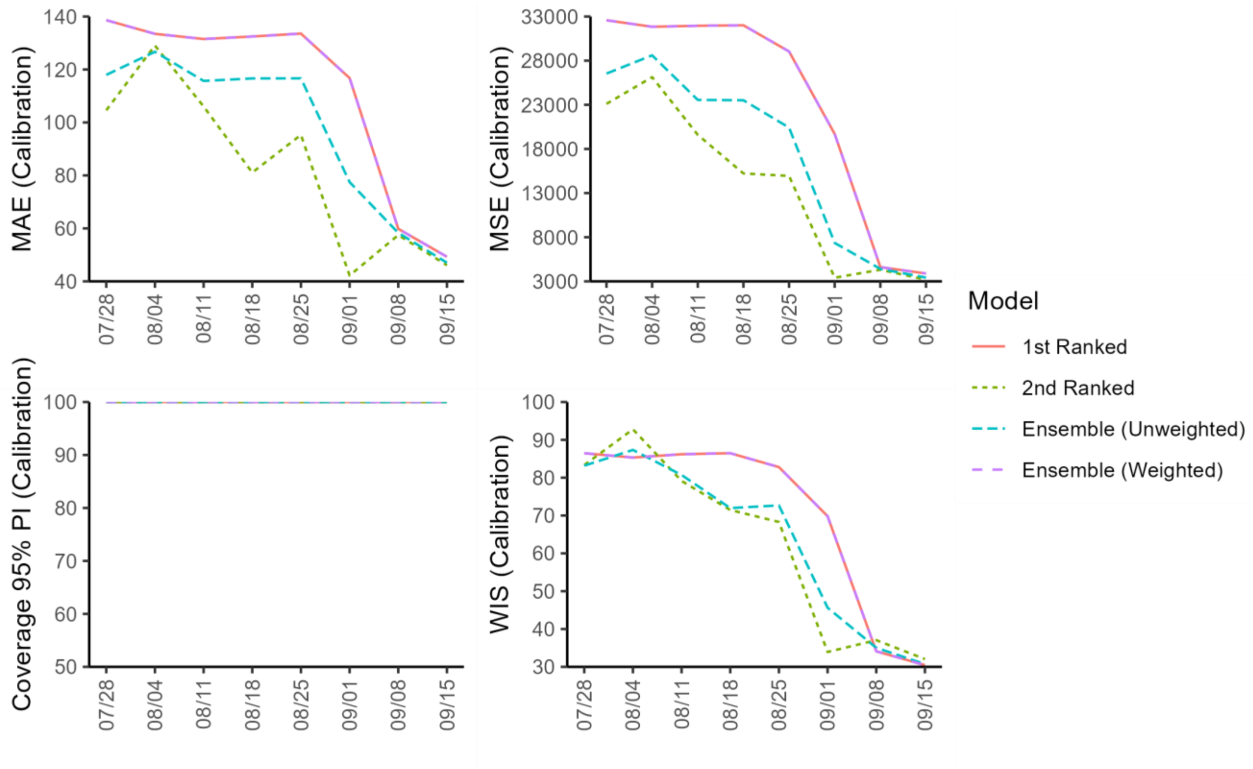

Figure 34s. Mean performance metrics for Spain quantifying model fit across 20 sequential 10-week calibration periods (Week of May 26th – October 13th, 2022) using weekly timeseries data from the OWID team over each 8 forecasting periods**.** Only forecast periods in which observed data was available are included.

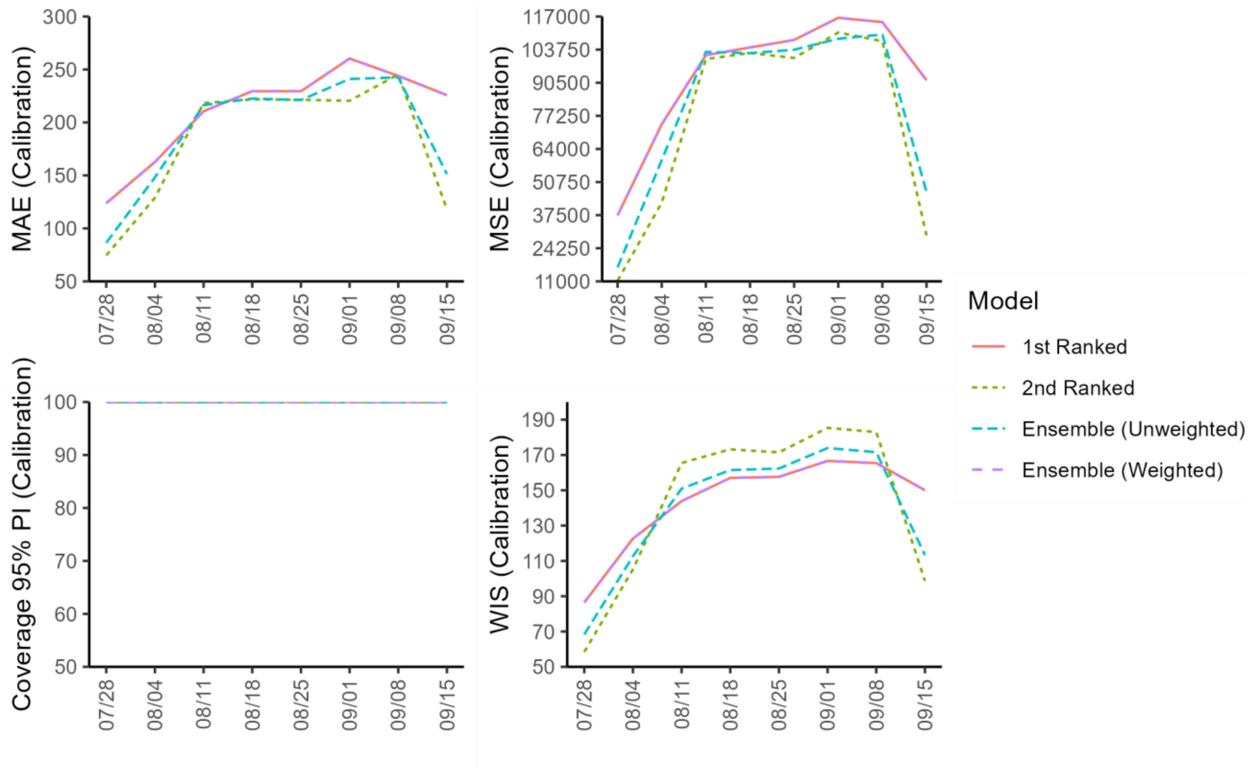

Figure 35s. Mean performance metrics for the United States quantifying model fit across 20 sequential 10-week calibration periods (Week of May 26th – October 13th, 2022) using weekly timeseries data from the OWID team over each 8 forecasting periods**.** Only forecast periods in which observed data was available are included.

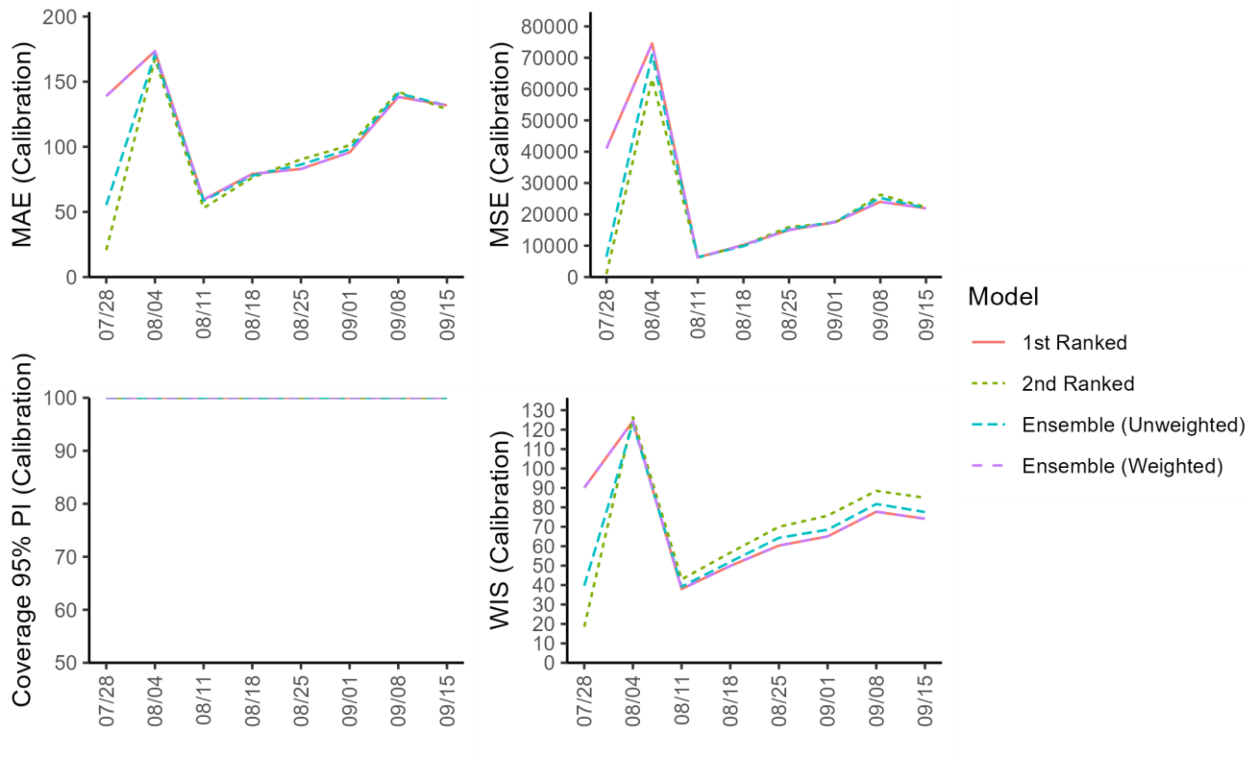

Figure 36s. Mean performance metrics for the United States quantifying model fit across 20 sequential 10-week calibration periods (Week of May 26th – October 13th, 2022) using weekly timeseries data from the CDC team over each 8 forecasting periods**.** Only forecast periods in which observed data was available are included.

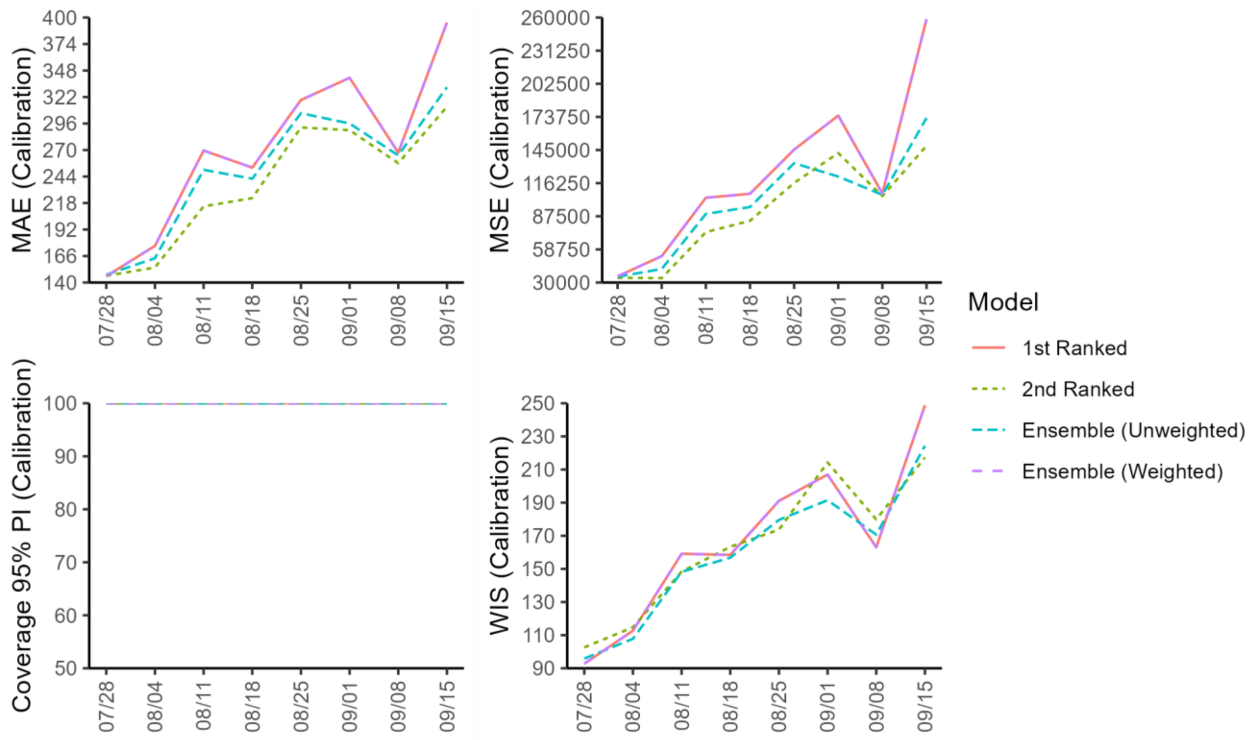

Figure 37s. Mean performance metrics for the World quantifying model fit across 20 sequential 10-week calibration periods (Week of May 26th – October 13th, 2022) using weekly timeseries data from the OWID team over each 8 forecasting periods**.** Only forecast periods in which observed data was available are included.

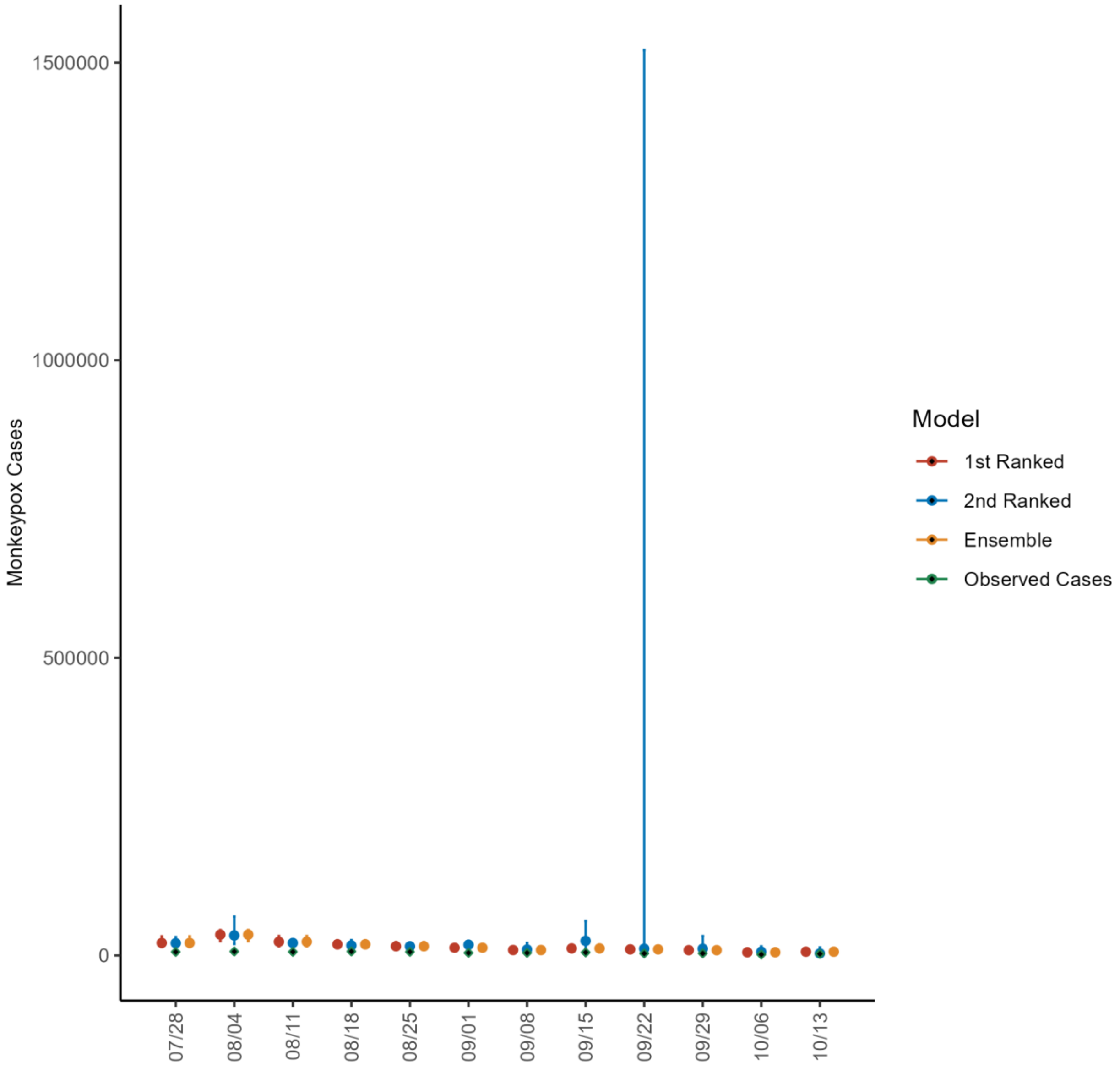

Figure 38s. Predicted cumulative number of new monkeypox cases for each of the 4-week ahead forecasts from the weeks of 7/28/2022 through 10/13/2022 based on a 10-week calibration period for the World. The solid circles indicate the estimated cumulative median value, and the bars indicate the corresponding 95% prediction interval. The observed cases are as reported to the OWID team as of 10/21/2022 [25]. For the week of 7/28/2022, data posted by the OWID team on 8/9/2022 was used to produce the forecast as it was the earliest version of data available.

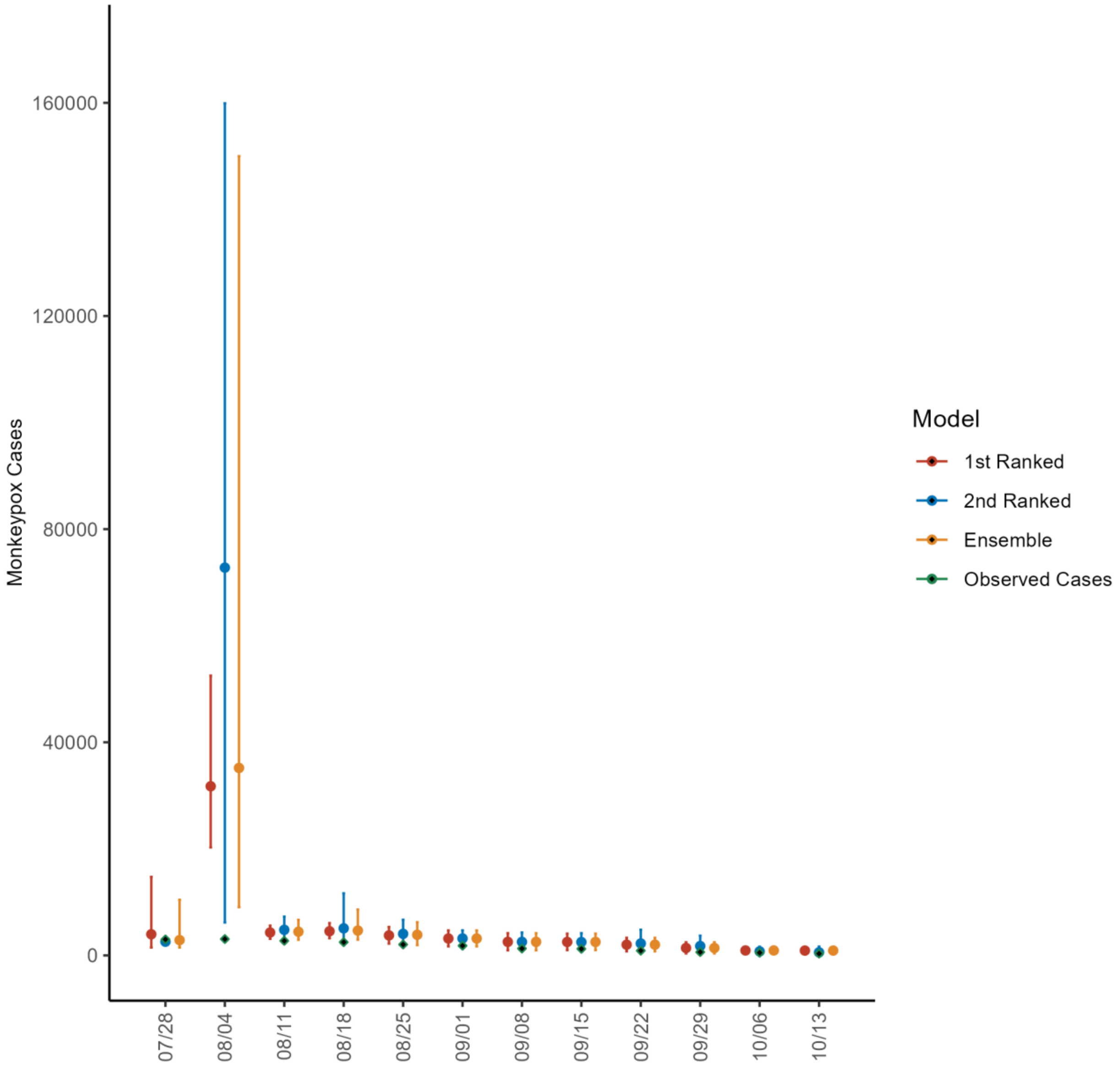

Figure 39s. Predicted cumulative number of new monkeypox cases for each of the 4-week ahead forecasts from the weeks of 7/28/2022 through 10/13/2022 based on a 10-week calibration period for the United States. The solid circles indicate the estimated cumulative median value, and the bars indicate the corresponding 95% prediction interval. The observed cases are as reported to the CDC as of 10/19/2022 [24].

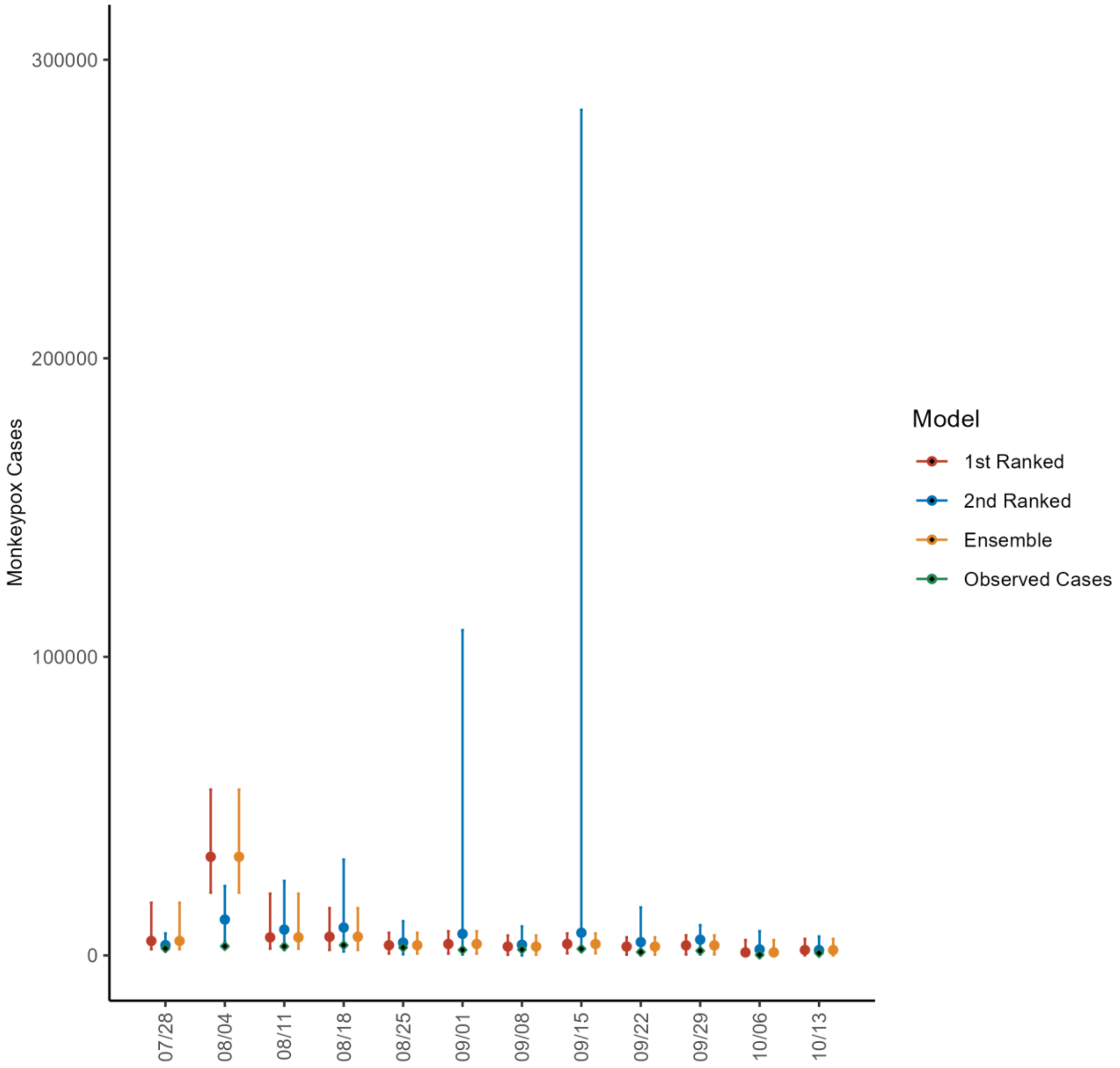

Figure 40s. Predicted cumulative number of new monkeypox cases for each of the 4-week ahead forecasts from the weeks of 7/28/2022 through 10/13/2022 based on a 10-week calibration period for the United States. The solid circles indicate the estimated cumulative median value, and the bars indicate the corresponding 95% prediction interval. The observed cases are as reported to the OWID team as of 10/21/2022 [25]. For the week of 7/28/2022, data posted by the OWID team on 8/9/2022 was used to produce the forecast as it was the earliest version of data available.

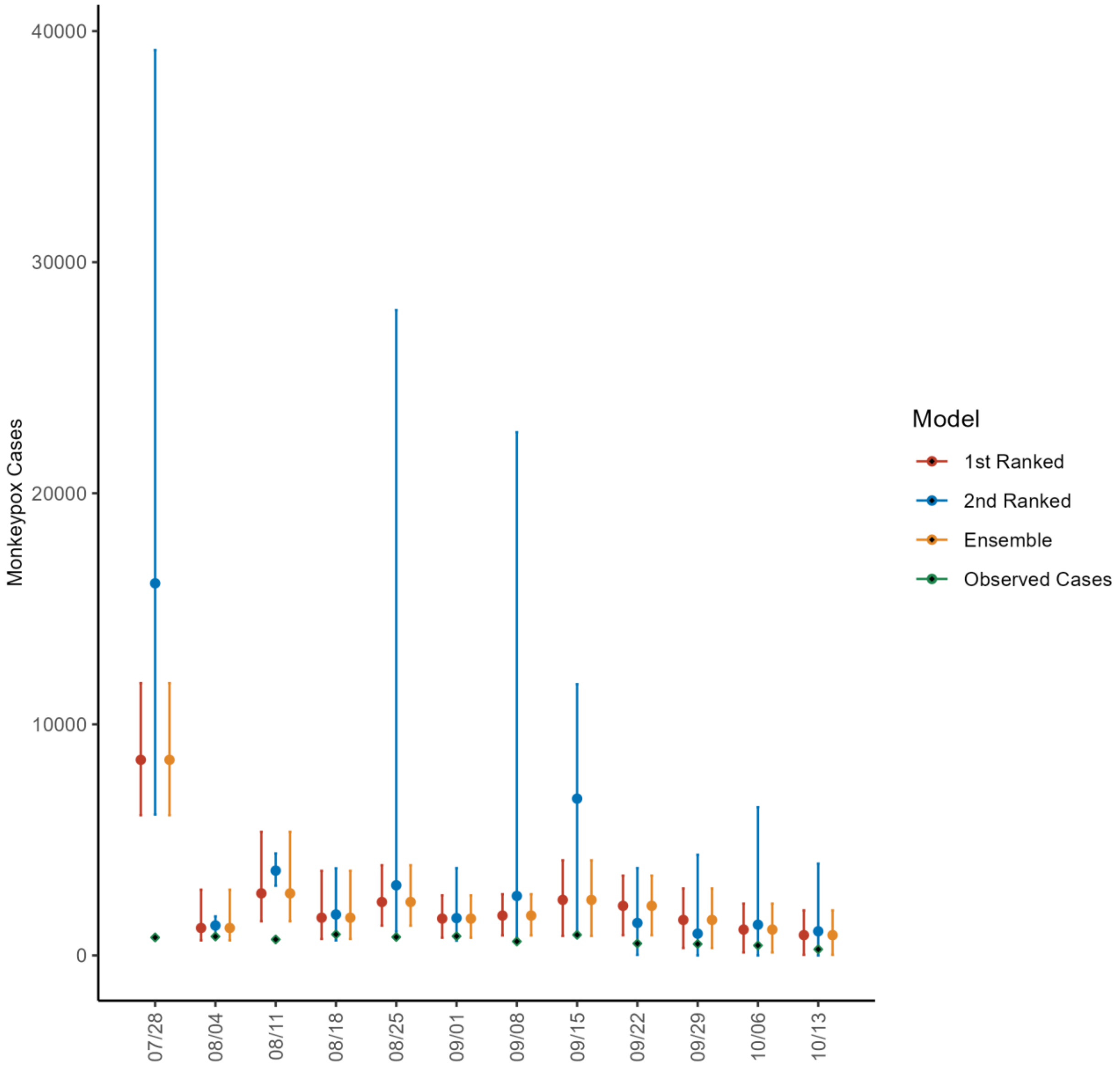

Figure 41s. Predicted cumulative number of new monkeypox cases for each of the 4-week ahead forecasts from the weeks of 7/28/2022 through 10/13/2022 based on a 10-week calibration period for Brazil. The solid circles indicate the estimated cumulative median value, and the bars indicate the corresponding 95% prediction interval. The observed cases are as reported to the OWID team as of 10/21/2022 [25]. For the week of 7/28/2022, data posted by the OWID team on 8/9/2022 was used to produce the forecast as it was the earliest version of data available.

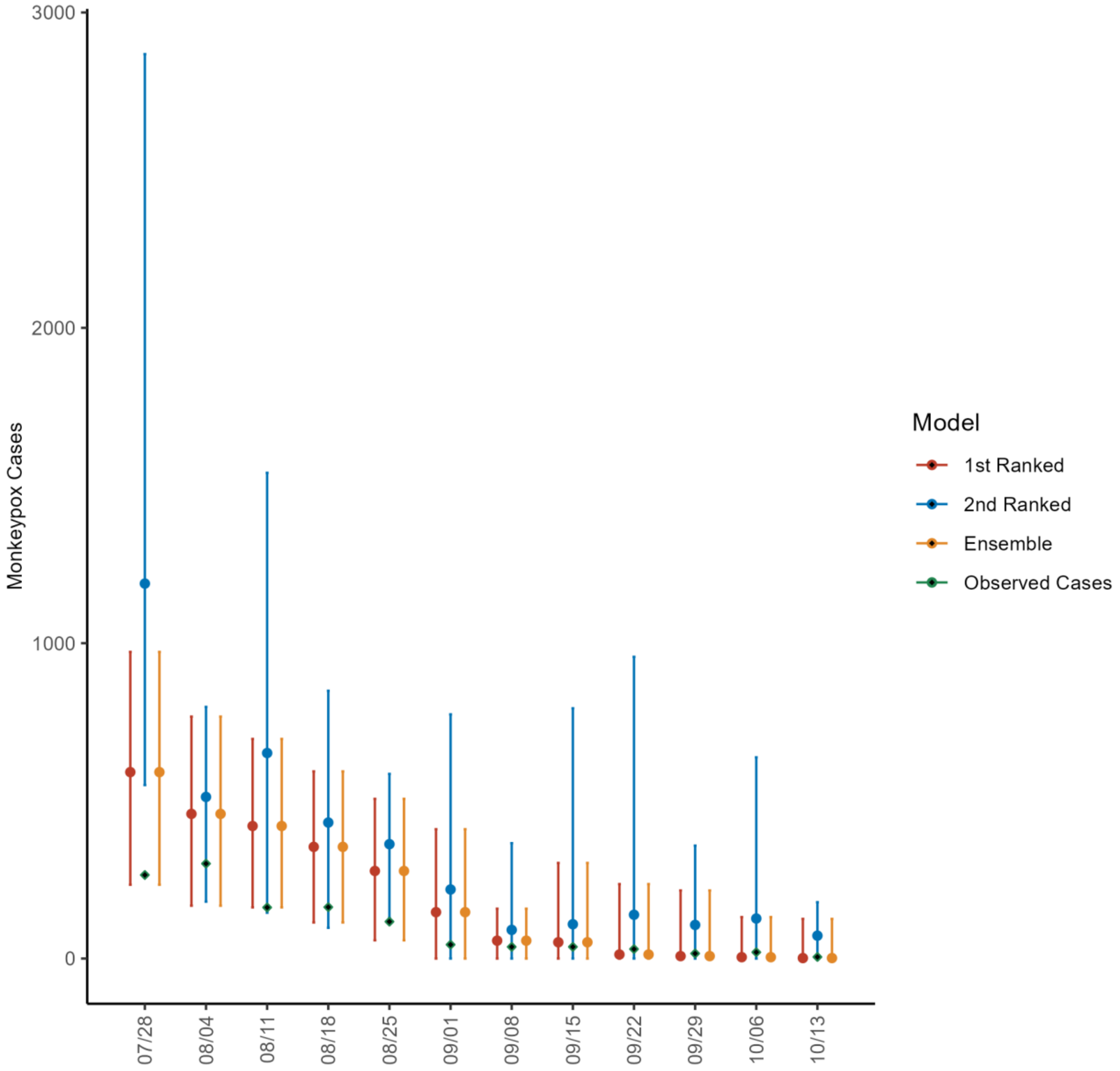

Figure 42s. Predicted cumulative number of new monkeypox cases for each of the 4-week ahead forecasts from the weeks of 7/28/2022 through 10/13/2022 based on a 10-week calibration period for Germany. The solid circles indicate the estimated cumulative median value, and the bars indicate the corresponding 95% prediction interval. The observed cases are as reported to the OWID team as of 10/21/2022 [25]. For the week of 7/28/2022, data posted by the OWID team on 8/9/2022 was used to produce the forecast as it was the earliest version of data available.

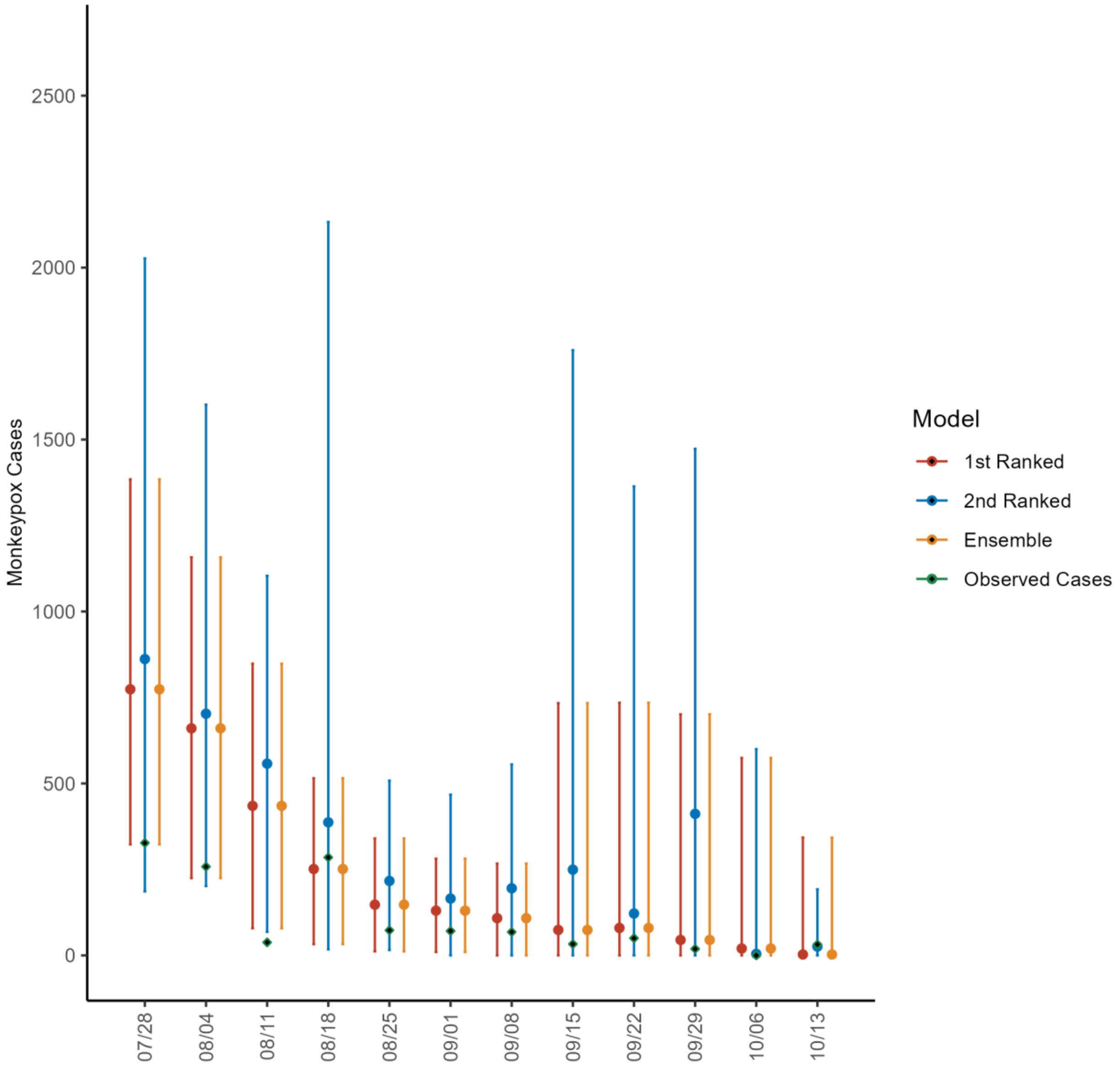

Figure 43s. Predicted cumulative number of new monkeypox cases for each of the 4-week ahead forecasts from the weeks of 7/28/2022 through 10/13/2022 based on a 10-week calibration period for England. The solid circles indicate the estimated cumulative median value, and the bars indicate the corresponding 95% prediction interval. The observed cases are as reported to the OWID team as of 10/21/2022 [25]. For the week of 7/28/2022, data posted by the OWID team on 8/9/2022 was used to produce the forecast as it was the earliest version of data available.

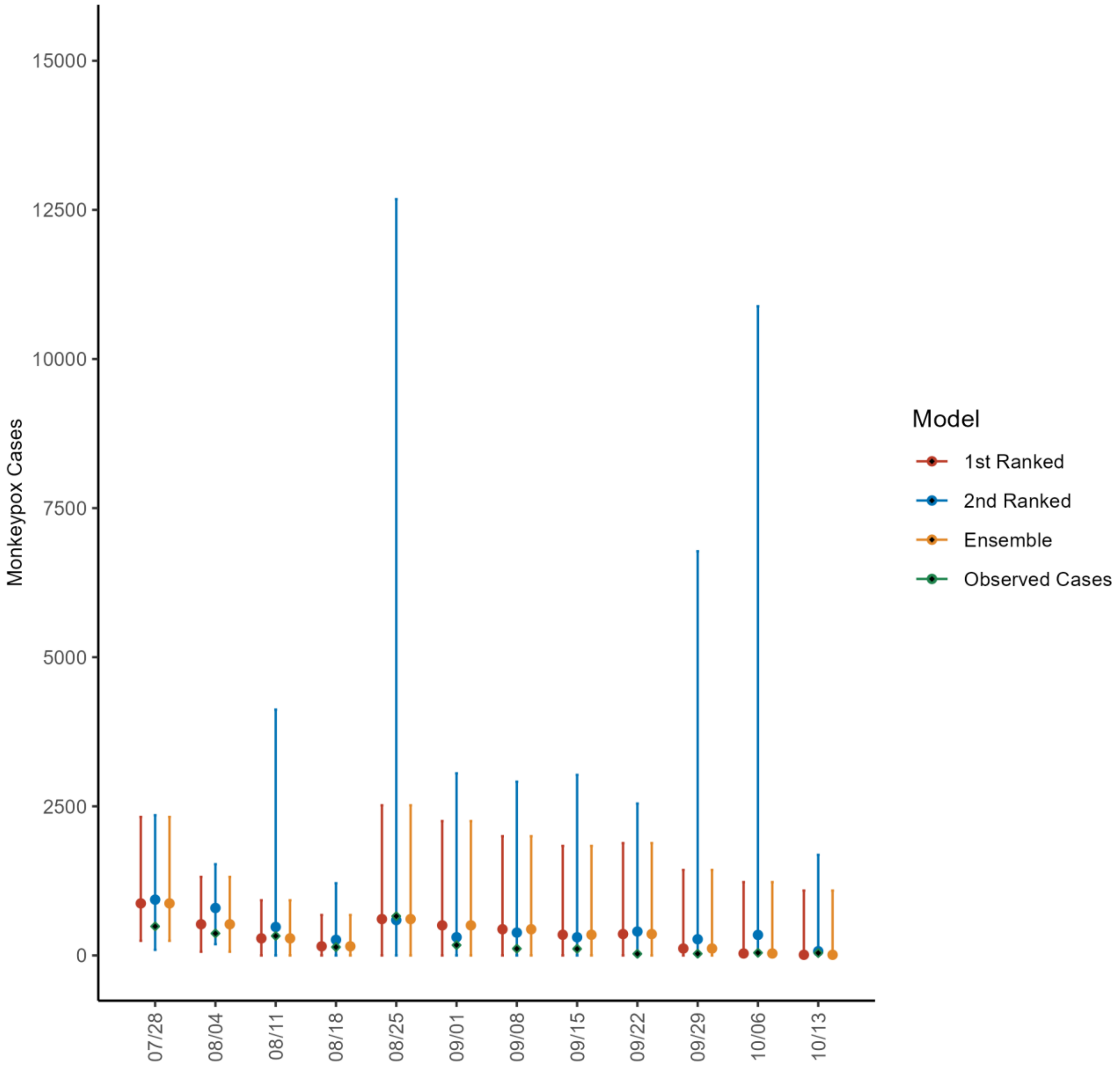

Figure 44s. Predicted cumulative number of new monkeypox cases for each of the 4-week ahead forecasts from the weeks of 7/28/2022 through 10/13/2022 based on a 10-week calibration period for France. The solid circles indicate the estimated cumulative median value, and the bars indicate the corresponding 95% prediction interval. The observed cases are as reported to the OWID team as of 10/21/2022 [25]. For the week of 7/28/2022, data posted by the OWID team on 8/9/2022 was used to produce the forecast as it was the earliest version of data available.

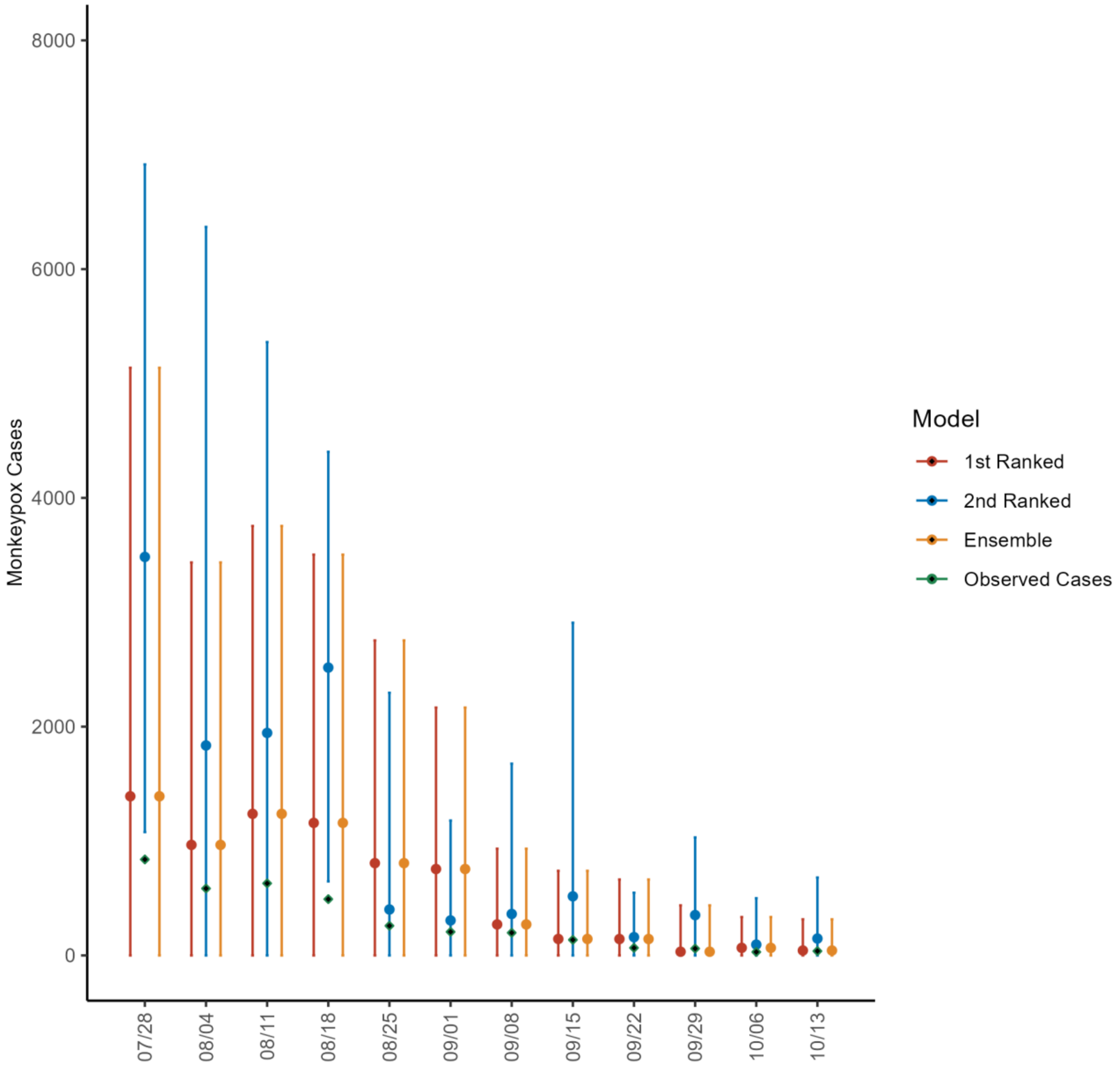

Figure 45s. Predicted cumulative number of new monkeypox cases for each of the 4-week ahead forecasts from the weeks of 7/28/2022 through 10/13/2022 based on a 10-week calibration period for Spain. The solid circles indicate the estimated cumulative median value, and the bars indicate the corresponding 95% prediction interval. The observed cases are as reported to the OWID team as of 10/21/2022 [25]. For the week of 7/28/2022, data posted by the OWID team on 8/9/2022 was used to produce the forecast as it was the earliest version of data available.

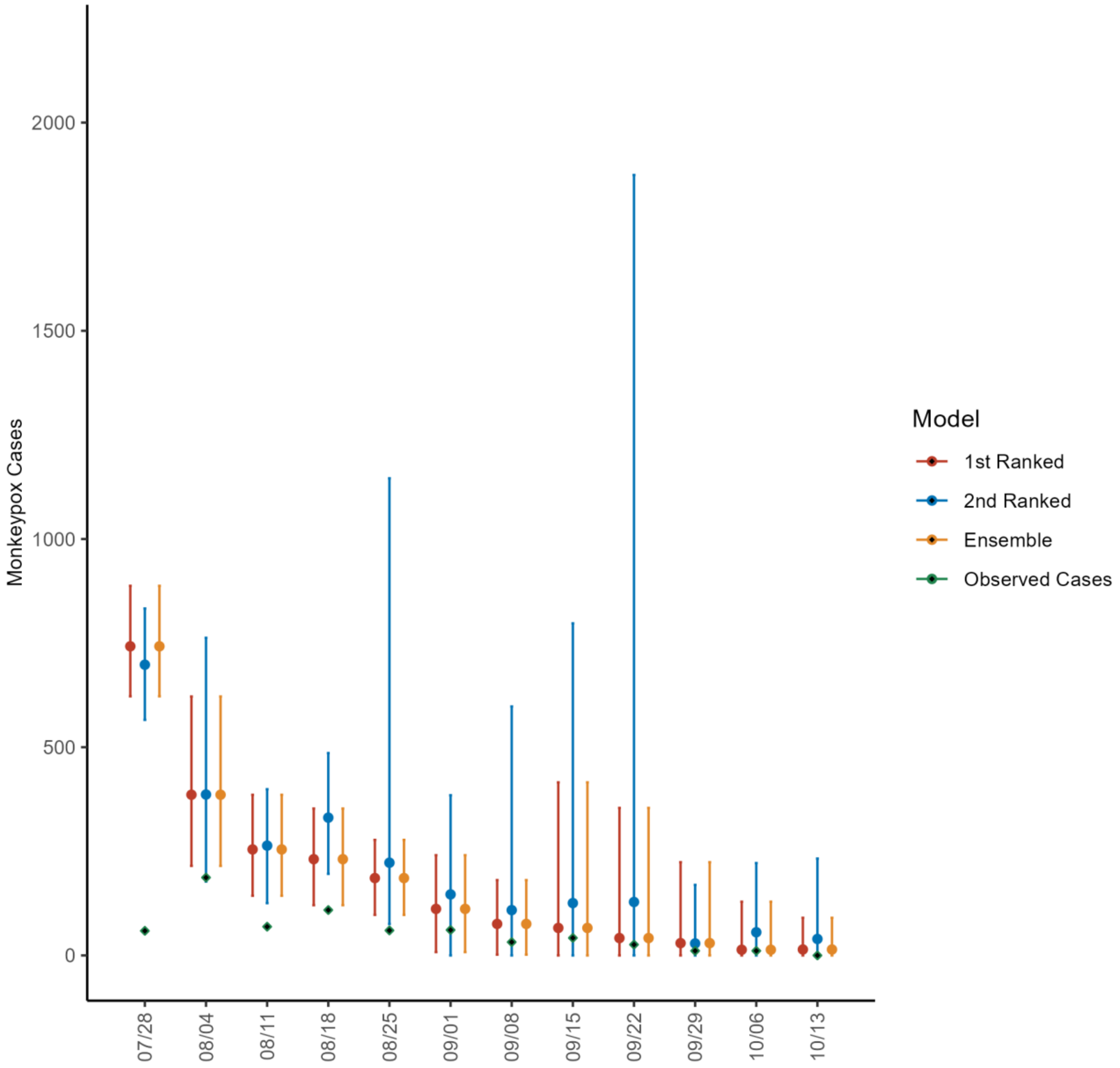

Figure 46s. Predicted cumulative number of new monkeypox cases for each of the 4-week ahead forecasts from the weeks of 7/28/2022 through 10/13/2022 based on a 10-week calibration period for Canada. The solid circles indicate the estimated cumulative median value, and the bars indicate the corresponding 95% prediction interval. The observed cases are as reported to the OWID team as of 10/21/2022 [25]. For the week of 7/28/2022, data posted by the OWID team on 8/9/2022 was used to produce the forecast as it was the earliest version of data available.
