# Supplementary material for: Real-time forecasting the trajectory of monkeypox outbreaks at the national and global levels, July–October 2022
Source: BMC Med. 2023 Jan 16;21:19. doi: 10.1186/s12916-022-02725-2 (PMC9841951; doi:10.1186/s12916-022-02725-2)
Supplement: Supplementary file 2 — Additional file 2: Supplementary Tables. Additional tables related to the dissemination and evaluation of models used to produce the forecasts included within the study. [file 12916_2022_2725_MOESM2_ESM.pdf]

Additional file 2: Supplementary tables

Week	Model	Brazil	Canada	England	France	Germany	Spain	US (OWID)	US (CDC)	World
07-28-22	1st ranked	**1307.40**	90.99	**87.36**	174.71	40.90	**176.55**	**1876.66**	**1653.73**	**1152.49**
	2nd ranked	3228.21	**82.70**	91.80	**172.82**	114.07	341.63	2195.80	1941.08	1241.66
	Ensemble (Weighted)	**1307.40**	90.99	**87.36**	174.71	40.90	**176.55**	**1876.66**	**1653.73**	**1152.49**
	Ensemble (Unweighted)	1501.62	86.74	88.42	**172.53**	**29.31**	236.46	2119.44	1905.29	1170.79
08-04-22	1st ranked	513.10	**24.25**	**99.60**	204.65	23.44	195.65	5524.23	**5600.15**	2961.41
	2nd ranked	**483.25**	24.47	107.57	**162.16**	**22.90**	**106.63**	**1220.59**	17132.86	2577.23
	Ensemble (Weighted)	513.10	**24.25**	**99.60**	204.65	23.44	195.65	5524.23	**5600.15**	2961.41
	Ensemble (Unweighted)	485.83	24.76	102.62	184.40	24.12	108.30	2724.37	6737.27	**2565.84**
08-11-22	1st ranked	116.98	**11.86**	**48.94**	206.31	**22.16**	**48.18**	1045.84	838.32	511.33
	2nd ranked	135.49	13.76	76.46	**180.87**	73.47	185.03	**407.53**	**700.86**	423.42
	Ensemble (Weighted)	116.98	**11.86**	**48.94**	206.31	**22.16**	**48.18**	1045.84	838.32	511.33
	Ensemble (Unweighted)	**102.19**	12.95	60.27	196.99	30.02	102.43	742.33	775.69	**384.81**
08-18-22	1st ranked	376.04	**9.7**	**11.77**	228.39	28.67	**60.4**	676.85	477.58	**645.08**
	2nd ranked	**341.39**	33.19	30.48	**215.18**	43.62	399.73	712.06	**203.62**	885.96
	Ensemble (Weighted)	376.04	**9.7**	**11.77**	228.39	**28.67**	**60.4**	676.85	477.58	**645.08**
	Ensemble (Unweighted)	362.89	22.95	14.42	225.5	36.77	364.3	**556.96**	422.07	746.89
08-25-22	1st ranked	154.1	**7.09**	19.13	22.54	**31.17**	31.84	972.14	377.49	716.45
	2nd ranked	133.98	15.04	**10.83**	31.33	54.38	74.27	**821.92**	**292.29**	**696.84**
	Ensemble (Weighted)	154.1	**7.09**	19.13	22.54	**31.17**	31.84	972.14	377.49	716.45
	Ensemble (Unweighted)	**128.79**	9.5	12.95	**19.16**	39.88	**24.53**	892.96	331.98	729.9
09-01-22	1st ranked	**226.82**	**7.22**	11.79	37.14	**6.22**	73.35	776.82	208.93	823.08
	2nd ranked	244.09	10.55	11.35	34.63	18.85	39.74	**256.01**	**126.26**	677.37
	Ensemble (Weighted)	**226.82**	**7.22**	11.79	37.14	**6.22**	73.35	776.82	208.93	823.08
	Ensemble (Unweighted)	237.09	7.99	**11.17**	**16.47**	9.70	**13.50**	621.87	184.41	**418.90**
09-08-22	1st ranked	157.48	5.85	**11.49**	43.49	11.67	12.21	642.87	164.77	1120.58
	2nd ranked	156.00	7.82	22.72	**20.63**	**6.42**	**10.95**	**588.87**	**149.85**	**994.90**
	Ensemble (Weighted)	157.48	5.85	**11.49**	43.49	11.67	12.21	642.87	164.77	1120.58
	Ensemble (Unweighted)	**110.68**	**5.83**	13.52	22.76	10.37	11.30	618.69	156.78	1054.48
09-15-22	1st ranked	**171.27**	**4.09**	16.82	35.83	6.28	14.54	**368.93**	**36.40**	**523.75**
	2nd ranked	1272.59	15.74	20.83	**23.62**	**4.55**	75.21	938.93	236.66	3730.62
	Ensemble (Weighted)	**171.27**	**4.09**	16.82	35.83	6.28	14.54	**368.93**	**36.40**	**523.75**
	Ensemble (Unweighted)	337.08	5.59	**15.03**	25.13	5.58	**8.27**	404.45	91.64	1050.04

Table 1s. Mean absolute errors (MAE) metric of the forecasts generated by the sub-epidemic models. The mean absolute errors (MAE) metric of the forecasts generated by the sub-epidemic models over 8 sequential forecasting periods and for each geographical area. Values highlighted in bold correspond to best performing model during the forecasting period for a given area. There may be more than one value bolded for a given forecast period and country. This indicates that the bolded models performed equally well. Only forecast periods in which observed data was available are included.

Week	Model	Brazil	Canada	England	France	Germany	Spain	US (OWID)	US (CDC)	World
7-28-22	1st ranked	**2364049.19**	10320.24	**11615.61**	80048.99	2506.33	**39129.18**	**3841147.07**	**2778792.34**	**1597604.21**
	2nd ranked	16925765.93	**8276.33**	12500.64	**70993.28**	15632.66	137353.23	5009878.03	3792893.47	1804185.50
	Ensemble (Weighted)	**2364049.19**	10320.24	**11615.61**	80048.99	2506.33	**39129.18**	**3841147.07**	**2778792.34**	**1597604.21**
	Ensemble (Unweighted)	3184897.05	9230.12	11903.15	75643.17	**1151.32**	71912.88	4739459.68	3648248.53	1617873.05
8-04-22	1st ranked	322108.04	859.00	**12512.59**	90471.12	617.31	43823.72	40217736.19	**38797623.51**	10893647.67
	2nd ranked	**258364.19**	**850.35**	13646.66	**60875.53**	678.16	**16511.82**	**2313218.19**	457651393.86	**6650709.10**
	Ensemble (Weighted)	322108.04	859.00	**12512.59**	90471.12	617.31	43823.72	40217736.19	**38797623.51**	10893647.67
	Ensemble (Unweighted)	265627.4	878.62	12801.13	74574.77	**593.18**	16328.29	8500892.42	57281239.98	7048019.09
8-11-22	1st ranked	20771.72	**176.02**	**4983.70**	92537.33	**742.54**	**2872.34**	1152686.27	711359.42	435608.17
	2nd ranked	28835.70	262.01	6517.98	**77711.78**	6589.97	37471.21	**203317.81**	**504943.73**	**206499.89**
	Ensemble (Weighted)	20771.72	**176.02**	**4983.70**	92537.33	**742.54**	**2872.34**	1152686.27	711359.42	435608.17
	Ensemble (Unweighted)	**18051.77**	218.02	5119.08	88428.49	1427.14	14396.77	614876.18	609758.03	231478.04
8-18-22	1st ranked	179673.13	**153.26**	**261.77**	95708.70	**1128.73**	**4949.63**	736308.70	245904.12	**727711.73**
	2nd ranked	**148790.82**	1195.22	1084.46	**88929.44**	2369.84	161896.62	770797.59	**79446.26**	1292512.11
	Ensemble (Weighted)	179673.13	**153.26**	**261.77**	95708.70	**1128.73**	**4949.63**	736308.70	245904.12	**727711.73**
	Ensemble (Unweighted)	165001.10	594.81	314.37	94223.70	1754.56	134229.87	**598252.85**	200615.58	898035.03
8-25-22	1st ranked	41954.48	**98.71**	483.35	805.69	**1168.14**	1574.15	1173259.62	152750.92	813000.99
	2nd ranked	28943.29	284.95	**143.33**	1538.58	2987.99	6369.42	**888425.7711**	**94560.05**	**790633.89**
	Ensemble (Weighted)	41954.48	**98.71**	483.35	805.69	**1168.14**	1574.15	1173259.62	152750.92	813000.99
	Ensemble (Unweighted)	**27364.56**	151.04	287.80	**403.64**	1719.40	**1125.78**	1015576.84	120862.39	834190.66
9-01-22	1st ranked	**73078.49**	**61.98**	250.16	1792.28	**78.89**	5876.02	714440.86	56435.89	992196.47
	2nd ranked	81670.49	144.59	**170.67**	1772.04	399.19	2015.50	**100213.29**	**21634.49**	497612.85
	Ensemble (Weighted)	**73078.49**	**61.98**	250.16	1792.28	**78.89**	5876.02	714440.86	56435.89	992196.47
	Ensemble (Unweighted)	78784.07	80.44	220.81	**510.22**	143.79	**264.46**	493508.74	46412.09	**326677.47**
9-08-22	1st ranked	35058.06	58.73	**182.38**	2494.38	146.86	272.99	565025.46	28528.03	1777396.54
	2nd ranked	25019.09	76.08	725.15	**507.58**	**51.37**	**144.12**	**457688.84**	**23812.47**	**1383510.77**
	Ensemble (Weighted)	35058.06	58.73	**182.38**	2494.38	146.86	272.99	565025.46	28528.03	1777396.54
	Ensemble (Unweighted)	**23324.14**	**46.95**	204.79	998.69	117.86	166.66	519936.15	25883.95	1632965.52
9-15-22	1st ranked	**31512.15**	**23.23**	380.81	1941.18	60.25	278.07	**172498.66**	**1708.39**	**395043.07**
	2nd ranked	1813357.91	276.42	699.37	**664.36**	**23.13**	5720.43	1184513.80	57656.67	15477666.88
	Ensemble (Weighted)	**31512.15**	**23.23**	380.81	1941.18	60.25	278.07	**172498.66**	1708.39	**395043.07**
	Ensemble (Unweighted)	121858.32	44.39	**260.88**	1065.67	44.77	**76.74**	216304.99	8734.33	1467433.58

Table 2s. Mean square errors (MSE) metric of the forecasts generated by the sub-epidemic models. The mean square errors (MSE) metric of the forecasts generated by the sub-epidemic models over 8 sequential forecasting periods and for each geographical area. Values highlighted in bold correspond to best performing model during the forecasting period for a given area. There may be more than one value bolded for a given forecast period and country. This indicates that the bolded models performed equally well. Only forecast periods in which observed data was available are included.

Week	Model	Brazil	Canada	England	France	Germany	Spain	US (OWID)	US (CDC)	World
07-28-22	1st ranked	**0.00**	**25.00**	**75.00**	**75.00**	**100.00**	**100.00**	**100.00**	**75.00**	**100.00**
	2nd ranked	**0.00**	**25.00**	**75.00**	**75.00**	**100.00**	75.00	0.00	0.00	**100.00**
	Ensemble (Weighted)	**0.00**	**25.00**	**75.00**	**75.00**	**100.00**	**100.00**	**100.00**	**75.00**	**100.00**
	Ensemble (Unweighted)	**0.00**	**25.00**	**75.00**	**75.00**	**100.00**	**100.00**	75.00	0.00	**100.00**
08-04-22	1st ranked	**25.00**	**75.00**	**75.00**	**75.00**	**100.00**	**100.00**	0.00	0.00	50.00
	2nd ranked	0.00	**75.00**	**75.00**	**75.00**	**100.00**	**100.00**	**100.00**	**75.00**	**75.00**
	Ensemble (Weighted)	**25.00**	**75.00**	**75.00**	**75.00**	**100.00**	**100.00**	0.00	0.00	50.00
	Ensemble (Unweighted)	**25.00**	**75.00**	**75.00**	**75.00**	**100.00**	**100.00**	**75.00**	**75.00**	**75.00**
08-11-22	1st ranked	**100.00**	**100.00**	75.00	75.00	**100.00**	**100.00**	**100.00**	0.00	**100.00**
	2nd ranked	75.00	**100.00**	**100.00**	**100.00**	**100.00**	**100.00**	**100.00**	**25.00**	**100.00**
	Ensemble (Weighted)	**100.00**	**100.00**	75.00	75.00	**100.00**	**100.00**	**100.00**	0.00	**100.00**
	Ensemble (Unweighted)	**100.00**	**100.00**	75.00	**100.00**	**100.00**	**100.00**	**100.00**	**25.00**	**100.00**
08-18-22	1st ranked	75.00	**100.00**	**100.00**	**75.00**	**100.00**	**100.00**	**100.00**	25.00	**100.00**
	2nd ranked	75.00	50.00	**100.00**	**75.00**	**100.00**	75.00	**100.00**	**100.00**	**100.00**
	Ensemble (Weighted)	75.00	**100.00**	**100.00**	**75.00**	**100.00**	**100.00**	**100.00**	25.00	**100.00**
	Ensemble (Unweighted)	**100.00**	**100.00**	**100.00**	**75.00**	**100.00**	**100.00**	**100.00**	**100.00**	**100.00**
08-25-22	1st ranked	**100.00**	**100.00**	**100.00**	**100.00**	**100.00**	**100.00**	75.00	50.00	**100.00**
	2nd ranked	**100.00**	**100.00**	**100.00**	**100.00**	50.00	**100.00**	**100.00**	**100.00**	**100.00**
	Ensemble (Weighted)	**100.00**	**100.00**	**100.00**	**100.00**	**100.00**	**100.00**	75.00	50.00	**100.00**
	Ensemble (Unweighted)	**100.00**	**100.00**	**100.00**	**100.00**	75.00	**100.00**	**100.00**	**100.00**	**100.00**
09-01-22	1st ranked	75.00	**100.00**	**100.00**	**100.00**	**100.00**	**100.00**	50.00	**100.00**	75.00
	2nd ranked	**100.00**	**100.00**	**100.00**	**100.00**	**100.00**	**100.00**	**100.00**	**100.00**	**100.00**
	Ensemble (Weighted)	75.00	**100.00**	**100.00**	**100.00**	**100.00**	**100.00**	50.00	**100.00**	75.00
	Ensemble (Unweighted)	75.00	**100.00**	**100.00**	**100.00**	**100.00**	**100.00**	**100.00**	**100.00**	**100.00**
09-08-22	1st ranked	75.00	**100.00**	**100.00**	**100.00**	**100.00**	**100.00**	50.00	**100.00**	50.00
	2nd ranked	**100.00**	**100.00**	**100.00**	**100.00**	**100.00**	**100.00**	**100.00**	**100.00**	**75.00**
	Ensemble (Weighted)	75.00	**100.00**	**100.00**	**100.00**	**100.00**	**100.00**	50.00	**100.00**	50.00
	Ensemble (Unweighted)	**100.00**	**100.00**	**100.00**	**100.00**	**100.00**	**100.00**	**100.00**	**100.00**	**75.00**
09-15-22	1st ranked	**100.00**	**100.00**	**100.00**	**100.00**	**100.00**	**100.00**	**100.00**	**100.00**	**100.00**
	2nd ranked	**100.00**	**100.00**	**100.00**	**100.00**	**100.00**	**100.00**	**100.00**	**100.00**	75.00
	Ensemble (Weighted)	**100.00**	**100.00**	**100.00**	**100.00**	**100.00**	**100.00**	**100.00**	**100.00**	**100.00**
	Ensemble (Unweighted)	**100.00**	**100.00**	**100.00**	**100.00**	**100.00**	**100.00**	**100.00**	**100.00**	**100.00**

Table 3s. Percent coverage of the 95% PI metric of the forecasts generated by the sub-epidemic models. The percent coverage of the 95% prediction interval (PI) metric of the forecasts generated by the sub-epidemic models over 8 sequential forecasting periods and for each geographical area. Values highlighted in bold correspond to best performing model during the forecasting period for a given area. There may be more than one value bolded for a given forecast period and country. This indicates that the bolded models performed equally well. Only forecast periods in which observed data was available are included.

Week	Model	Brazil	Canada	England	France	Germany	Spain	US (OWID)	US (CDC)	World
07-28-22	1st ranked	**1040.18**	79.75	60.75	**120.88**	**24.55**	**93.64**	**1107.61**	**1013.06**	**628.79**
	2nd ranked	2128.96	**69.82**	62.24	126.69	70.97	225.15	1835.73	1891.16	720.81
	Ensemble (Weighted)	**1040.18**	79.75	60.75	**120.88**	**24.55**	**93.64**	**1107.61**	**1013.06**	**628.79**
	Ensemble (Unweighted)	1384.18	74.18	**60.48**	122.32	29.70	113.98	1376.73	1311.82	676.83
08-04-22	1st ranked	**394.05**	16.48	**67.01**	155.85	13.51	91.93	4414.97	**4580.72**	1915.62
	2nd ranked	447.96	**15.76**	69.17	**126.96**	13.51	92.72	**697.57**	9284.33	**1610.64**
	Ensemble (Weighted)	**394.05**	16.48	**67.01**	155.85	13.51	91.93	4414.97	**4580.72**	1915.62
	Ensemble (Unweighted)	412.27	16.04	68.13	138.94	**13.13**	**81.48**	1498.48	5567.13	1637.65
08-11-22	1st ranked	71.68	**6.91**	**35.46**	161.83	**14.44**	**62.34**	550.30	712.04	358.12
	2nd ranked	96.94	8.33	42.90	**116.03**	43.00	115.02	**287.13**	**479.62**	**244.81**
	Ensemble (Weighted)	71.68	**6.91**	**35.46**	161.83	**14.44**	**62.34**	550.30	712.04	358.12
	Ensemble (Unweighted)	**57.72**	7.68	37.68	131.32	24.28	83.68	388.21	580.48	279.50
08-18-22	1st ranked	255.27	**6.69**	**9.42**	177.93	**18.41**	**66.74**	**386.12**	339.92	**405.01**
	2nd ranked	**217.68**	22.41	23.92	**147.73**	29.29	261.16	621.02	337.29	510.20
	Ensemble (Weighted)	255.27	**6.69**	**9.42**	177.93	**18.41**	**66.74**	**386.12**	339.92	**405.01**
	Ensemble (Unweighted)	234.53	11.95	14.37	161.09	22.95	141.33	430.33	**270.76**	446.24
08-25-22	1st ranked	**91.88**	**4.96**	10.85	**47.33**	**18.90**	51.96	628.12	234.08	441.09
	2nd ranked	132.93	10.40	**8.37**	71.68	33.98	**44.06**	**424.17**	**155.30**	**433.50**
	Ensemble (Weighted)	**91.88**	**4.96**	10.85	**47.33**	**18.90**	51.96	628.12	234.08	441.09
	Ensemble (Unweighted)	95.80	7.21	8.95	55.93	25.19	45.58	511.04	193.22	435.97
09-01-22	1st ranked	160.56	**4.69**	**7.77**	46.40	**7.88**	48.60	465.91	127.59	530.15
	2nd ranked	**144.22**	7.42	8.54	**43.69**	15.75	**26.65**	466.12	127.35	392.40
	Ensemble (Weighted)	160.56	**4.69**	**7.77**	46.40	**7.88**	48.60	465.91	127.59	530.15
	Ensemble (Unweighted)	150.66	5.74	**7.77**	45.07	10.83	31.94	**380.37**	**115.15**	**333.79**
09-08-22	1st ranked	100.51	**4.12**	**7.13**	**41.14**	6.31	**17.10**	399.84	**91.87**	814.40
	2nd ranked	122.09	6.25	14.66	49.34	**5.13**	28.37	**329.40**	113.29	**626.04**
	Ensemble (Weighted)	100.51	**4.12**	**7.13**	**41.14**	6.31	**17.10**	399.84	**91.87**	814.40
	Ensemble (Unweighted)	**89.02**	4.77	9.71	45.47	5.15	21.55	363.42	94.89	698.72
09-15-22	1st ranked	**95.61**	**7.80**	**13.45**	**39.02**	**5.92**	**14.13**	**216.45**	**46.88**	**329.59**
	2nd ranked	678.89	14.18	29.16	47.79	11.11	47.46	1733.14	162.57	2137.17
	Ensemble (Weighted)	**95.61**	**7.80**	**13.45**	**39.02**	**5.92**	**14.13**	**216.45**	**46.88**	**329.59**
	Ensemble (Unweighted)	272.38	10.66	19.78	42.34	7.96	24.93	722.95	87.13	844.38

Table 4s. Weighted Interval Score (WIS) metric of the forecasts generated by the sub-epidemic models. The weighted Interval Score (WIS) metric of the forecasts generated by the sub-epidemic models over 8 sequential forecasting periods and for each geographical area. Values highlighted in bold correspond to best performing model during the forecasting period for a given area. There may be more than one value bolded for a given forecast period and country. This indicates that the bolded models performed equally well. Only forecast periods in which observed data was available are included.

	**Model**	**Brazil**	**Canada**	**England**	**France**	**Germany**	**Spain**	**US (OWID)**	**US (CDC)**	**World**
Mean Absolute Error (MAE)	1st ranked*	**37.50**	**75.00**	**62.50**	0.00	**50.00**	**37.50**	25.00	37.50	**37.50**
	2nd ranked	25.00	12.50	12.50	**62.50**	37.50	25.00	**62.50**	**62.50**	25.00
	Ensemble* (Weighted)	**37.50**	**75.00**	**62.50**	0.00	**50.00**	**37.50**	25.00	37.50	**37.50**
	Ensemble (Unweighted)	**37.50**	12.50	25.00	37.50	12.50	**37.50**	12.50	0.00	**37.50**
Mean Square Error (MSE)	1st ranked*	**37.50**	**62.50**	**62.50**	0.00	**50.00**	37.50	25.00	37.50	37.50
	2nd ranked	25.00	25.00	25.00	**75.00**	25.00	12.50	**62.50**	**62.50**	**50.00**
	Ensemble* (Weighted)	**37.50**	**62.50**	**62.50**	0.00	**50.00**	37.50	25.00	37.50	37.50
	Ensemble (Unweighted)	**37.50**	12.50	12.50	25.00	25.00	**50.00**	12.50	0.00	12.50
Percent coverage of the 95% prediction interval (PI)	1st ranked*	62.50	**100.00**	87.50	87.50	**100.00**	**100.00**	50.00	50.00	62.50
	2nd ranked	62.50	87.50	**100.00**	**100.00**	87.50	75.00	**87.50**	**87.50**	87.50
	Ensemble* (Weighted)	62.50	**100.00**	87.50	87.50	**100.00**	**100.00**	50.00	50.00	62.50
	Ensemble (Unweighted)	**87.50**	**100.00**	87.50	**100.00**	87.50	**100.00**	75.00	**87.50**	**100.00**
Weighted Interval Score (WIS)	1st ranked*	**50.00**	**75.00**	**75.00**	**50.00**	**75.00**	**62.50**	37.50	**50.00**	37.50
	2nd ranked	25.00	25.00	12.50	**50.00**	12.50	25.00	**50.00**	25.00	**50.00**
	Ensemble* (Weighted)	**50.00**	**75.00**	**75.00**	**50.00**	**75.00**	**62.50**	37.50	**50.00**	37.50
	Ensemble (Unweighted)	25.00	0.00	25.00	0.00	12.50	12.50	12.50	25.00	12.50

Table 5s. Frequency a given model ranked the best for a given metric. The frequency a given model ranked the best for a given metric generate for forecasts over the course of 8 sequential forecasting periods (July 28th – September 15th, 2022) for geographic regions included in the study. Frequencies are listed as the percent out of 100. *The 1st ranked, and weighted ensemble forecasts were the exact same for every given metric and geographical region.

	**Model**	**Brazil***	**Canada***	**England***	**France***	**Germany***	**Spain***	**US* (OWID)**	**US† (CDC)**	**World***
Mean Absolute Error (MAE)	Top-ranked	45.52	9.74	22.74	67.98	17.52	111.94	210.74	112.45	270.87
	2nd ranked	**38.25**	**8.80**	**21.57**	**61.85**	**14.85**	**82.73**	**181.08**	**97.51**	**236.24**
	Ensemble (Weighted)	45.52	9.74	22.74	67.98	17.52	111.94	210.74	112.45	270.87
	Ensemble (Unweighted)	41.21	9.34	21.94	62.67	15.40	97.06	191.19	102.44	250.32
Mean Square Error (MSE)	Top-ranked	3833.94	**220.99**	1215.30	12303.24	615.95	23202.07	93478.23	26323.77	123124.47
	2nd ranked	**2795.90**	224.38	**1118.59**	**10393.44**	**446.15**	**13740.62**	**75456.03**	**20300.88**	**92214.48**
	Ensemble (Weighted)	3833.94	**220.99**	1215.30	12303.24	615.95	23202.07	93478.23	26323.77	123124.47
	Ensemble (Unweighted)	3150.86	221.88	1159.47	11197.14	483.93	17227.40	81207.31	21724.52	99542.69
Percent coverage of the 95% prediction interval (PI)	Top-ranked	**100.00**	**100.00**	**100.00**	97.50	**100.00**	**100.00**	**100.00**	**100.00**	**100.00**
	2nd ranked	**100.00**	**100.00**	**100.00**	**100.00**	**100.00**	**100.00**	**100.00**	**100.00**	**100.00**
	Ensemble (Weighted)	**100.00**	**100.00**	**100.00**	97.50	**100.00**	**100.00**	**100.00**	**100.00**	**100.00**
	Ensemble (Unweighted)	**100.00**	**100.00**	**100.00**	**100.00**	**100.00**	**100.00**	**100.00**	**100.00**	**100.00**
Weighted Interval Score (WIS)	Top-ranked	28.15	**6.01**	**14.95**	**47.96**	11.62	70.21	143.71	72.45	166.61
	2nd ranked	26.98	6.53	16.11	50.91	10.98	**62.26**	142.61	70.46	164.27
	Ensemble (Weighted)	28.15	**6.01**	**14.95**	**47.96**	11.62	70.21	143.71	72.45	166.61
	Ensemble (Unweighted)	**26.67**	6.15	15.26	48.42	**10.89**	63.41	**139.32**	**68.27**	**159.34**

Table 6s. Mean performance metrics quantifying model fit. The mean performance metrics quantifying model fit across 20 sequential 10-week calibration periods (Week of May 26th – October 13th, 2022) using weekly timeseries data from the CDC[†] and OWID[*] teams for each geographical region. Values highlighted in bold correspond to best performing model during the forecasting period for a given area. There may be more than one value bolded for a given forecast period and country. This indicates that the bolded models performed equally well. Only forecast periods in which observed data was available are included.

Week	Model	Brazil	Canada	England	France	Germany	Spain	US (OWID)	US (CDC)	World
07-28-22	1st ranked	21.64	6.03	**26.54**	36.44	22.26	138.62	123.71	138.91	**146.21**
	2nd ranked	**19.56**	**4.37**	26.95	**35.65**	**13.63**	**104.59**	**74.53**	**20.75**	146.53
	Ensemble (Weighted)	21.64	6.03	**26.54**	36.44	22.26	138.62	123.71	138.91	**146.21**
	Ensemble (Unweighted)	20.23	5.03	26.62	36.51	17.53	117.97	86.41	55.22	147.68
08-04-22	1st ranked	32.06	7.54	28.73	42.42	21.80	133.46	162.67	173.45	175.95
	2nd ranked	**13.08**	**7.39**	**23.94**	**30.07**	**12.21**	129.05	**128.86**	**167.53**	**154.76**
	Ensemble (Weighted)	32.06	7.54	28.73	42.42	21.80	133.46	162.67	173.45	175.95
	Ensemble (Unweighted)	19.48	7.57	26.86	35.70	13.65	**126.69**	148.16	170.89	163.80
08-11-22	1st ranked	29.97	6.75	27.21	**47.67**	17.05	131.51	**210.55**	59.41	269.48
	2nd ranked	**25.05**	**5.16**	**22.75**	50.76	17.39	**106.04**	218.29	**52.98**	**214.71**
	Ensemble (Weighted)	29.97	6.75	27.21	**47.67**	17.05	131.51	**210.55**	59.41	269.48
	Ensemble (Unweighted)	27.27	6.05	25.12	48.81	**16.67**	115.69	216.37	58.57	250.58
08-18-22	1st ranked	36.75	7.31	20.09	41.20	17.06	132.46	229.51	79.16	252.74
	2nd ranked	**30.66**	**6.21**	**18.82**	**39.85**	**13.31**	**81.08**	**221.78**	**76.35**	**222.85**
	Ensemble (Weighted)	36.75	7.31	20.09	41.20	17.06	132.46	229.51	79.16	252.74
	Ensemble (Unweighted)	33.41	6.88	19.74	42.07	14.48	116.62	222.58	77.81	241.85
08-25-22	1st ranked	47.44	6.29	15.60	101.45	15.21	133.56	229.45	**82.96**	319.08
	2nd ranked	**44.26**	**5.12**	**12.04**	**94.66**	**12.28**	**95.30**	221.37	90.28	**292.14**
	Ensemble (Weighted)	47.44	6.29	15.60	101.45	15.21	133.56	229.45	**82.96**	319.08
	Ensemble (Unweighted)	44.77	5.58	12.63	95.60	13.34	116.65	**221.15**	86.33	306.17
09-01-22	1st ranked	46.08	10.37	**10.77**	93.91	**17.92**	116.77	260.35	**95.67**	340.84
	2nd ranked	**42.81**	**9.97**	11.30	89.97	20.50	**42.21**	**220.45**	101.14	**289.47**
	Ensemble (Weighted)	46.08	10.37	**10.77**	93.91	**17.92**	116.77	260.35	**95.67**	340.84
	Ensemble (Unweighted)	43.42	10.15	10.84	**80.17**	18.73	77.48	241.06	98.09	296.05
09-08-22	1st ranked	55.98	8.05	12.05	90.95	**6.51**	59.83	243.95	**138.23**	267.55
	2nd ranked	**51.01**	**7.07**	12.00	**78.91**	6.94	**57.48**	245.35	142.29	**257.11**
	Ensemble (Weighted)	55.98	8.05	12.05	90.95	**6.51**	59.83	243.95	**138.23**	267.55
	Ensemble (Unweighted)	52.96	7.70	**11.73**	81.74	6.53	58.32	**242.55**	140.89	264.78
09-15-22	1st ranked	94.28	25.60	**40.95**	89.78	22.38	49.27	225.75	131.85	395.12
	2nd ranked	**79.60**	**25.08**	44.78	**74.89**	22.51	**46.09**	**118.04**	**128.79**	**312.35**
	Ensemble (Weighted)	94.28	25.60	**40.95**	89.78	22.38	49.27	225.75	131.85	395.12
	Ensemble (Unweighted)	88.13	25.78	41.95	80.77	**22.30**	47.05	151.23	131.69	331.61

Table 7s. Mean absolute error (MAE) metrics quantifying model fit. The mean absolute error (MAE) metrics quantifying model fit across 20 sequential 10-week calibration periods (Week of May 26th – October 13th, 2022) used during forecasting periods for the given geographical regions. Values highlighted in bold correspond to best performing model during the forecasting period for a given area. There may be more than one value bolded for a given forecast period and country. This indicates that the bolded models performed equally well. Only forecast periods in which observed data was available are included.

Week	Model	Brazil	Canada	England	France	Germany	Spain	US (OWID)	US (CDC)	World
07-28-22	1st ranked	797.18	69.02	**1084.19**	2964.07	936.01	32577.00	37426.62	41061.16	35401.61
	2nd ranked	**517.01**	**38.92**	1130.97	**2396.74**	**266.33**	**23107.15**	**11310.19**	**993.61**	**34107.69**
	Ensemble (Weighted)	797.18	69.02	**1084.19**	2964.07	936.01	32577.00	37426.62	41061.16	35401.61
	Ensemble (Unweighted)	638.21	50.24	1097.57	2609.83	460.37	26537.40	16583.00	6469.11	35066.30
08-04-22	1st ranked	1778.58	89.70	1210.71	2986.61	815.99	31839.75	73858.07	74580.14	52914.01
	2nd ranked	**328.41**	**87.18**	**892.64**	**1377.14**	**296.36**	**26130.86**	**42452.64**	**63253.40**	**33827.46**
	Ensemble (Weighted)	1778.58	89.70	1210.71	2986.61	815.99	31839.75	73858.07	74580.14	52914.01
	Ensemble (Unweighted)	598.69	90.25	1087.66	2097.18	410.45	28603.31	59515.87	70969.79	41772.18
08-11-22	1st ranked	2037.12	64.00	1160.27	**3400.90**	610.48	31954.23	101526.93	6266.19	103584.14
	2nd ranked	**1029.36**	**45.50**	**841.49**	3673.42	597.38	**19598.69**	**99956.47**	**6178.18**	**73762.56**
	Ensemble (Weighted)	2037.12	64.00	1160.27	**3400.90**	610.48	31954.23	101526.93	6266.19	103584.14
	Ensemble (Unweighted)	1415.44	53.21	1026.27	3475.13	**566.17**	23555.35	102836.07	6335.19	89546.09
08-18-22	1st ranked	2180.36	74.93	**645.32**	**2746.61**	512.74	32008.76	104611.47	10241.78	107034.19
	2nd ranked	**1976.88**	**62.10**	645.74	2753.31	**439.24**	**15225.73**	102405.02	10154.03	**83580.23**
	Ensemble (Weighted)	2180.36	74.93	**645.32**	**2746.61**	512.74	32008.76	104611.47	10241.78	107034.19
	Ensemble (Unweighted)	2035.91	67.27	651.19	2789.54	469.14	23514.26	**102339.35**	**9852.72**	95498.10
08-25-22	1st ranked	3629.89	58.78	380.95	22578.97	492.25	29041.11	107625.64	**14990**.15	145361.68
	2nd ranked	**2632.41**	**53.06**	268.86	**17902.65**	**279.30**	**14942.30**	**100406.00**	15976.68	**116437.94**
	Ensemble (Weighted)	3629.89	58.78	380.95	22578.97	492.25	29041.11	107625.64	**14990.15**	145361.68
	Ensemble (Unweighted)	3001.05	54.66	**265.87**	20042.38	369.12	20419.05	103700.44	15331.37	133545.45
09-01-22	1st ranked	3622.06	**147.75**	**245.07**	21616.42	**740.96**	19695.80	116488.17	17498.43	174865.61
	2nd ranked	**2933.55**	151.40	257.32	**17678.92**	838.36	**3412.73**	110619.95	**17271.81**	142329.80
	Ensemble (Weighted)	3622.06	**147.75**	**245.07**	21616.42	**740.96**	19695.80	116488.17	17498.43	174865.61
	Ensemble (Unweighted)	3056.94	149.02	251.09	18995.35	770.86	7351.48	**108100.31**	17604.84	**121988.23**
09-08-22	1st ranked	4172.76	101.17	270.48	21313.98	111.68	4612.78	114765.99	**24014.42**	107287.31
	2nd ranked	**3745.92**	**96.00**	**254.58**	**20296.42**	114.99	**4316.43**	**107104.49**	26269.55	**105033.78**
	Ensemble (Weighted)	4172.76	101.17	270.48	21313.98	111.68	4612.78	114765.99	**24014.42**	107287.31
	Ensemble (Unweighted)	3822.69	98.69	257.00	20353.34	**110.65**	4413.96	109812.89	25256.66	106099.55
09-15-22	1st ranked	12453.56	**1162.51**	4725.45	20818.35	**707.49**	3887.13	91522.97	**21937.91**	258547.18
	2nd ranked	**9203.64**	1260.88	4657.13	**17068.89**	737.19	**3191.04**	**29393.45**	22309.78	**148636.39**
	Ensemble (Weighted)	12453.56	**1162.51**	4725.45	20818.35	**707.49**	3887.13	91522.97	**21937.91**	258547.18
	Ensemble (Unweighted)	10637.98	1211.67	**4639.07**	19214.38	714.72	3424.40	46770.58	21976.45	172825.59

Table 8s. Mean square error (MSE) metrics quantifying model fit. The mean square error (MSE) metrics quantifying model fit across 20 sequential 10-week calibration periods (Week of May 26th – October 13th, 2022) used during forecasting periods for the given geographical regions. Values highlighted in bold correspond to best performing model during the forecasting period for a given area. There may be more than one value bolded for a given forecast period and country. This indicates that the bolded models performed equally well. Only forecast periods in which observed data was available are included.

**Week**	**Model**	**Brazil**	**Canada**	**England**	**France**	**Germany**	**Spain**	**US (OWID)**	**US (CDC)**	**World**
07-28-22	1st ranked	**100.00**	**100.00**	**100.00**	**100.00**	**100.00**	**100.00**	**100.00**	**100.00**	**100.00**
	2nd ranked	**100.00**	**100.00**	**100.00**	**100.00**	**100.00**	**100.00**	**100.00**	**100.00**	**100.00**
	Ensemble (Weighted)	**100.00**	**100.00**	**100.00**	**100.00**	**100.00**	**100.00**	**100.00**	**100.00**	**100.00**
	Ensemble (Unweighted)	**100.00**	**100.00**	**100.00**	**100.00**	**100.00**	**100.00**	**100.00**	**100.00**	**100.00**
08-04-22	1st ranked	**100.00**	**100.00**	**100.00**	**100.00**	**100.00**	**100.00**	**100.00**	**100.00**	**100.00**
	2nd ranked	**100.00**	**100.00**	**100.00**	**100.00**	**100.00**	**100.00**	**100.00**	**100.00**	**100.00**
	Ensemble (Weighted)	**100.00**	**100.00**	**100.00**	**100.00**	**100.00**	**100.00**	**100.00**	**100.00**	**100.00**
	Ensemble (Unweighted)	**100.00**	**100.00**	**100.00**	**100.00**	**100.00**	**100.00**	**100.00**	**100.00**	**100.00**
08-11-22	1st ranked	**100.00**	**100.00**	**100.00**	**100.00**	**100.00**	**100.00**	**100.00**	**100.00**	**100.00**
	2nd ranked	**100.00**	**100.00**	**100.00**	**100.00**	**100.00**	**100.00**	**100.00**	**100.00**	**100.00**
	Ensemble (Weighted)	**100.00**	**100.00**	**100.00**	**100.00**	**100.00**	**100.00**	**100.00**	**100.00**	**100.00**
	Ensemble (Unweighted)	**100.00**	**100.00**	**100.00**	**100.00**	**100.00**	**100.00**	**100.00**	**100.00**	**100.00**
08-18-22	1st ranked	**100.00**	**100.00**	**100.00**	**100.00**	**100.00**	**100.00**	**100.00**	**100.00**	**100.00**
	2nd ranked	**100.00**	**100.00**	**100.00**	**100.00**	**100.00**	**100.00**	**100.00**	**100.00**	**100.00**
	Ensemble (Weighted)	**100.00**	**100.00**	**100.00**	**100.00**	**100.00**	**100.00**	**100.00**	**100.00**	**100.00**
	Ensemble (Unweighted)	**100.00**	**100.00**	**100.00**	**100.00**	**100.00**	**100.00**	**100.00**	**100.00**	**100.00**
08-25-22	1st ranked	**100.00**	**100.00**	**100.00**	**100.00**	**100.00**	**100.00**	**100.00**	**100.00**	**100.00**
	2nd ranked	**100.00**	**100.00**	**100.00**	**100.00**	**100.00**	**100.00**	**100.00**	**100.00**	**100.00**
	Ensemble (Weighted)	**100.00**	**100.00**	**100.00**	**100.00**	**100.00**	**100.00**	**100.00**	**100.00**	**100.00**
	Ensemble (Unweighted)	**100.00**	**100.00**	**100.00**	**100.00**	**100.00**	**100.00**	**100.00**	**100.00**	**100.00**
09-01-22	1st ranked	**100.00**	**100.00**	**100.00**	**100.00**	**100.00**	**100.00**	**100.00**	**100.00**	**100.00**
	2nd ranked	**100.00**	**100.00**	**100.00**	**100.00**	**100.00**	**100.00**	**100.00**	**100.00**	**100.00**
	Ensemble (Weighted)	**100.00**	**100.00**	**100.00**	**100.00**	**100.00**	**100.00**	**100.00**	**100.00**	**100.00**
	Ensemble (Unweighted)	**100.00**	**100.00**	**100.00**	**100.00**	**100.00**	**100.00**	**100.00**	**100.00**	**100.00**
09-08-22	1st ranked	**100.00**	**100.00**	**100.00**	90.00	**100.00**	**100.00**	**100.00**	**100.00**	**100.00**
	2nd ranked	**100.00**	**100.00**	**100.00**	**100.00**	**100.00**	**100.00**	**100.00**	**100.00**	**100.00**
	Ensemble (Weighted)	**100.00**	**100.00**	**100.00**	90.00	**100.00**	**100.00**	**100.00**	**100.00**	**100.00**
	Ensemble (Unweighted)	**100.00**	**100.00**	**100.00**	**100.00**	**100.00**	**100.00**	**100.00**	**100.00**	**100.00**
09-15-22	1st ranked	**100.00**	**100.00**	**100.00**	90.00	**100.00**	**100.00**	**100.00**	**100.00**	**100.00**
	2nd ranked	**100.00**	**100.00**	**100.00**	**100.00**	**100.00**	**100.00**	**100.00**	**100.00**	**100.00**
	Ensemble (Weighted)	**100.00**	**100.00**	**100.00**	90.00	**100.00**	**100.00**	**100.00**	**100.00**	**100.00**
	Ensemble (Unweighted)	**100.00**	**100.00**	**100.00**	**100.00**	**100.00**	**100.00**	**100.00**	**100.00**	**100.00**

Table 9s. Percent coverage of the 95% (PI) metrics quantifying model fit. The percent coverage of the 95% prediction interval (PI) metrics quantifying model fit across 20 sequential 10-week calibration periods (Week of May 26th – October 13th, 2022) used during forecasting periods for the given geographical regions. Values highlighted in bold correspond to best performing model during the forecasting period for a given area. There may be more than one value bolded for a given forecast period and country. This indicates that the bolded models performed equally well. Only forecast periods in which observed data was available are included.

Week	Model	Brazil	Canada	England	France	Germany	Spain	US (OWID)	US (CDC)	World
07-28-22	1st ranked	**13.38**	4.02	**15.72**	**25.51**	14.43	86.47	86.45	90.11	**92.74**
	2nd ranked	14.76	**3.51**	18.65	26.21	**9.70**	83.34	**58.45**	**18.52**	102.60
	Ensemble (Weighted)	**13.38**	4.02	**15.72**	**25.51**	14.43	86.47	86.45	90.11	**92.74**
	Ensemble (Unweighted)	13.57	3.66	16.96	25.98	11.18	**83.17**	68.32	39.64	95.85
08-04-22	1st ranked	19.76	**4.78**	17.19	26.54	13.84	**85.33**	122.64	124.34	112.68
	2nd ranked	**11.32**	5.22	**15.96**	**20.27**	**8.50**	92.79	**105.21**	126.40	114.76
	Ensemble (Weighted)	19.76	**4.78**	17.19	26.54	13.84	**85.33**	122.64	124.34	112.68
	Ensemble (Unweighted)	13.52	4.93	16.34	22.97	10.24	87.34	112.64	**123.26**	**107.80**
08-11-22	1st ranked	20.97	3.99	16.51	**29.12**	**11.69**	86.20	**143.87**	**37.99**	159.13
	2nd ranked	**17.14**	**3.76**	**16.17**	33.54	13.49	**79.14**	165.48	42.95	148.37
	Ensemble (Weighted)	20.97	3.99	16.51	**29.12**	**11.69**	86.20	**143.87**	**37.99**	159.13
	Ensemble (Unweighted)	18.08	3.81	16.21	30.74	12.23	80.72	150.92	39.19	**148.23**
08-18-22	1st ranked	**23.72**	4.29	**12.52**	**26.06**	11.12	86.47	**157.02**	**49.85**	158.50
	2nd ranked	25.52	4.40	13.82	29.24	11.24	**71.47**	173.19	56.66	163.29
	Ensemble (Weighted)	**23.72**	4.29	**12.52**	**26.06**	11.12	86.47	**157.02**	**49.85**	158.50
	Ensemble (Unweighted)	24.25	**4.25**	12.95	27.34	**10.95**	71.97	161.47	51.94	**156.79**
08-25-22	1st ranked	29.30	**3.80**	9.66	71.59	10.82	82.80	**157.60**	**60.34**	191.08
	2nd ranked	29.30	4.17	9.33	73.98	**8.55**	**68.30**	171.48	69.97	**173.72**
	Ensemble (Weighted)	29.30	**3.80**	9.66	71.59	10.82	82.80	**157.60**	**60.34**	191.08
	Ensemble (Unweighted)	**28.40**	3.91	**8.98**	**71.07**	9.35	72.68	162.33	64.31	179.59
09-01-22	1st ranked	29.73	**6.02**	**7.82**	**69.77**	**13.10**	69.82	**166.63**	**65.03**	206.93
	2nd ranked	**28.80**	6.72	9.05	73.95	15.61	**33.94**	185.35	75.72	214.27
	Ensemble (Weighted)	29.73	**6.02**	**7.82**	**69.77**	**13.10**	69.82	**166.63**	**65.03**	206.93
	Ensemble (Unweighted)	28.92	6.29	8.29	69.98	14.15	45.63	173.94	68.50	**191.47**
09-08-22	1st ranked	**32.31**	**4.97**	**8.19**	**67.95**	**5.13**	**34.11**	**165.41**	**77.75**	**163.05**
	2nd ranked	34.34	5.52	8.89	76.69	5.89	37.05	182.97	88.51	179.88
	Ensemble (Weighted)	**32.31**	**4.97**	**8.19**	**67.95**	**5.13**	**34.11**	**165.41**	**77.75**	**163.05**
	Ensemble (Unweighted)	32.49	5.16	8.46	71.12	5.38	35.04	171.57	81.72	170.66
09-15-22	1st ranked	56.03	**16.20**	**32.01**	**67.17**	**12.84**	**30.46**	150.05	**74.15**	248.77
	2nd ranked	54.65	18.92	37.02	73.39	14.84	32.05	**98.72**	84.95	**217.31**
	Ensemble (Weighted)	56.03	**16.20**	**32.01**	**67.17**	**12.84**	**30.46**	150.05	**74.15**	248.77
	Ensemble (Unweighted)	**54.12**	17.21	33.88	68.16	13.67	30.73	113.33	77.60	224.32

Table 10s. Weighted interval score (WIS) metrics quantifying model fit. The weighted interval score (WIS) metrics quantifying model fit across 20 sequential 10-week calibration periods (Week of May 26th – October 13th, 2022) used during forecasting periods for the given geographical regions. Values highlighted in bold correspond to best performing model during the forecasting period for a given area. There may be more than one value bolded for a given forecast period and country. This indicates that the bolded models performed equally well. Only forecast periods in which observed data was available are included.

Second-Ranked Model										
**Week**		**Brazil**	**Canada**	**England**	**France**	**Germany**	**Spain**	**US (OWID)**	**US (CDC)**	**World**
07-28-22	Median	16102.9	698.1	861.7	935.2	1189.0	3483.8	3448.7	2573.8	20604.8
	LB	6096.9	565.7	186.1	93.8	550.1	1078.3	1679.7	1921.9	13073.0
	UB	39180.3	833.1	2026.9	2350.8	2868.0	6915.3	7389.5	3134.8	30896.7
08-04-22	Median	1295.2	386.4	702.7	795.3	512.5	1835.7	12011.9	72771.3	33511.9
	LB	925.5	177.4	201.7	187.8	180.4	0.0	4640.2	6186.8	19185.0
	UB	1689.7	762.7	1601.6	1529.4	797.9	6369.0	23275.5	159904.1	65275.2
08-11-22	Median	3668.9	263.6	557.5	476.7	651.7	1945.2	8652.7	4803.5	20910.2
	LB	3017.8	125.6	68.4	0.0	145.3	0.0	1790.0	2688.0	15426.7
	UB	4412.5	399.1	1103.9	4121.0	1540.3	5362.3	24943.3	7276.3	27280.5
08-18-22	Median	1770.8	330.8	386.9	261.3	431.2	2516.3	9344.5	5083.5	16790.7
	LB	650.9	196.0	17.4	0.0	97.5	647.2	1288.9	2617.6	10550.5
	UB	3767.3	485.9	2132.6	1210.2	849.1	4402.7	32128.1	11648.3	25842.3
08-25-22	Median	3034.9	222.7	216.5	592.4	362.7	401.5	4296.7	4047.7	15331.8
	LB	1037.6	75.4	15.8	0.0	128.4	0.0	369.0	1682.3	10176.1
	UB	27926.0	1145.7	508.3	12679.9	585.5	2296.1	11480.4	6690.7	21986.0
09-01-22	Median	1615.7	146.5	165.2	305.3	219.0	305.9	7213.8	3184.7	17921.9
	LB	638.5	0.0	0.0	0.0	0.0	0.0	279.2	1740.5	11869.3
	UB	3777.4	384.7	467.5	3053.3	774.3	1179.2	108959.8	4702.4	24638.6
09-08-22	Median	2572.8	108.8	195.2	382.4	90.8	362.6	3592.6	2540.1	9916.2
	LB	765.2	0.0	0.0	0.0	0.0	0.0	0.0	943.9	4243.7
	UB	22643.0	597.8	555.4	2912.5	365.8	1676.5	9732.7	4298.7	21199.2
09-15-22	Median	6784.5	125.7	249.4	304.4	108.8	516.6	7584.4	2502.8	24432.4
	LB	1031.7	0.0	0.0	0.0	0.0	0.0	1426.7	940.1	10430.3
	UB	11736.4	797.4	1759.9	3026.6	793.5	2908.5	283241.3	4173.2	58026.3
09-22-22	Median	1404.9	128.3	121.8	400.8	138.8	159.9	4464.6	2242.4	11317.7
	LB	23.0	0.0	0.0	0.0	0.0	0.0	102.9	411.5	1876.8
	UB	3777.2	1874.2	1363.8	2546.3	956.8	548.2	16090.2	4805.1	1521252.0
09-29-22	Median	942.9	28.7	411.7	271.7	106.6	352.6	5275.8	1759.7	11260.9
	LB	0.0	0.0	0.0	0.0	0.0	0.0	317.9	227.2	1405.9
	UB	4356.1	169.3	1473.2	6776.6	358.0	1031.4	10158.9	3703.1	32533.4
10-06-22	Median	1328.6	55.7	4.4	343.6	126.9	94.6	2005.9	756.3	6075.6
	LB	0.0	0.0	0.0	0.0	0.0	0.0	0.0	84.3	0.0
	UB	6414.1	221.7	599.9	10883.7	638.0	500.0	8133.4	1627.7	15271.4
10-13-22	Median	1044.6	39.5	25.7	70.0	72.5	147.5	1768.7	579.2	3393.7
	LB	0.0	0.0	0.0	0.0	0.0	0.0	0.0	0.0	0.0
	UB	3970.7	232.6	192.3	1686.1	178.7	681.0	6361.3	1652.4	13340.1

Table 11s. Predicted cumulative number of newly forecasted monkeypox cases and 95% PIs for the second-ranked model. Predicted cumulative number of newly forecasted monkeypox cases and 95% PIs for each of the 4-week ahead forecasts during the weeks of 7-28-2022 through 10-13-2022 based on a 10-week calibration period for the second-ranked sub-epidemic model.

Weighted - Ensemble Model										
Week		**Brazil**	**Canada**	**England**	**France**	**Germany**	**Spain**	**US (OWID)**	**US (CDC)**	**World**
07-28-22	Median	8463.7	742.3	773.9	872.4	591.2	1391.2	4838.8	2874.5	20957.3
	LB	6067.5	622.3	322.9	245.6	234.1	0.0	2038.2	1484.1	14263.4
	UB	11783.6	887.6	1384.3	2322.0	972.4	5137.4	17645.5	10437.6	32121.5
08-04-22	Median	1187.2	385.9	660.3	522.6	458.7	966.3	33042.3	35181.3	34936.8
	LB	651.6	215.0	224.7	62.0	167.6	0.0	21005.3	9062.0	24148.0
	UB	2841.9	621.8	1157.7	1316.6	767.2	3435.6	55553.7	149984.9	42706.4
08-11-22	Median	2685.3	254.5	434.9	286.1	420.3	1237.7	6058.4	4442.5	22930.0
	LB	1477.3	143.3	79.0	0.0	162.3	0.0	2242.7	2943.4	15153.2
	UB	5348.1	385.7	848.3	924.7	696.3	3754.3	20689.6	6676.0	32717.9
08-18-22	Median	1631.6	231.2	251.2	152.8	354.1	1158.6	6247.6	4667.2	18643.0
	LB	711.3	120.6	32.6	0.0	114.4	0.0	1816.2	2955.4	12818.4
	UB	3662.6	352.7	515.4	677.8	593.4	3503.7	15853.4	8601.9	25447.7
08-25-22	Median	2312.1	185.7	147.6	609.1	277.7	806.8	3420.0	3871.9	15412.5
	LB	1288.4	97.3	11.7	0.0	57.8	0.0	610.6	1938.8	9456.3
	UB	3903.2	277.4	340.1	2517.0	506.4	2753.8	7594.1	6232.0	22458.5
09-01-22	Median	1593.3	111.7	130.1	503.9	147.1	755.3	3824.4	3175.5	12899.5
	LB	768.7	8.0	9.9	0.0	0.0	0.0	571.0	1698.0	7794.2
	UB	2599.4	240.7	281.3	2252.0	410.0	2166.7	8101.4	4687.9	18540.1
09-08-22	Median	1723.7	75.6	108.4	438.6	56.7	271.0	2978.7	2542.6	9097.0
	LB	866.6	1.9	0.0	0.0	0.0	0.0	223.6	959.7	5279.6
	UB	2648.9	180.9	267.2	1998.6	158.0	933.0	6706.9	4174.2	13323.9
09-15-22	Median	2401.9	66.0	74.0	346.6	51.6	143.9	3826.9	2526.5	11764.0
	LB	844.3	0.0	0.0	0.0	0.0	0.0	690.5	999.4	5704.9
	UB	4116.9	415.6	733.7	1836.3	303.1	739.6	7379.2	4087.7	18545.3
09-22-22	Median	2144.9	41.7	79.9	357.9	12.8	143.3	2958.6	2011.8	10205.4
	LB	875.0	0.0	0.0	0.0	0.0	0.0	265.3	768.4	4305.2
	UB	3452.4	354.2	734.6	1882.9	236.1	663.5	6078.7	3299.8	16332.0
09-29-22	Median	1534.7	29.5	45.0	115.6	7.7	32.3	3365.1	1395.1	8853.4
	LB	320.1	0.0	0.0	0.0	0.0	0.0	326.2	377.7	3312.8
	UB	2898.0	223.8	701.1	1432.3	215.9	437.5	6758.5	2469.3	14514.1
10-06-22	Median	1117.8	13.6	20.0	32.7	4.3	66.1	1017.4	931.4	5297.9
	LB	129.8	0.0	0.0	0.0	0.0	0.0	0.0	330.9	244.2
	UB	2240.8	129.0	574.3	1229.4	131.4	335.6	5154.0	1551.8	11508.3
10-13-22	Median	878.9	14.2	2.7	10.3	1.5	43.0	1806.3	910.1	6231.5
	LB	25.2	0.0	0.0	0.0	0.0	0.0	0.0	245.3	492.8
	UB	1950.5	90.3	342.6	1087.6	125.6	315.5	5542.4	1603.3	12463.1

Table 12s. Predicted cumulative number of newly forecasted monkeypox cases and 95% PIs for the weighted ensemble model. Predicted cumulative number of newly forecasted monkeypox cases and 95% PIs for each 4-week ahead forecasts during the weeks of 7-28-2022 through 10-13-2022 based on a 10-week calibration period for the weighted sub-epidemic ensemble model.

Unweighted - Ensemble Model										
Week		Brazil	Canada	England	France	Germany	Spain	US (OWID)	US (CDC)	World
07-28-22	Median	9453.81	720.35	812.55	892.66	811.35	2501.29	3947.64	2787.79	20769.27
	LB	6136.76	583.03	251.00	154.97	286.08	83.57	1722.17	1467.06	13597.43
	UB	33425.20	866.54	1750.31	2298.95	2179.16	6289.50	14718.29	9995.46	31957.42
08-04-22	Median	1255.12	387.40	675.83	668.80	484.80	1301.58	21959.92	37056.90	33481.53
	LB	644.02	196.25	211.91	94.33	169.67	0.00	5763.36	9169.81	20344.07
	UB	2154.17	655.15	1314.96	1430.76	782.50	5118.04	53110.37	151064.53	61582.74
08-11-22	Median	3465.88	257.71	489.50	360.88	494.27	1556.86	7090.51	4526.81	21736.19
	LB	1630.85	134.47	73.84	0.00	153.42	0.00	2045.94	3008.05	15234.04
	UB	5086.97	390.58	1016.95	2905.87	1317.54	4640.71	23052.82	6832.69	31359.19
08-18-22	Median	1691.65	278.17	302.44	200.16	388.30	1898.78	7120.39	4791.23	17854.07
	LB	691.80	136.15	22.71	0.00	111.59	0.00	1552.00	2937.10	11341.66
	UB	3661.71	459.49	1339.64	1033.44	758.20	4211.14	29838.26	29020.30	25529.89
08-25-22	Median	2576.71	200.15	176.60	609.45	319.26	587.55	3807.32	3919.09	15401.01
	LB	1150.41	85.09	13.61	0.00	77.69	0.00	453.12	1989.57	9839.95
	UB	20773.99	808.69	451.66	4883.46	558.30	2537.73	9944.00	6237.37	22282.28
09-01-22	Median	1602.62	125.83	141.45	432.51	173.88	481.38	4827.79	3399.82	15298.59
	LB	706.05	2.07	1.95	0.00	0.00	0.00	428.49	1447.05	8529.28
	UB	2828.54	331.73	410.76	2643.14	630.89	1931.92	84582.47	16358.72	23416.86
09-08-22	Median	1966.47	89.40	138.00	406.53	70.80	307.24	3204.48	2692.46	9405.82
	LB	832.92	0.00	0.00	0.00	0.00	0.00	46.76	670.41	4754.03
	UB	14835.60	319.38	457.35	2483.85	277.94	1437.07	8769.74	9638.91	16766.10
09-15-22	Median	3133.06	88.63	136.83	332.19	74.15	267.11	5045.99	2832.23	15653.78
	LB	954.99	0.00	0.00	0.00	0.00	0.00	835.58	838.21	6437.87
	UB	11140.29	645.42	1366.06	2374.49	571.20	2121.99	230054.20	7442.24	42965.67

Table 13s. Predicted cumulative number of newly forecasted monkeypox cases and 95% PIs for the unweighted ensemble model. Predicted cumulative number of newly forecasted monkeypox cases and 95% PIs for each of the 4-week ahead forecasts during the weeks of 7-28-2022 through 9-15-2022 based on a 10-week calibration period for the unweighted sub-epidemic ensemble model. Only weeks in which the 4-week ahead forecast have past are included in this table.
